# Genome-Wide Relatedness of *Treponema pedis,* from Gingiva and Necrotic Skin Lesions of Pigs, with the Human Oral Pathogen *Treponema denticola*

**DOI:** 10.1371/journal.pone.0071281

**Published:** 2013-08-19

**Authors:** Olov Svartström, Memoona Mushtaq, Märit Pringle, Bo Segerman

**Affiliations:** 1 Department of Biomedical Sciences and Veterinary Public Health, Swedish University of Agricultural Sciences, Uppsala, Sweden; 2 Department of Animal Breeding and Genetics, Swedish University of Agricultural Sciences, Uppsala, Sweden; 3 Department of Animal Health and Antimicrobial Strategies, National Veterinary Institute, Uppsala, Sweden; 4 Department of Bacteriology, National Veterinary Institute, Uppsala, Sweden; Federal University of Pelotas, Brazil

## Abstract

*Treponema pedis* and *T. denticola* are two genetically related species with different origins of isolation. *Treponema denticola* is part of the human oral microbiota and is associated with periodontitis while *T. pedis* has been isolated from skin lesions in animals, *e.g.,* digital dermatitis in cattle and necrotic ulcers in pigs. Although multiple *Treponema* phylotypes may exist in ulcerative lesions in pigs, *T. pedis* appears to be a predominant spirochete in these lesions. *Treponema pedis* can also be present in pig gingiva. In this study, we determined the complete genome sequence of *T. pedis* strain T A4, isolated from a porcine necrotic ear lesion, and compared its genome with that of *T. denticola*. Most genes in *T. pedis* were homologous to those in *T. denticola* and the two species were similar in general genomic features such as size, G+C content, and number of genes. In addition, many homologues of specific virulence-related genes in *T. denticola* were found in *T. pedis*. Comparing a selected pair of strains will usually not give a complete picture of the relatedness between two species. We therefore complemented the analysis with draft genomes from six *T. pedis* isolates, originating from gingiva and necrotic ulcers in pigs, and from twelve *T. denticola* strains. Each strain carried a considerable amount of accessory genetic material, of which a large part was strain specific. There was also extensive sequence variability in putative virulence-related genes between strains belonging to the same species. Signs of lateral gene-transfer events from bacteria known to colonize oral environments were found. This suggests that the oral cavity is an important habitat for *T. pedis*. In summary, we found extensive genomic similarities between *T. pedis* and *T. denticola* but also large variability within each species.

## Introduction

The genus *Treponema* includes commensal and pathogenic spirochete species, some of which affect human and animal health. These fastidious bacteria often require an anaerobic environment and are difficult to grow and manipulate *in vitro*. Some species, *e.g. T. pallidum*, are host dependent and have not been successfully cultivated on bacteriological media. The fastidiousness of these bacteria has hampered *Treponema* research. Consequently, few *Treponema* pathogenic mechanisms have been well characterized.

Our research focuses on characterization of porcine skin lesions that are colonized, and perhaps worsened, by *Treponema* [1], [2], [3]. One treponeme of particular interest is *T. pedis*. The *T. pedis* type strain T3552B^T^ originates from a bovine digital dermatitis (BDD) lesion [4]. In pigs, *T. pedis* has been isolated from gingiva and necrotic ulcers, referred to as ear necrosis and shoulder ulcers [2], [3]. Ear necrosis and shoulder ulcers are necrotic skin lesions of animal welfare concern. Ear necrosis can cause loss of the entire ear in severe cases and shoulder ulcers can develop into deep necrotic lesions involving underlying bone tissue. A recent study by Karlsson et al., showed that *T. pedis*, several other *Treponema* phylotypes, and coccoid bacteria are frequently occurring in these lesions [1]. Common coccoid bacteria in skin lesions in pigs are *Staphylococcus hyicus* and β-hemolytic streptococci [5].

The human oral microbiome is currently under investigation and DNA from 11 classified species of *Treponema* has been found [6]. One of these, *T. denticola,* is associated with human periodontitis. Overgrowth of bacteria from the species *T. denticola, Porphyromonas gingivalis*, and *Tannerella forsythia*, often collectively referred to as the “red complex”, is believed to be associated with the clinical progression of periodontitis [7]. The genome sequence of *T. denticola* strain ATCC 35405 was released in 2004 [8], providing a resource for identifying virulence factors.

*Treponema pedis* and *T. denticola* are phylogenetically close based on 16S rRNA gene comparison. Originally, *T.* pedis was described as *T. denticola*-like since these species share 95.7% 16S similarity [4]. To our knowledge, *T. denticola* has only been isolated from oral samples in humans. In contrast, *T. pedis* has been isolated from oral locations (gingiva of pigs) and lesions (ear necrosis, shoulder ulcers, and BDD) demonstrating that this is a treponeme capable of colonizing both the oral cavity and skin lesions.

To gain insight into the genetic composition of *T. pedis* and to identify potential virulence factors, we determined the complete genome sequence of the *T. pedis* strain T A4, isolated from a case of pig ear necrosis. From the comparative genome analysis we discovered that *T. pedis* shares substantial genetic similarities, including conserved virulence-related genes, with *T. denticola*. The *T. pedis* T A4 reference genome was complemented with a dataset of six draft whole-genome shotgun (WGS) assemblies representing *T. pedis* isolates from ear necrosis, shoulder ulcer and gingiva of pigs. The wide set of *T. pedis* genes was compared to genes in *T. denticola* ATCC 35405 complemented with genes from 12 additional *T. denticola* draft WGS assemblies. These were made from data deposited in the NCBI Sequence Read Archive (http://www.ncbi.nlm.nih.gov/sra). This analysis enabled us to make an estimation of the pan-genome structures of *T. pedis* and *T. denticola*, determine the sequence variability of specific virulence-related genes, and trace lateral gene-transfer events.

## Materials and Methods

### Isolation and Culturing

Seven *T. pedis* isolates from porcine gingiva, ear necrosis, and shoulder ulcers were used in this study (Table S1). These were chosen for sequencing as they represents *T. pedis* isolated from lesions and gingiva. All clinical isolates used in this study originates from studies approved by the ethical committee of animal experiments in Uppsala, Sweden. The isolation procedure was the same as described for *T. pedis* strain T A4 [2]. Culturing was done anaerobically at 37°C on a shaker (90 rpm) in fastidious anaerobe broth (LAB 71, LabM, UK, Lancashire) containing the following additives per liter: 2.0 g D-glucose (Amresco, USA, OH, Solon); 720 µg thiamine pyrophosphate; 10 µl each of isobutyric acid, isovaleric acid, 2-methylbutyric acid, valeric acid (solubilized in 0.1 M KOH); and 25% fetal calf serum (S 0115, Biochrom AG, Germany, Berlin).

### DNA Preparation and Sequencing

The *T. pedis* strain T A4 was used to produce a complete reference genome for the species by a combination of sequencing techniques. Genomic DNA from T A4 was prepared using a DNeasy Blood & Tissue Kit (Qiagen, http://www.qiagen.com) starting with 30 ml of a 5-day culture. Before the DNA was prepared, cultures were inspected not to have non-spirochetal contamination by phase-contrast microscopy. The DNA was sequenced on a Roche 454 GS FLX platform (DNAVision, Belgium, Gosselies) using one-fourth of a titanium plate; this yielded 310,432 reads with an average length of 408 bp. PCR generated sequencing templates for gap closure and verified low coverage and areas of poor quality in the assembly. The PCR products were purified (Illustra GFX PCR Purification Kit, GE Healthcare, www.gehealthcare.com) and analyzed by Sanger sequencing on an ABI 3730XL instrument (Macrogen Europe, Netherlands, Amsterdam). To further improve the quality of the assembly, DNA from 80 ml of a 4-day broth culture was extracted by phenol/chloroform and sequenced on an Illumina HiSeq 2000 instrument (Macrogen Inc, Korea, Seoul). The Illumina paired-end reads (2×100 bp) were used to error correct the 454/Sanger derived genome sequence.

Additional *T. pedis* isolates were sequenced on an Illumina MiSeq instrument (2×250 bp, paired end). DNA was prepared using the same method as for the 454 sequencing but starting with 10 ml of broth culture. One ng DNA, as determined on a Qubit fluorometer (Invitrogen, NY, Grand Island), was used with a Nextera XT (Illumina, http://www.illumina.com) sequencing-library preparation kit, according to the manufacturer’s instructions.

The Illumina reads (2×100 bp) that were used to assemble draft genome sequences from 12 *T. denticola* strains (Table S2) were downloaded from GenBank, SRA (http://www.ncbi.nlm.nih.gov/sra) and converted to FASTQ format using the SRA toolkit (NCBI). Raw data for *T. pedis* have been deposited in SRA and can be accessed via the bioproject page (bioprojects numbers are listed in table S1 and S2).

### Genome Assemblies

An initial draft-genome assembly consisting of 53 contigs for *T. pedis* T A4 with 40× coverage was produced from the 454 reads using a GS *de novo* assembler (Roche). The contigs were joined in Consed [9] and confirmed with PCR. Problematic areas were identified using search functions and manual inspection in Consed. Some low quality areas had to be broken up, reassembled and verified by PCR. One gap was solved by primer walking over a long-range PCR product. In total, 85 Sanger sequenced PCR products were added. Since no widespread insertion elements, only two rRNA operons and limited number of repetitive regions were present, most gaps could be closed relatively easy. Homopolymers, indels, and other minor errors (minor misassemblies) were corrected by mapping 30,012,760 high-coverage Illumina paired-end reads to the final gap-free assembly. The high sequence depth also ensured high coverage over the whole genome. The starting codon of the chromosomal replication initiator *dnaA* gene on the leading strand was designated as the sequence starting point.

Draft genome *de novo* assemblies of remaining *T. pedis* isolates and *T. denticola* strains were produced using Mira3 [10]. The completed genome of *T. denticola* strain ATCC 35405 was downloaded from GenBank (accession AE017226). The assemblies derived from the available SRA data for *T. denticola* strains SP23, SP32 and SP44 resulted in consensus sequence twice the size of the genome of *T. denticola* ATCC 35405 and showed an uneven distribution of contig coverage (data not shown) suggesting they were not pure; therefore they were not included in this study.

### Annotation of *T. pedis* T A4

Coding DNA sequence (CDS) positions in *T. pedis* T A4 were predicted with Glimmer 3 [11]. Positions of the ribosomal subunit (rRNA) genes 16S, 23S and 5S were identified by pair-wise nucleotide comparisons with corresponding genes in *T. denticola* ATCC 35405. Transfer RNA (tRNA) genes were predicted using tRNAscan [12]. All CDSs that overlapped at least 50% with a tRNA, rRNA or another CDS were removed. The set of predicted reference genes in *T. pedis* T A4 were given locus tags ranging from TPE0001 to TPE2809.

A homology search of translated CDSs in *T. pedis* T A4 was performed using BLASTP (NCBI) [13]. The CDSs were compared to the proteins in the all-bacterial genome database (ftp://ftp.ncbi.nlm.nih.gov/genomes/Bacteria/all.gbk.tar.gz). Functional annotation was assigned to a CDS based on the best BLASTP hit where >30% amino acid identity was observed in the alignment, length difference was <25% and e-value was <1×10^−6^. All other CDSs were annotated as hypothetical proteins. Borderline cases were manually inspected. A distribution of different functional categories for the CDSs in *T. pedis* T A4 and *T. denticola* ATCC 35405 were made by performing a BLASTP search in the COG-database (clusters of orthologous groups) [14]. The functional category was retrieved from groups where e-value was ≤1×10^−5^.

### Clustering Analysis

The CDSs in the draft genome sequences were predicted with Glimmer 3 [11]. The translated CDSs in all genomes, including the reference strains (*T. pedis* T A4 and *T. denticola* ATCC 35405), were subjected to all-against-all sequence similarity comparisons using BLASTP. The *T. denticola* ATCC 35405 genes marked as “pseudo” were also included. Clusters of homologues were constructed in parallel for both species by collapsing intraspecies CDSs sharing >80% amino acid identity in the BLASTP alignment and with lengths deviating <30%. Functional annotation information for the clusters was retrieved by querying all translated CDS members in the all-bacterial genome database (including *T. pedis* T A4). The annotation from the best-hit gene was transferred, along with the score.

### Phylogenetic Analysis, Amino Acid Alignments and Whole Genome Comparisons

Phylogenetic analysis, using the Neighbor joining method, of the intergenic spacer region between the 16S rRNA and tRNA^Ile^ genes and amino acid alignments were done in the CLC Main Work Bench 6 software (CLC bio). Whole-genome average genomic similarities were calculated in Gegenees [15]. Genome alignment between *T. pedis* T A4 and *T. denticola* ATCC 35405 was made with Mummer [16]. Circular representation of the genome was made in DNAplotter [17].

## Results

### The Complete Genome Sequence of *T. pedis* Shares General Features with *T. denticola*

**Figure 1:**
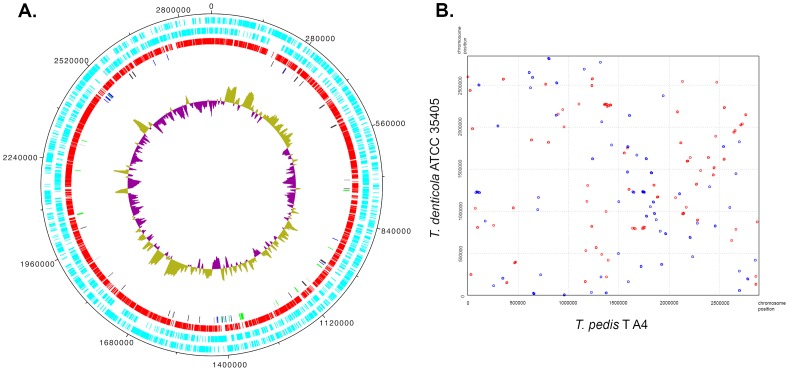
Circular representation of the *T. pedis* T A4 genome and complete genome alignment with *T. denticola*. (A.) Circular representation of the *T. pedis* T A4 genome. The CDSs are shown in violet where the outer circle represents predictions on the plus strand and the second circle those on the minus strand. CDSs with a best BLASTP hit in *T. denticola* ATCC 35405 are colored red and shown in the third circle. The fourth circle represents genes with best BLASTP hits in *T. brennaborense* (black), *F. nucleatum* (green), *F. alocis* (blue) and *T. succinifaciens* (grey). G+C skew is drawn in the inner circle. (B.) Complete genome alignment between *T. pedis* T A4 and *T. denticola* ATCC 35405. Dots represent maximum unique matches (MUMs) between the genomes. MUMs oriented in the same direction are depicted as red dots and reverse complemented MUMs are depicted as blue dots.

The genome of *T. pedis* strain T A4 (accession CP004120) consisted of a single 2,889,325 base pair (bp) circular replicon (Figure 1A) with a G+C content of 36.9%. There were 2,806 predicted CDSs with an average length of 755 bp. The longest CDS encoded a putative lipoprotein and comprised 6,261 nucleotides. There were two copies of the rRNA operon organized as 16S-tRNA-23S-5S and 45 predicted tRNA genes. There were paralogous variants of the 23S and 16S rRNA genes with single-nucleotide differences. One of the 16S rRNA genes was identical with the deposited sequence of the *T. pedis* type strain T3552B^T^ (NR_044064).

The full-length 16S rRNA genes from *T. pedis* T A4 and *T. denticola* ATCC 35405 shared 96% sequence identity and the general features of the genomes were also very similar (Table 1). Overall, *Treponema pedis* and *T. denticola* showed similar profiles of distribution between functional categories (Table 2) representing most functional groups in the COG-database [14]. *Treponema pedis* had 38% more energy production related genes. For both species, approximately 60% of the CDSs had unknown or poorly characterized functions. A comparison with BLASTP found that most of the CDSs (2,077 CDSs, i.e., ∼74% of them) were more closely related to proteins of *T. denticola* than to any other proteins in the all-completed bacterial genomes (Figure 1A, circle 3). Among the CDSs that did not give a best hit in *T. denticola*, 31 showed highest similarity with proteins from *Treponema brennaborense*, 27 with proteins from *Fusobacterium nucleatum*, 19 with proteins from *Filifactor alocis,* and 14 with proteins from *Treponema succinifaciens* (Figure 1A, circle 4). There were also a small number of gene products that showed highest similarity with proteins from *Dichelobacter nodosus*, *Tannerella forsythia,* and *Porphyromonas gingivalis*. In contrast to the gene content, synteny was only weakly conserved between *T. pedis* T A4 and *T. denticola* ATCC 35405 (Figure 1B).

**Table 1:** General genomic features of *T. pedis* strain T A4 and *T. denticola* strain ATCC 35405.

	*T. pedis* strain T A4	*T. denticola* strain ATCC 35405
**Size**	2,889,325 bp	2,843,201 bp
**G+C content**	36.9%	37.9%
**Protein-coding genes**	2,806	2,786
**rRNA operons**	2	2
**tRNAs**	45	44

**Table 2:** Distribution of functional categories in *T. pedis* strain T A4 and *T. denticola* strain ATCC 35405 according to the COG classification.

	Number of CDSs			
Functional category	*T. pedis* TA4		*T. denticola* ATCC 35405	
Function unknown	1476	(52.6%)	1433	(51.4%)
General function prediction only	208	(7.4%)	229	(8.2%)
Translation, ribosomal structure and biogenesis	135	(4.8%)	143	(5.1%)
Cell wall/membrane/envelope biogenesis	121	(4.3%)	95	(3.4%)
Amino acid transport and metabolism	100	(3.6%)	100	(3.6%)
Energy production and conversion	90	(3.2%)	65	(2.3%)
Inorganic ion transport and metabolism	89	(3.2%)	83	(3.0%)
Defense mechanisms	87	(3.1%)	99	(3.6%)
Replication, recombination and repair	78	(2.8%)	93	(3.3%)
Carbohydrate transport and metabolism	68	(2.4%)	67	(2.4%)
Posttranslational modification, protein turnover, chaperones	61	(2.2%)	59	(2.1%)
Transcription	59	(2.1%)	79	(2.8%)
Coenzyme transport and metabolism	52	(1.9%)	51	(1.8%)
Nucleotide transport and metabolism	44	(1.6%)	44	(1.6%)
Signal transduction mechanisms	40	(1.4%)	49	(1.8%)
Lipid transport and metabolism	39	(1.4%)	39	(1.4%)
Cell motility	24	(0.9%)	26	(0.9%)
Cell cycle control, cell division, chromosome partitioning	19	(0.7%)	16	(0.6%)
Intracellular trafficking, secretion, and vesicular transport	14	(0.5%)	13	(0.5%)
Secondary metabolites biosynthesis, transport and catabolism	2	(0.1%)	3	(0.1%)

In *T. denticola* ATCC 35405, there are four, long genes located in pairs (TDE1558 (3,320 aa), TDE1560 (1,126 aa), TDE2020 (1,140 aa) and TDE2022 (1,488 aa)) that match the YD repeat motif (TIGR01643), meaning that they are possibly involved in attachment to carbohydrate structures. In *T. pedis* T A4, there were two corresponding regions but instead of containing two long genes each, they contained a high number (17 and 20) of smaller gene predictions homologous to the *T. denticola* YD repeat proteins. This probably reflects non-functionalization and we therefore designated these gene predictions as pseudogenes. In *T. pedis* T A4, there were 70 gene products with predicted functions as ABC permease transporters. The corresponding number in *T. denticola* ATCC 35405 is 83 [6]. Four *T. pedis* T A4 gene products were predicted to function as hemolysins. No plasmids, evident IS elements or prophages were found.

### Assembly Properties and Phylogeny of *T. pedis* and *T. denticola* Draft Genomes

**Figure 2:**
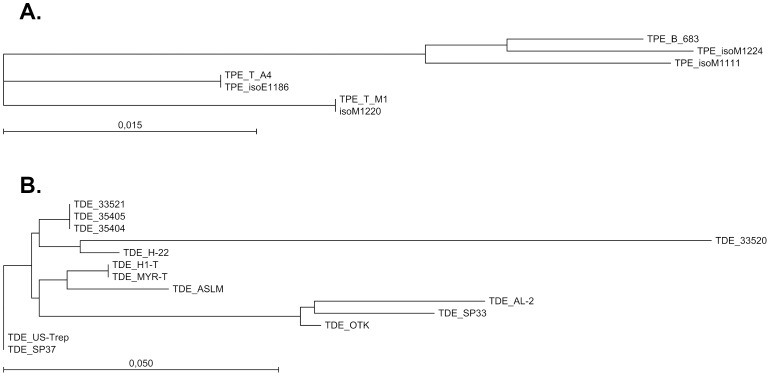
Phylogeny of *T. pedis* isolates and *T. denticola* strains. Phylogeny based on neighbor-joining of the intergenic spacer region between the 16S rRNA and tRNA^Ile^. The sequences were extracted from the WGS assemblies generated in this study. (A) Phylogeny of the *T. pedis* isolates. (B) Phylogeny of the *T. denticola* strains. The bars corresponds to 0.015 and 0.050 nucleotide substitutions per position.

Six draft genomes of *T. pedis* isolates were generated with assembly sizes varying between 2.95 and 3.47 Mbp and with G+C contents varying between 36.9% and 37.3% (Table S1). In addition, twelve *T. denticola* draft genomes were assembled from SRA data with sizes varying between 2.76 and 3.03 Mbp and with G+C contents varying between 37.7% and 38.0% (Table S2). Phylogeny, based on the variable intergenic spacer region between the 16S and tRNA^Ile^ genes, showed heterogeneity among the *T. pedis* isolates (Figure 2A) and *T. denticola* strains (Figure 2B), although a few had identical sequences in this region. However, based on whole-genome average similarity, as measured with the Gegenees software [12], no genomes were identical. The pairwise, intraspecies average similarities on a nucleotide level were in the range of 78% – 99.7% in the conserved core.

Within the phylogeny of *T. pedis*, the lesion-derived isolates did not form a specific genotypic group. In the clustering analysis of all *T. pedis* genes described below, only six gene clusters were found exclusively in lesion isolates (clusters 0827, 1328, 1344, 1509, 2834 and 2855; Table S3).

### Pan-genome Structures of *T. pedis* and *T. denticola*

**Figure 3:**
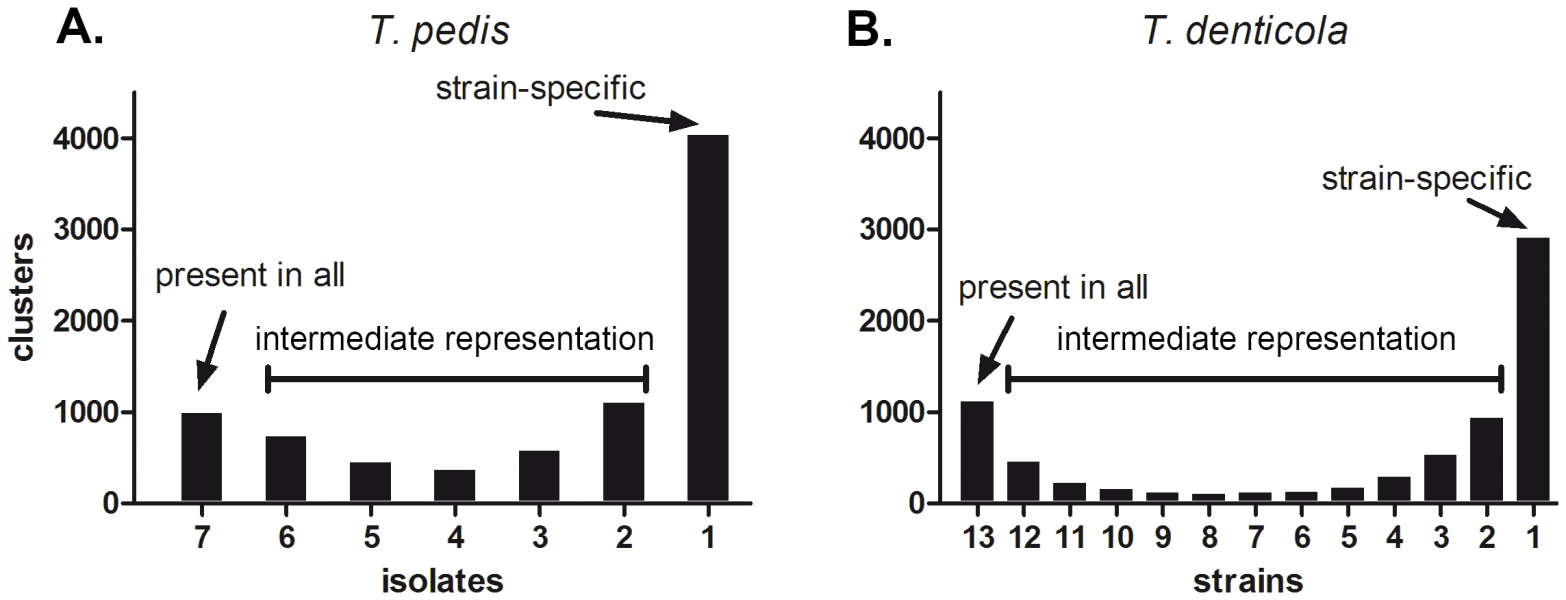
Quantification of strain-specific, intermediately-represented, and core genes in *T. pedis* and *T. denticola*. The distribution between strain-specific genes, intermediately-represented genes, and genes present in all genomes, i.e., core functions, in the analyzed genome datasets of *T. pedis* (A) and *T. denticola* (B). The gene representation was determined by a clustering analysis that collapsed CDSs sharing >80% BLASTP identity and that had <30% length difference.

All CDSs in the reference genomes and draft WGS assemblies (File S1) were used to estimate the variability in the pan-genomes for the two species. We produced clusters of intraspecies homologues by collapsing similar CDSs, i.e., those sharing >80% amino acid identity in the alignment and with lengths deviating <30%. Functional annotation information was acquired from the best BLASTP hits in the all-bacterial genome database. This resulted in 8,244 clusters in *T. pedis* (Table S3) and 7,269 in *T. denticola* (Table S4). On the basis of their high sequence identity and similar size, we assumed that the genes in a single cluster represented a specific function. Clusters that included CDSs from all isolates/strains were presumed to represent core functions. The clustering analysis enabled us to quantify the core genes, the strain-specific genes, and the genes with intermediate representation within the draft genome dataset. The relative distribution between these categories was similar for the two species (Figure 3). There were 988 core functions in *T. pedis* and 1,115 in *T. denticola*. On average, each *T. pedis* isolate contributed with 576 strain-specific clusters. In *T. denticola*, the corresponding number was 224.

Many of the clusters represent related functions. We therefore wanted to quantify the number of completely novel clusters with which the draft genome analysis contributed. We identified 758 clusters in the *T. pedis* draft assemblies that did not give any BLASTP hit in *T. pedis* T A4 (Table S5). The *T. denticola* draft assemblies contributed 977 clusters with no BLASTP hit in *T. denticola* ATCC 35405 (Table S6).

Next, we searched for genes that may have been acquired from recent, lateral DNA-transfer events. We looked specifically in the dataset of strain-specific genes assuming they represent more recently acquired genes that have not yet or will not become a part of the core genome. Thus, we examined possible gene exchanges between *T. pedis* and *T. denticola* by identifying strain-specific genes with an intraspecies BLASTP score <80 and a score >160 in a core function of the compared species. The limitation to core functions was used to lower the probability that the identified genes originated from other species. In *T. pedis* there were three strain-specific genes that fulfilled these criteria (Clusters 2118, 6126 and 7527; Table S3) and two in *T. denticola* (Clusters 0207 and 6387; Table S4).

Gene exchange from other species was examined by analyzing strain-specific genes with BLASTP scores <80 in all other *T. pedis* and *T. denticola* genomes. Among these, 41 strain-specific genes in *T. pedis* (Table S7) and 42 in *T. denticola* (Table S8) had BLASTP hit scores >160 in the all-bacterial genome database. In *T. pedis,* putative lateral gene transfer events from related species of oral origin occurred, including *F. alocis* and *P. gingivalis*. Similarly for *T. denticola*, there were putative lateral gene transfer events from *e.g. T. forsythia* and *F. alocis*.

### Potential Virulence Factors in *T. pedis*

Many of the putative virulence factors described in *T. denticola* were also found in *T. pedis* T A4 (Table 3). The putative *T. pedis* T A4 major surface-sheath protein (Msp), aligned with relatively low amino-acid identity (29%) with the *T. denticola* ATCC 35405 Msp protein. All components of the dentilisin operon (prcB, prcA and prtP) were found in *T. pedis* TA4 and had amino acid identities of 48%, 56%, and 67%, respectively when aligned to the corresponding *T. denticola* ATCC 35405 proteins. There were also homologues in *T. pedis* T A4 to a *T. denticola* surface antigen (TDE2258), and three *T. denticola* proteases (TDE1195, TDE2140 and TDE0362). A high degree of conservation was observed for *T. denticola* ATCC 35405 filament protein (TDE0842) and flagellar hook protein (TDE2768) when comparing them with the corresponding genes in *T. pedis* T A4. There were no homologues in *T. pedis* T A4 to the *T. denticola* ATCC 35405 virulence related *fhbB* (TDE0108) and *oppA* (TDE1071) genes.

**Table 3:** Putative virulence factors.

Locus tag			
*T. denticola* ATCC 35405	*T. pedis* T A4	Amino acid identity	Gene product
TDE0405	TPE2758	29%	major outer sheath protein
TDE0760/0761/0762	TPE1952/1951/1950	48/56/67%	dentilisin components
TDE2258	TPE1291	54%	surface antigen
TDE0842	TPE0043	90%	filament protein
TDE2768	TPE0609	91%	flagellar hook protein
TDE2140	TPE0746	72%	Protease
TDE1195	TPE0822	75%	Protease
TDE0362	TPE1565	26%	Protease

Virulence-related genes in *T. denticola* and their *T. pedis* homolouges.

The putative virulence-related genes in the completed genomes described above were used as references to determine the conservation across the set of draft genomes from *T. pedis* (Table S9) and *T. denticola* (Table S10).

In *T. denticola*, filament protein A (TDE0842), flagellar hook protein (TDE2768), and two proteases (TDE2140 and TDE1195) showed high similarity to each other throughout their entire sequences. In *T. pedis*, the corresponding genes were also highly similar to each other, but the conservation was in many cases limited to only a part of the protein.

There was lower sequence variability in the dentilisin components of *T. pedis* compared to *T. denticola.* In the *T. denticola* strain SP33, only short sequence fragments with low similarities were found matching the dentilisin components. This indicates that the strain may have lost this function.

The surface antigen protein and the Msp product had high sequence variability in both species, *e.g.* the Msp of *T. denticola* strain OTK showed only 31% identity to that of *T. denticola* strain ATCC 35405.

Protease activity is dependent on catalytic residues at an active site. Specific residues have been described for the *T. denticola* proteases PtrB (TDE2140) [18], Dentipain (TDE0362) [19] and PrtP (TDE0762) [20]. We also identified potential catalytic residues in the PtrB paralogue TDE1195 [7] by alignment with PtrB. These four proteases were used as reference to align the identified *T. pedis* and *T. denticola* homologues. The catalytic residues in PtrB (Figure S1), the PtrB paralogue (Figure S2), Dentipain (Figure S3) and PrtP (Figure S4) were conserved in *T. pedis* strain T A4 (Table 4). In the draft genomes, most predicted genes showed conservation over the active site, including catalytic residues. However, in all four alignments, there were occurrences of sequences that lacked regions with catalytic residues (Figure S1, S2, S3, S4).

**Table 4:** Proteases identified in *T. pedis* T A4 and their putative catalytic residues.

Description	Locus tag	Putative catalytic residues
PtrB (Oligopeptidase)	TPE0746	Ser 571, Asp 656 and His 691
PtrB paralogue (Oligopeptidase)	TPE0822	Ser 538, Asp 622 and His 657
Dentipain (IgG-specific protease)	TPE1565	Cys 315 and His 463
PrtP (dentilisin component)	TPE1950	Asp 192, His 246 and Ser 435

## Discussion

Animal health in pig production is important both from an animal welfare perspective and for economical profit. Pigs with ear necrosis may be difficult to sell and sows with shoulder ulcers can cause economical losses due to early slaughter. The role of treponemes in necrotic pig ulcers is still poorly understood. This study analyzed genome sequences of *T. pedis* isolates from pig gingiva, ear necrosis, and shoulder ulcer. These were compared to multiple strains of the closely-related, human oral pathogen, *T. denticola*. Here we describe their relatedness on the genomic level and identify putative virulence-related genes in *T. pedis*.

Shared general genomic features, similar functional class distributions and having a majority of gene content homologous demonstrates a close genetic relation between *T. pedis* and *T. denticola*. The low level of gene synteny indicates that a large amount of genome re-arrangements have occurred since these organisms diverged. Low gene synteny has also been reported between *T. denticola* ATCC 35405 and *T. pallidum* (NC_000919) [8]. However, large contigs (>10 kbp) from draft genomes of *T. pedis* and *T. denticola* aligned with high synteny against the completed genomes within their species (data not shown). The putative genes in *T. pedis* T A4 include the relatively high number of ABC-transporter genes in *T. denticola* ATCC 35405 suggested to be used for competitive growth [8] and several hemolysins that may cause tissue destruction.

Specific discrepancies between *T. pedis* and *T. denticola* were also found. The *T. pedis* genome contained 38% more energy-production related genes which may contribute to a potential of colonizing a broader range of habitats. The YD-repeat genes in *T. denticola* ATCC 35405 were found in seemingly degenerated forms in *T. pedis* T A4 and we could not identify homologues to the serum resistance associated *fhbB* gene (TDE0108) [21], [22] and *oppA* gene (TDE1071) that binds host proteins [23].

When we examined the pan-genomes of *T. pedis* and *T. denticola,* strain-specific genes with no homology within genomes of the same species were identified. We find it likely that these genes are signs of putative lateral-gene transfer events from the surrounding microbiota. However, there was little evidence of recent gene transfer events between *T. pedis* and *T. denticola*, which would be expected from species that colonize different hosts. The genome of *T. pedis* T A4 contained several CDSs that were most closely related to genes from the periodontal-associated species *P. gingivalis*, *T. forsythia* [7], and *F. alocis* [24]. Presence of these species and *T. pedis* have been demonstrated in tonsils of pigs [25], [26] indicating that gene transfer between these species may occur in the oral cavity. In addition, there were also CDSs most closely related to genes from the digital dermatitis-associated species *T. brennaborense* [27] and *D. nodosus*. Co-existence of *D. nodosus* and treponemes has been demonstrated both in ovine digital dermatitis [28] and BDD [29]. The genome of *D. nodosus,* where the homology occurred, has been characterized using an isolate from ovine foot rot [30]. Collectively, these data indicate that *T. pedis* acquires genes laterally from species associated with both skin diseases and periodontitis.

In addition to colonize different hosts, *T. pedis* seems to colonize a wider range of habitats than *T. denticola*, including both the oral cavity and necrotic ulcers. The fact that *T. pedis* was first isolated from a lesion [31] does not exclude the possibility that it is a species originating from an oral environment which extended its habitat to skin lesions. There were no indications that gingiva and skin lesions of pigs were colonized by different *T. pedis* genotypes. Clusters containing genes solely from isolates originating from lesions were few and the annotations did not point to any obvious relation to virulence. Thus, it is likely that transmission between the oral environment and skin is mediated by biting behavior.

Strains within a bacterial species typically share a set of conserved core genes, with each strain containing a variable number of accessory genes. The total gene set of a species is often referred to as its pan-genome [32]. By clustering closely related genes we observed a similar distribution for *T. pedis* and *T. denticola* between core genes, intermediately represented genes, and genes found in a single strain. The core genomes of *T. pedis* and *T. denticola* both consisted of approximately 1,000 different gene functions. Each strain carried a considerable number of accessory genes where a large proportion of them were strain specific. The draft genome analysis contributed with many completely novel genes to the pan-genomes, which emphasizes the importance of complementing a reference genome with draft genomes to understand the species.

The gene homologies in *T. pedis* and *T. denticola* enabled us to identify a set of potential virulence-related genes in *T. pedis*. The degree of conservation between the putative virulence genes in *T. pedis* and *T. denticola* varied considerably.

There were homologues in *T. pedis* T A4 to a *T. denticola* ATCC 35405 surface antigen (TDE2258) involved in co-aggregration with the oral species *T. forsythia* [33] and motility genes indirectly involved in biofilm formation with other bacteria (filament protein, flagellar hook protein) [7] were highly conserved. This suggests that *T. pedis* interacts with other bacteria similarly as *T. denticola*.

A set of *T. denticola* protease homologues was identified in *T. pedis* T A4. These include the dentilisin operon in *T. denticola*, which is believed to contribute to periodontal disease by interfering with host signaling pathways and degrade host-cell matrix proteins by its proteolytic activity [7], [33]. In *T. denticola* ATCC 35405, this operon consists of the components named PrcB, PrcA and PrtP [34]. The PrtP component is responsible for the proteolytic activity of dentilisin [20]. We could not identify an intact dentilisin operon in *T. denticola* strain SP33; this suggests that the virulence of this strain may be impaired. There were also homologues in *T. pedis* to the two PtrB oligopeptidases that have been proposed as virulence factors in *T. denticola* [7], [18]. Finally, a homologue to an IgG-specific protease designated as dentipain in *T. denticola* was found in *T. pedis* T A4. Dentipain-deficiant mutants of *T. denticola* have been shown to cause smaller absesses in a murine model [19]. The catalytic residues in all these proteases were found in the homologues in *T. pedis* strain T A4, strongly suggesting the activity is conserved which may contribute to virulence.

The major surface sheath protein (Msp) is abundant in the cell membrane of *T. denticola.* It has drawn much attention as a potential virulence determinant because it mediates colonization through binding to host proteins. It can also act as a porin with cytopathic effects [7], [33]. A high degree of variability was observed in Msp proteins from both *T. denticola* and *T. pedis*. The Msp of *T. pedis* showed only 29% identity to that in *T. denticola*. However, the identity within the species of *T. denticola* varied down to 31% which indicates a variable nature of this gene. Sequence variations of Msp in *T. denticola* have previously been reported both in *T. denticola* strains [35] and in Msp sequences derived from clinical periodontal samples [36].

In conclusion, *T. pedis* and *T. denticola* are genetically similar to each other when comparing whole genome sequences. The affiliation of *T. pedis* with the oral microbiota is supported by its homology with other oral species and by indications of gene exchange with these species. This study has demonstrated variability, both in gene content and in specific genes, among strains of these species. This emphasizes the importance of complementing a completed reference genome with draft sequences from additional strains. The homology between *T. pedis* and *T. denticola* was used to identify potential genetic traits. These traits will be the subject of future research to understand the role of *T. pedis* in necrotic skin lesions.

## Supporting Information

Figure S1 **Amino acid alignments of** ***T. denticola*** **ATCC 35405 protease PtrB (TDE2140) and identified homologues.** Described catalytic residues are indicated along with their corresponding positions in TDE2140.
(PDF)

Figure S2 **Amino acid alignments of** ***T. denticola*** **ATCC 35405 PtrB protease homologue (TDE1195) and identified homologues.** Described catalytic residues are indicated along with their corresponding positions in TDE1195.
(PDF)

Figure S3 **Amino acid alignments of** ***T. denticola*** **ATCC 35405 protease Dentipain (TDE0362) and identified homologues.** Described catalytic residues are indicated along with their corresponding positions in TDE0362.
(PDF)

Figure S4 **Amino acid alignments of** ***T. denticola*** **ATCC 35405 protease PrtP (TDE0762) and identified homologues.** Described catalytic residues are indicated along with their corresponding positions in TDE0762.
(PDF)

Table S1 Assembly information for draft genomes of *T. pedis* isolates. (XLS)

Table S2 Assembly information for draft genomes of *T. denticola* strains. (XLS)

Table S3 Presence in isolates. (XLS)

Table S4 Presence in strains. (XLS)

Table S5 (XLS)

Table S6 (XLS)

Table S7 (XLS)

Table S8 (XLS)

Table S9 Virulence gene conservation in *T. pedis*. Comparison of the putative virulence-related genes in *T. pedis* TA 4 with those in the *T. pedis* draft genomes. The amino-acid identity values in the alignment are shown and the alignment coverage is in brackets, if below 70%.
(XLS)

Table S10 Virulence gene conservation in *T. denticola.*
Comparison of the virulence related genes in *T. denticola* ATCC 35405 with those in the *T. denticola* draft genomes. The amino-acid identity values in the alignment are shown and the alignment coverage is in brackets, if below 70%.
(XLS)

File S1 Contains .faa file. (ZIP)
