# Supplementary material for: Genome-Wide Relatedness of Treponema pedis, from Gingiva and Necrotic Skin Lesions of Pigs, with the Human Oral Pathogen Treponema denticola
Source: PLoS One. 2013 Aug 19;8(8):e71281. doi: 10.1371/journal.pone.0071281 (PMC3747143; doi:10.1371/journal.pone.0071281)
Supplement: Figure S1 — Amino acid alignments of T. denticola ATCC 35405 protease PtrB (TDE2140) and identified homologues. Described catalytic residues are indicated along with their corresponding positions in TDE2140. (PDF) [file pone.0071281.s001.pdf]

```
                                   20                  40                  60                  80
TDE_35405       ----------------------------------LKQSDFEKPPIAEIKETRFEKFGKTRIDNYYWLKDKTDKKVIDYLNAENAYTDKIMASSKDLQK
TDE_33520       ----------------------------------LKQSDFEKPPIAEIKETRFEKFGKTRIDNYYWLKDKTDKKVIDYLNAENAYTDKIMASSKDLQK
TDE_33521       ----------------------------------LKQSDFEKPPIAEIKETRFEKFGKTRIDNYYWLKDKTDKKVIDYLNAENAYTDKIMASSKDLQK
TDE_35404       ----------------------------------LKQSDFEKPPIAEIKETRFEKFGKTRIDNYYWLKDKTDKKVIDYLNAENAYTDKIMASSKDLQK
TDE_AL-2        ----------------------------------LKQSDFEKPPIAEIKETRFEKFGKTRIDNYYWLKDKTDKKVIDYLNAENAYTDKIMASSKDLQK
TDE_ASLM        ----------------------------------LKQSDFEKPPVAEIKETRFEKFGKTRIDNYYWLKDKTDKKVIDYLNAENAYTDKIMASSKDLQK
TDE_H-22        ----------------------------------LKQSDFEKPPIAEIKETRFEKFGKTRIDNYYWLKDKTDKKVIDYLNAENAYTDKIMASSKDLQK
TDE_H1-T        --------------------------------------------------------------------------------------------------
TDE_MYR-T       ----------------------------------LKQSDFEKPPIAEIKETRFEKFGKTRIDNYYWLKDKTDKKVIDYLNAENAYTDKIMASSKDLQK
TDE_OTK         ----------------------------------LKQSDFEKPPVAEIKETRFEKFGKTRIDNYYWLKDKTDKKVIDYLNAENAYTDKIMASSKDLQK
TDE_SP33        ----------------------------------LKQSDFEKPPVAEIKETRFEKFGKTRIDNYYWLKDKTDKKVIDYLNAENAYTDKIMAFSKDLQK
TDE_SP37        ----------------------------------LKQSDFEKPPIAEIKETRFEKFGKTRIDNYYWLKDKTDKKVIDYLNAENAYTDKIMASSKDLQK
TDE_US-Trep     ----------------------------------LKQSDFEKPPIAEIKETRFEKFGKTRIDNYYWLKDKTDKKVIDYLNAENAYTDKIMASSKDLQK
TPE_TA4         MLKTKNYVKVLPAILTAGLSVFLGCQTGQSKSVQPKQSDFEAPPKAQIYPEEFTKFGNKRVDNYYWLKDKNNPEVIDYLKAENAYTDTVMASTKDLQK
TPE_TM1         MLKTKNYVKVLPAILTAGLSVFLGCQTGQSKSVQPKQSDFEAPPKAQIYPEEFTKFGNKRVDNYYWLKDKNNPEVIDYLKAENAYTDTVMASTKDLQK
TPE_B683        MLKTKNSVKVLPAILTAGLSVFLGCQTGQSKSVQPKQSDFEAPPKAQIHPEEFTKFGNKRVDNYYWLKDKNNPEVIDYLKAENAYTDTVMASTKDLQK
TPE_isoM1111    --------------------------------------------------------------------------------------------------
TPE_isoE1186    MLKTKNSVKVLPAILTAGLSVFLGCQTGQSKSVQPKQSDFEAPPKAQIHPEEFTKFGNKRVDNYYWLKDKNNPEVIDYLKAENAYTDSVMASTKDLQK
TPE_isoM1220    MLKTKNYVKVLPAILTAGLSVFLGCQTGQSKSVQPKQSDFEAPPKAQIYPEEFTKFGNKRVDNYYWLKDKNNPEVIDYLKAENAYTDTVMASTKDLQK
TPE_isoM1224    MLKTKNSVKVLPAILTAGLSVFLGCQTGQSKSVQPKQSDFEAPPKAQIHPEEFTKFGNKRVDNYYWLKDKKNPEVIDYLKAENAYTDSVMASTKDLQK
```

```
             100                 120                 140                 160                 180
TDE_35405    SIYDEIVGRIKEDDETYPVFENGYYYYNRVEKGKQYRTYCRKKASLDAAEEIIFDVNKMAEGKQAFIFDDYVVSPDNKKACYFYNETGSFAEFILKIR
TDE_33520    SIYDEIVGRIKEDDETYPVFENGYYYYNRVEKGKQYRTYCRKKASLDAAEEIIFDVNKMAEGKQAFIFDDYVVSPDNKKACYFYNETGSFAEFILKIR
TDE_33521    SIYDEIVGRIKEDDETYPVFENGYYYYNRVEKGKQYRTYCRKKASLDAAEEIIFDVNKMAEGKQAFIFDDYVVSPDNKKACYFYNETGSFAEFILKIR
TDE_35404    SIYDEIVGRIKEDDETYPVFENGYYYYNRVEKGKQYRTYCRKKASLDAAEEIIFDVNKMAEGKQAFIFDDYVVSPDNKKACYFYNETGSFAEFILKIR
TDE_AL-2     SIYDEIVGRIKEDDETYPVFENGYYYYNRVEKGKQYRTYCRKKASLDAAEEIIFDVNKMAEGKQAFIFSDYVVSPDNKKACYFYNETGSFAEFILKIR
TDE_ASLM     SIYDEIVGRIKEDDETYPVFKNGYYYYNRVEKGKQYRTYCRKKASLNAAEEIIFDVNKMAEGKQAFIFSDYVVSPDNKKACYFYNETGSFAEFILKIR
TDE_H-22     SIYDEIVGRIKEDDETYPIFENGYYYYNRVEKGKQYRTYCRKKASLDAAEEIIFDVNKMAEGKQAFIFSDYVVSPDNKKACYFYNETGSFAEFILKIR
TDE_H1-T     ----------------------------------------------------------MAEGKQAFIFDDYVVSPDNKKACYFYNETGSFAEFILKIR
TDE_MYR-T    SIYDEIVGRIKEDDETYPVFENGYYYYNRVEKGKQYRTYCRKKASLDAAEEIIFDVNKMAEGKQAFIFDDYVVSPDNKKACYFYNETGSFAEFILKIR
TDE_OTK      SIYDEIVGRIKEDDETYPVFENGYYYYNRVEKGKQYRTYCRKKASLDAAEEIIFDVNKMAEGKQAFIFSDYVVSPDNKKACYFYNETGSYAEFILKIR
TDE_SP33     SIYDEIVGRIKEDDETYPVFENGYYYYNRVEKGKQYRTYCRKKASLDAAEEIIFDVNKMAEGKQAFIFDDYVVSPDNKKACYFYNETGSFAEFILKIR
TDE_SP37     SIYDEIVGRIKEDDETYPVFENGYYYYNRVEKGKQYRTYCRKKASLDAAEEIIFDVNKMAEGKQAFIFSDYVVSPDNKKACYFYNETGSFAEFILKIR
TDE_US-Trep  SIYDEIVGRIKEDDETYPVFENGYYYYNRVEKGKQYRTYCRKKASLDAAEEIIFDVNKMAEGKQAFIFSDYVVSPDNKKACYFYNETGSFAEFILKIR
TPE_TA4      TLYDEMIARIKEDDESYPVFRNGYYYYSRVEKGKQYRVYCRKKGSLEAKEEIFFDVNKMAEGKNAFIFSRYSVSPDNTKAIYFYNETGSFAEFTMKIR
TPE_TM1      TLYDEMIARIKEDDESYPVFRNGYYYYSRVEKGKQYRVYCRKKGSLEAKEEIFFDVNKMAEGKNAFIFSRYSVSPDNTKAIYFYNETGSFAEFTMKIR
TPE_B683     TLYDEMIARIKEDDESYPVFRNGYYYYSRVEKGKQYRVYCRKKGSLEAEEEIFFDVNKMAEGKNAFIFSRYSVSPDNTKAIYFYNETGSFAEFTMKIR
TPE_isoM1111 --------------------------------------------------------------------------------------------------
TPE_isoE1186 TLYDEMIARIKEDDESYPVFRNGYYYYSRVEKGKQYRVYCRKKGSLEAEEEIFFDVNKMAEGKNAFIFSRYSVSPDNTKAIYFYNETGSFAEFTMKIR
TPE_isoM1220 TLYDEMIARIKEDDESYPVFRNGYYYYSRVEKGKQYRVYCRKKGSLEAKEEIFFDVNKMAEGKNAFIFSRYSVSPDNTKAIYFYNETGSFAEFTMKIR
TPE_isoM1224 TLYDEMIARIKEDDESYPVFRNGYYYYSRVEKGKQYRVYCRKKGSLEAEEEIFFDVNKMAEGKNAFIFSRYSVSPDNTKAIYFYNETGSFAEFTMKIR
```

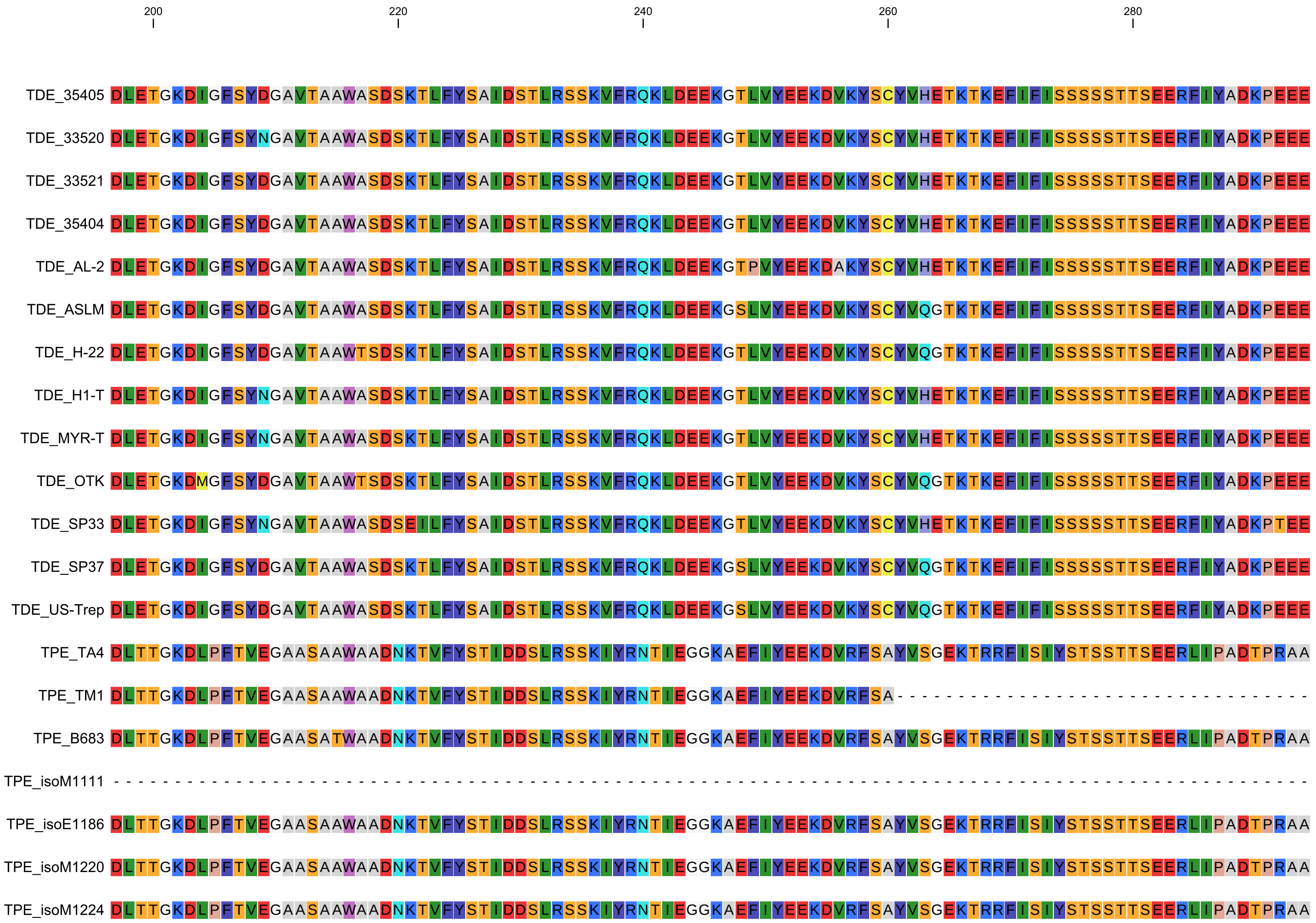
200
220
240
260
280
TDE_35405 DLETGKDIGFSYDGAVTAAWASDSKTLFYSAIDSTLRSSKVFRQKLDEEKGTLVYEEKDVKYSCYVHETKTKEFIFISSSSSTTSEERFIYADKPEEE
TDE_33520 DLETGKDIGFSYNGAVTAAWASDSKTLFYSAIDSTLRSSKVFRQKLDEEKGTLVYEEKDVKYSCYVHETKTKEFIFISSSSSTTSEERFIYADKPEEE
TDE_33521 DLETGKDIGFSYDGAVTAAWASDSKTLFYSAIDSTLRSSKVFRQKLDEEKGTLVYEEKDVKYSCYVHETKTKEFIFISSSSSTTSEERFIYADKPEEE
TDE_35404 DLETGKDIGFSYDGAVTAAWASDSKTLFYSAIDSTLRSSKVFRQKLDEEKGTLVYEEKDVKYSCYVHETKTKEFIFISSSSSTTSEERFIYADKPEEE
TDE_AL-2 DLETGKDIGFSYDGAVTAAWASDSKTLFYSAIDSTLRSSKVFRQKLDEEKGTPVYEEKDAKYSCYVHETKTKEFIFISSSSSTTSEERFIYADKPEEE
TDE_ASLM DLETGKDIGFSYDGAVTAAWASDSKTLFYSAIDSTLRSSKVFRQKLDEEKGSLVYEEKDVKYSCYVQGTKTKEFIFISSSSSTTSEERFIYADKPEEE
TDE_H-22 DLETGKDIGFSYDGAVTAAWTSDSKTLFYSAIDSTLRSSKVFRQKLDEEKGTLVYEEKDVKYSCYVQGTKTKEFIFISSSSSTTSEERFIYADKPEEE
TDE_H1-T DLETGKDIGFSYNGAVTAAWASDSKTLFYSAIDSTLRSSKVFRQKLDEEKGTLVYEEKDVKYSCYVHETKTKEFIFISSSSSTTSEERFIYADKPEEE
TDE_MYR-T DLETGKDIGFSYNGAVTAAWASDSKTLFYSAIDSTLRSSKVFRQKLDEEKGTLVYEEKDVKYSCYVHETKTKEFIFISSSSSTTSEERFIYADKPEEE
TDE_OTK DLETGKDMGFSYDGAVTAAWTSDSKTLFYSAIDSTLRSSKVFRQKLDEEKGTLVYEEKDVKYSCYVHETKTKEFIFISSSSSTTSEERFIYADKPEEE
TDE_SP33 DLETGKDIGFSYNGAVTAAWASDSEILFYSAIDSTLRSSKVFRQKLDEEKGTLVYEEKDVKYSCYVHETKTKEFIFISSSSSTTSEERFIYADKPTEE
TDE_SP37 DLETGKDIGFSYDGAVTAAWASDSKTLFYSAIDSTLRSSKVFRQKLDEEKGSLVYEEKDVKYSCYVQGTKTKEFIFISSSSSTTSEERFIYADKPEEE
TDE_US-Trep DLETGKDIGFSYDGAVTAAWASDSKTLFYSAIDSTLRSSKVFRQKLDEEKGSLVYEEKDVKYSCYVQGTKTKEFIFISSSSSTTSEERFIYADKPEEE
TPE_TA4 DLTTGKDLPFTVEGAASAAWAADNKTVFYSTIDDSLRSSKIYRNTIEGGKAEFIYEEKDVRFSAYVSGEKTRRFISIYSTSSTTSEERLIPADTPRAA
TPE_TM1 DLTTGKDLPFTVEGAASAAWAADNKTVFYSTIDDSLRSSKIYRNTIEGGKAEFIYEEKDVRFSA------------------------------------
TPE_B683 DLTTGKDLPFTVEGAASATWAADNKTVFYSTIDDSLRSSKIYRNTIEGGKAEFIYEEKDVRFSAYVSGEKTRRFISIYSTSSTTSEERLIPADTPRAA
TPE_isoM1111 --
TPE_isoE1186 DLTTGKDLPFTVEGAASAAWAADNKTVFYSTIDDSLRSSKIYRNTIEGGKAEFIYEEKDVRFSAYVSGEKTRRFISIYSTSSTTSEERLIPADTPRAA
TPE_isoM1220 DLTTGKDLPFTVEGAASAAWAADNKTVFYSTIDDSLRSSKIYRNTIEGGKAEFIYEEKDVRFSAYVSGEKTRRFISIYSTSSTTSEERLIPADTPRAA
TPE_isoM1224 DLTTGKDLPFTVEGAASAAWAADNKTVFYSTIDDSLRSSKIYRNTIEGGKAEFIYEEKDVRFSAYVSGEKTRRFISIYSTSSTTSEERLIPADTPRAA

```
                 300                 320                 340                 360                 380
TDE_35405      FKIFLPRVKDTEYSVYPHKEKFFIRYKDKQNLNGKIYSAPRSSYSDKSTWKEERAHDENVRIEDVSVFESYLVLELRKNGLIEIEIKSLKNGEVKNIS
TDE_33520      FKIFLPRVKDTEYSVYPHKEKFFIRYKDKQNLNGKIYSAPRSSYSDKSTWKEERAHDENVRIEEVSVFESYLVLELRKNGLIEIEIKSLKNGEVKNIS
TDE_33521      FKIFLPRVKDTEYSVYPHKEKFFIRYKDKQNLNGKIYSAPRSSYSDKSTWKEERAHDENVRIEDVSVFESYLVLELRKNGLIEIEIKSLKNGEVKNIS
TDE_35404      FKIFLPRVKDTEYSVYPHKEKFFIRYKDKQNLNGKIYSAPRSSYSDKSTWKEERAHDENVRIEDVSVFESYLVLELRKNGLIEIEIKSLKNGEVKNIS
TDE_AL-2       FKIFLPRVKDTEYSVYPHKEKFFIRYKDKQNLNGKIYSAPRSSYSDKSTWKEERAHDENVRIEEVSVFESYLVLELRKNGLIEIEIKSLKNGEVKNIS
TDE_ASLM       FKIFLPRVKDTEYSVYPHKEKFFIRYKDKQNLNGKIYSAPRSSYSDKSTWKEERAHDENVRIEDVSVFESYLVLELRKNGLIEIEIKSFKNGEVKNIS
TDE_H-22       FKIFLPRVKDTEYSVYPHKEKFFIRYKDKQNLNGKIYSAPRSSYSDKSTWKEERAHDENVRIEALSVFESYIVLELRKNGLIEIEIKSLKNGEVKNIS
TDE_H1-T       FKIFLPRVKDTEYSVYPHKEKFFIRYKDKQNLNGKIYSAPRSSYSDKSTWKEERAHDENVRIEEVSVFESYLVLELRKNG------------EVKNIS
TDE_MYR-T      FKIFLPRVKDTEYSVYPHKEKFFIRYKDKQNLNGKIYSAPRSSYSDKSTWKEERAHDENVRIEEVSVFESYLVLELRKNG------------EVKNIS
TDE_OTK        FKIFLPRVKDTEYSVYPHKEKFFIRYKDKQNLNGKIYSAPRSSYSDKSTWKEERAHDENVRIEDVSVFESYLVLELRKNGLIEIEIKSLKNGEVKNIS
TDE_SP33       FKIFLPRVKDTEYSVYPHKEKFFIRYKDKQNLNGKIYSSPRSSYSDKSTWKEERAHDENVRIEALSVFESYIVLELRKNGLIEIEIKSLKNGEVKNIS
TDE_SP37       FKIFLPRVKDTEYSVYPHKEKFFIRYKDKQNLNGKIYSAPRSSYSDKSTWKEERAHDENVRIEDVSVFESYLVLELRKNGLIEIEIKSFKNGEVKNIS
TDE_US-Trep    FKIFLPRVKDTEYSVYPHKEKFFIRYKDKQNLNGKIYSAPRSSYSDKSTWKEERAHDENVRIEDVSVFESYLVLELRKNGLIEIEIKSFKNGEVKNIS
TPE_TA4        FTVFKPRVQDIDYSVIPHKDCFFIQYKDKENLNGKLYKAPLTGFENTANWQEIVPHNPDVRIEGIDIFKDFLVLELRKNGLIEIKILSIADGTEKNIA
TPE_TM1        --------------------------------------------------------------------------------------------------
TPE_B683       FTVFKPRVQDIDYSVIPHKDCFFIQYKDKENLNGKLYKAPLTGFENTANWQEVVPHNPDVRIEGIDIFKDFLVLELRKNGLIEIKILSIADGSEKSIS
TPE_isoM1111   --------------------------KDKENLNGKLYKAPLTGFENTANWQEIVPHNPDVRIEGIDIFKDFLVLELRKNGLIEIKILSIADGSEKNIA
TPE_isoE1186   FTVFKPRVQDIDYSVIPHKDCFFIQYKDKENLNGKLYKAPLTGFENTANWQEIVPHNPDVRIEGIDIFKDFLVLELRKNGLIEIKILSIADGSEKNIA
TPE_isoM1220   FTVFKPRVQDIDYSVIPHKDCFFIQYKDKENLNGKLYKAPLTGFENTANWQEIVPHNPDVRIEGIDIFKDFLVLELRKNGLIEIKILSIADGTEKNIA
TPE_isoM1224   FTVFKPRVQDIDYSVIPHKDCFFIQYKDKENLNGKLYKAPLTGFENTANWQEIVPHNPDVRIEGIDIFKDFLVLELRKNGLIEIKILSIADGSEKNIA
```

```
                 400                 420                 440                 460                 480
                   |                   |                   |                   |                   |
TDE_35405    FPEPVYTAYLGANPEYVSDKVRYIYTSLNRPSSVYDYDILTGKSVLLKQQEVPSGFNPDDYTVERLWATAQDGVKVPMAAVYKKGLVKDGSSPALLYS
TDE_33520    FPEPVYTAYLGANPEYSSDKVRYIYTSLNRPSSVYDYDILTGKSVLLKQQEVPSGFNPDDYTVERLWATAQDGVKVPMAAVYKKGLXK----------
TDE_33521    FPEPVYTAYLGANPEYVSDKVRYIYTSLNRPSSVYDYDILTGKSVLLKQQEVPSGFNPDDYTVERLWATAQDGVKVPMAAVYKKGLVKDGSSPALLYS
TDE_35404    FPEPVYTAYLGANPEYVSDKVRYIYTSLNRPSSVYDYDILTGKSVLLKQQEVPSGFNPDDYTVERLWATAQDGVKVPMAAVYKKGLVKDGSSPALLYS
TDE_AL-2     FPEPVYTAYLGANPEYSSDKVRYIYTSLNRPSSVYDYDILTGKSVLLKQQEVPSGFNPDDYTVERLWATAQDGVKVPMAAVYKKGLVKDGAAPALLYS
TDE_ASLM     FPEPVYTAYLGANPEYSSDKVRYIYTSLNRPSSVYDYDILTGKSVLLKQQEVPSGFNPDDYTVERLWATAQDGVKVPMAAVYKKGLVKDGSAPALLYS
TDE_H-22     FPEPVYTAYLGANPEYSSDKVRYIYTSLNRPSSVYDYDILTGKSVLLKQQEVPSGFNPDDYTVERLWATAQDGVKVPMAAVYKKGLVKDGAAPALLYS
TDE_H1-T     FPEPVYTAYLGANPEYSSDKVRYIYTSLNRPSSVYDYDILTGKSVLLKQQEVPSGFNPDDYTVERLWATAQDGVKVPMAAVYKKGLVKDGSAPALLYS
TDE_MYR-T    FPEPVYTAYLGANPEYSSDKVRYIYTSLNRPSSVYDYDILTGKSVLLKQQEVPSGFNPDDYTVERLWATAQDGVKVPMAAVYKKGLVKDGSAPALLYS
TDE_OTK      FPEPVYTAYLGANPEYASDKVRYIYTSLNRPSSVYDYDILTGKSVLLKQQEVPSGFNPDDYTVERLWATAQDGVKVPMAAVYKKGLVKDGSAPALLYS
TDE_SP33     FPEPVYTAYLGANPEYSSDKVRYIYTSLNRPSSVYDYDILTGKSVLLKQQEVPSGFNPDDYTVERLWAKAQDGVKVPMAAVYKKGLAKDGSAPALLYS
TDE_SP37     FPEPVYTAYLGANPEYVSDKVRYIYTSLNRPSSVYDYDILTGKSVLLKQQEVPSGFNPDDYTVERLWATAQDGVKVPMAAVYKKGLVKDGSAPALLYS
TDE_US-Trep  FPEPVYTAYLGANPEYVSDKVRYIYTSLNRPSSVYDYDILTGKSVLLKQQEVPSGFNPDDYTVERLWATAQDGVKVPMAAVYKKGLVKDGSAPALLYS
TPE_TA4      FPEPVYTASLSGNPEYDAETIRYTYSSLNRPQTLYEYTIGTGKTEKLKEQEIPSGFNPDNYTVERLWAQAPDGVKVPMAVVYKKGLKKNGSAPALLYS
TPE_TM1      --------------------------------------------------------------------------------------------------
TPE_B683     FPEPVYTASLSGNPEYDAETIRYTYSSLNRPQTLYEYTIGTGKTEKLKEQEIPSGFNPDNYTVERLWAQAPDGVKVPMAVVYKKGLKKNGSAPALLYS
TPE_isoM1111 FPEPVYTASLSGNPEYDAETIRYTYSSLNRPQTLYEYTIGTGKTEKLKEQEIPSGFNPDNYTVERLWAQAPDGVKVPMAVVYKKGLKKNGSAPALLYS
TPE_isoE1186 FPEPVYTASLSGNPEYDAETIRYTYSSLNRPQTLYEYTIGTGKTEKLKEQEIPSGFNPDNYTVERLWAQAPDGVKVPMAVVYKKGLKKNGSAPALLYS
TPE_isoM1220 FPEPVYTASLSGNPEYDAETIRYTYSSLNRPQTLYEYTIGTGKTEKLKEQEIPSGFNPDNYTVERLWAQAPDGVKVPMAVVYKKGLKKNGSAPALLYS
TPE_isoM1224 FPEPVYTASLSGNPEYDAETIRYTYSSLNRPQTLYEYTIGTGKTEKLKEQEIPSGFNPDNYTVERLWAQAPDGVKVPMAVVYKKGLKKNGSAPALLYS
```

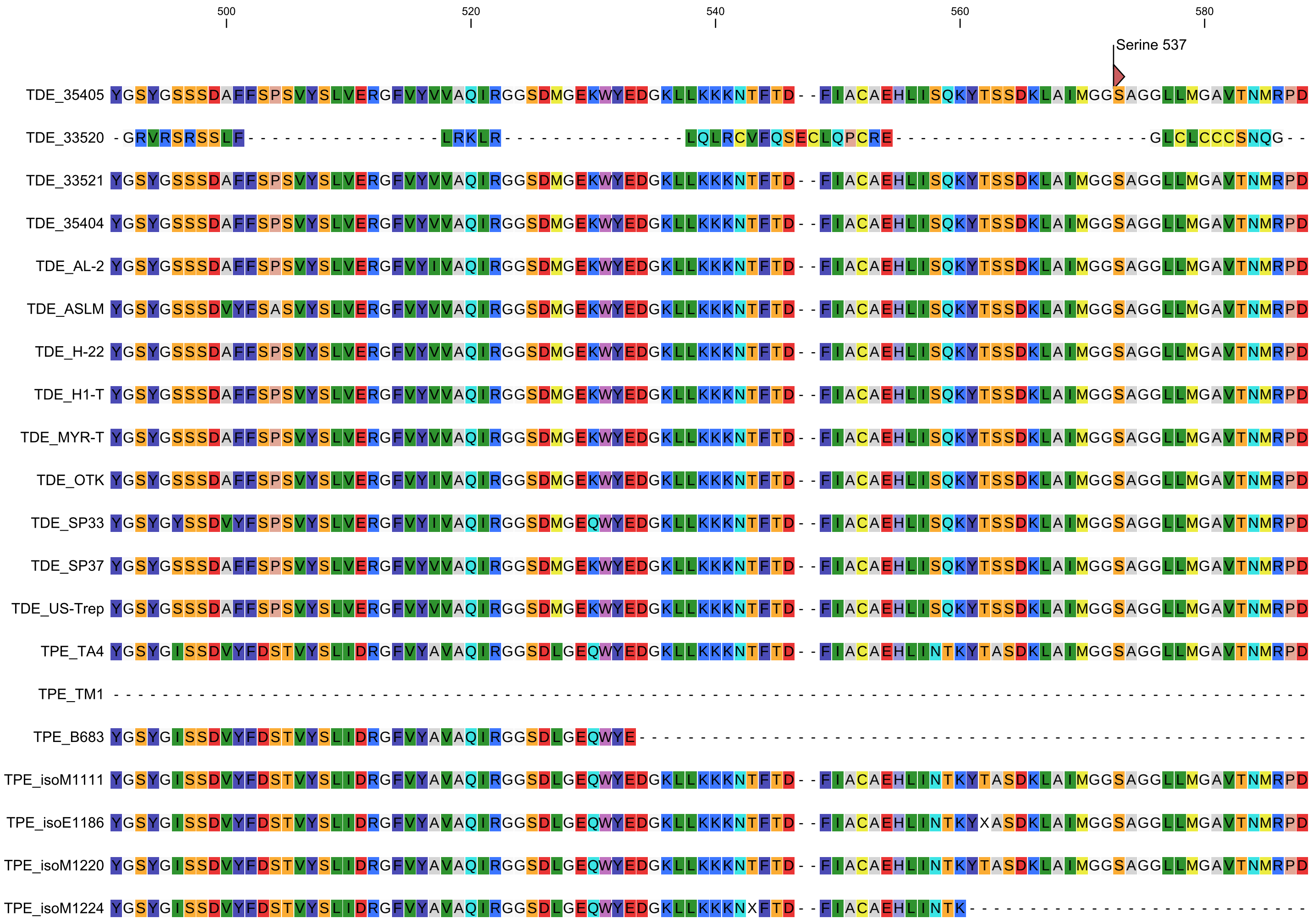
500
520
540
560
580
Serine 537
TDE_35405
TDE_33520
TDE_33521
TDE_35404
TDE_AL-2
TDE_ASLM
TDE_H-22
TDE_H1-T
TDE_MYR-T
TDE_OTK
TDE_SP33
TDE_SP37
TDE_US-Trep
TPE_TA4
TPE_TM1
TPE_B683
TPE_isoM1111
TPE_isoE1186
TPE_isoM1220
TPE_isoM1224

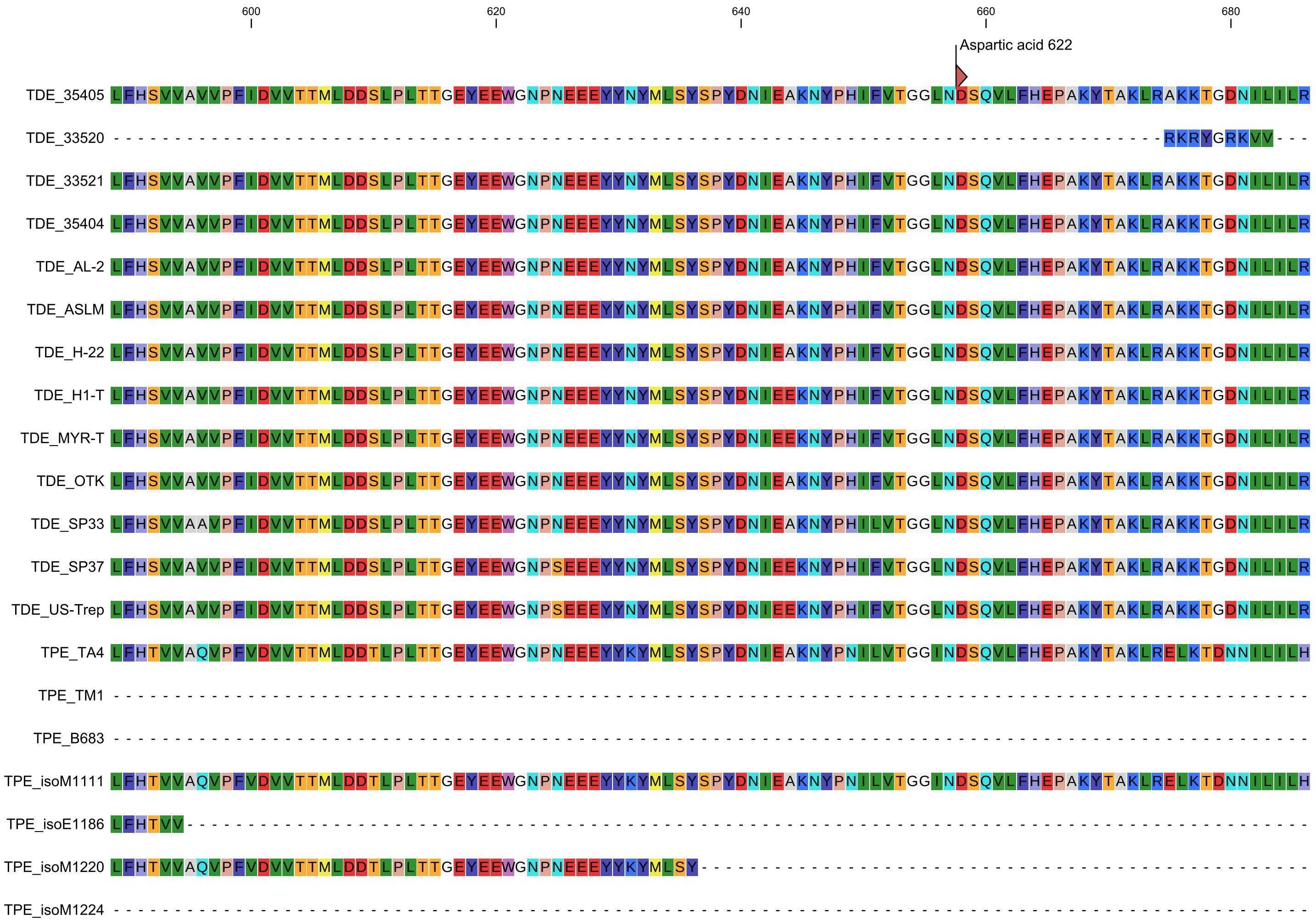
600
620
640
660
680
Aspartic acid 622
TDE_35405 LFHSVVAVVPFIDVVTTMLDDSLPLTTGEYEEWGNPNEEEYYNYMLSYSPYDNIEAKNYPHIFVTGGLNDSQVLFHEPAKYTAKLRAKKTGDNILILR
TDE_33520 RKRYGRKVV
TDE_33521 LFHSVVAVVPFIDVVTTMLDDSLPLTTGEYEEWGNPNEEEYYNYMLSYSPYDNIEAKNYPHIFVTGGLNDSQVLFHEPAKYTAKLRAKKTGDNILILR
TDE_35404 LFHSVVAVVPFIDVVTTMLDDSLPLTTGEYEEWGNPNEEEYYNYMLSYSPYDNIEAKNYPHIFVTGGLNDSQVLFHEPAKYTAKLRAKKTGDNILILR
TDE_AL-2 LFHSVVAVVPFIDVVTTMLDDSLPLTTGEYEEWGNPNEEEYYNYMLSYSPYDNIEAKNYPHIFVTGGLNDSQVLFHEPAKYTAKLRAKKTGDNILILR
TDE_ASLM LFHSVVAVVPFIDVVTTMLDDSLPLTTGEYEEWGNPNEEEYYNYMLSYSPYDNIEAKNYPHIFVTGGLNDSQVLFHEPAKYTAKLRAKKTGDNILILR
TDE_H-22 LFHSVVAVVPFIDVVTTMLDDSLPLTTGEYEEWGNPNEEEYYNYMLSYSPYDNIEAKNYPHIFVTGGLNDSQVLFHEPAKYTAKLRAKKTGDNILILR
TDE_H1-T LFHSVVAVVPFIDVVTTMLDDSLPLTTGEYEEWGNPNEEEYYNYMLSYSPYDNIEEKNYPHIFVTGGLNDSQVLFHEPAKYTAKLRAKKTGDNILILR
TDE_MYR-T LFHSVVAVVPFIDVVTTMLDDSLPLTTGEYEEWGNPNEEEYYNYMLSYSPYDNIEEKNYPHIFVTGGLNDSQVLFHEPAKYTAKLRAKKTGDNILILR
TDE_OTK LFHSVVAVVPFIDVVTTMLDDSLPLTTGEYEEWGNPNEEEYYNYMLSYSPYDNIEAKNYPHIFVTGGLNDSQVLFHEPAKYTAKLRAKKTGDNILILR
TDE_SP33 LFHSVVAAVPFIDVVTTMLDDSLPLTTGEYEEWGNPNEEEYYNYMLSYSPYDNIEAKNYPHILVTGGLNDSQVLFHEPAKYTAKLRAKKTGDNILILR
TDE_SP37 LFHSVVAVVPFIDVVTTMLDDSLPLTTGEYEEWGNPSEEEYYNYMLSYSPYDNIEEKNYPHIFVTGGLNDSQVLFHEPAKYTAKLRAKKTGDNILILR
TDE_US-Trep LFHSVVAVVPFIDVVTTMLDDSLPLTTGEYEEWGNPSEEEYYNYMLSYSPYDNIEEKNYPHIFVTGGLNDSQVLFHEPAKYTAKLRAKKTGDNILILR
TPE_TA4 LFHTVVAQVPFVDVVTTMLDDTLPLTTGEYEEWGNPNEEEYYKYMLSYSPYDNIEAKNYPNILVTGGINDSQVLFHEPAKYTAKLRELKTDNNILILH
TPE_TM1
TPE_B683
TPE_isoM1111 LFHTVVAQVPFVDVVTTMLDDTLPLTTGEYEEWGNPNEEEYYKYMLSYSPYDNIEAKNYPNILVTGGINDSQVLFHEPAKYTAKLRELKTDNNILILH
TPE_isoE1186 LFHTVV
TPE_isoM1220 LFHTVVAQVPFVDVVTTMLDDTLPLTTGEYEEWGNPNEEEYYKYMLSY
TPE_isoM1224

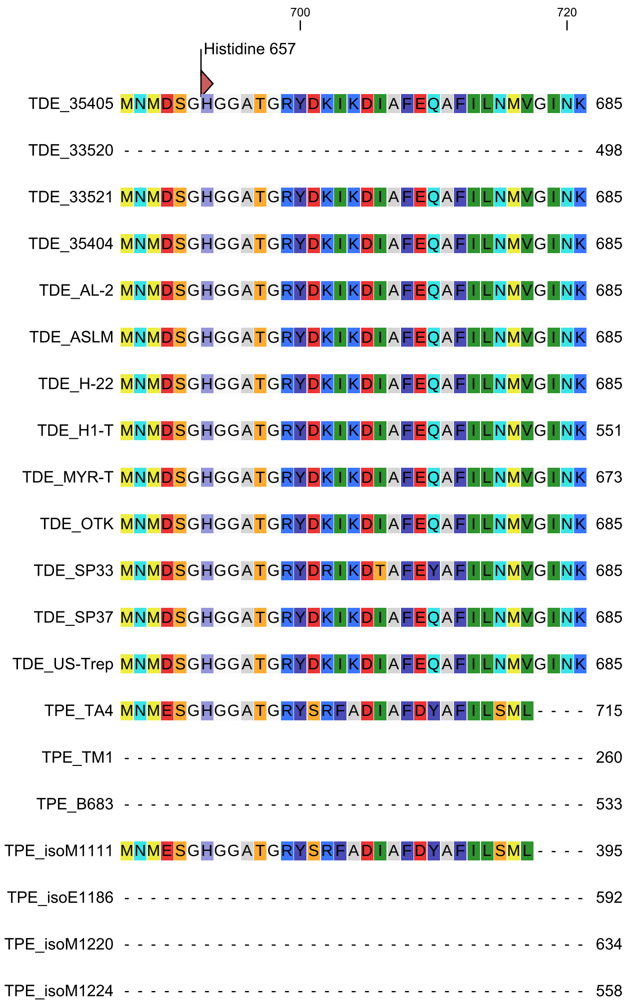
700
720
Histidine 657
TDE_35405 MNMDSGHGGATGRYDKIKDIAFEQAFILNMVGINK 685
TDE_33520 ----------------------------------- 498
TDE_33521 MNMDSGHGGATGRYDKIKDIAFEQAFILNMVGINK 685
TDE_35404 MNMDSGHGGATGRYDKIKDIAFEQAFILNMVGINK 685
TDE_AL-2 MNMDSGHGGATGRYDKIKDIAFEQAFILNMVGINK 685
TDE_ASLM MNMDSGHGGATGRYDKIKDIAFEQAFILNMVGINK 685
TDE_H-22 MNMDSGHGGATGRYDKIKDIAFEQAFILNMVGINK 685
TDE_H1-T MNMDSGHGGATGRYDKIKDIAFEQAFILNMVGINK 551
TDE_MYR-T MNMDSGHGGATGRYDKIKDIAFEQAFILNMVGINK 673
TDE_OTK MNMDSGHGGATGRYDKIKDIAFEQAFILNMVGINK 685
TDE_SP33 MNMDSGHGGATGRYDRIKDTAFEYAFILNMVGINK 685
TDE_SP37 MNMDSGHGGATGRYDKIKDIAFEQAFILNMVGINK 685
TDE_US-Trep MNMDSGHGGATGRYDKIKDIAFEQAFILNMVGINK 685
TPE_TA4 MNMESGHGGATGRYSRFADIAFDYAFILSML---- 715
TPE_TM1 ----------------------------------- 260
TPE_B683 ----------------------------------- 533
TPE_isoM1111 MNMESGHGGATGRYSRFADIAFDYAFILSML---- 395
TPE_isoE1186 ----------------------------------- 592
TPE_isoM1220 ----------------------------------- 634
TPE_isoM1224 ----------------------------------- 558
