# Supplementary material for: Genome-Wide Relatedness of Treponema pedis, from Gingiva and Necrotic Skin Lesions of Pigs, with the Human Oral Pathogen Treponema denticola
Source: PLoS One. 2013 Aug 19;8(8):e71281. doi: 10.1371/journal.pone.0071281 (PMC3747143; doi:10.1371/journal.pone.0071281)
Supplement: Figure S2 — Amino acid alignments of T. denticola ATCC 35405 PtrB protease homologue (TDE1195) and identified homologues. Described catalytic residues are indicated along with their corresponding positions in TDE1195. (PDF) [file pone.0071281.s002.pdf]

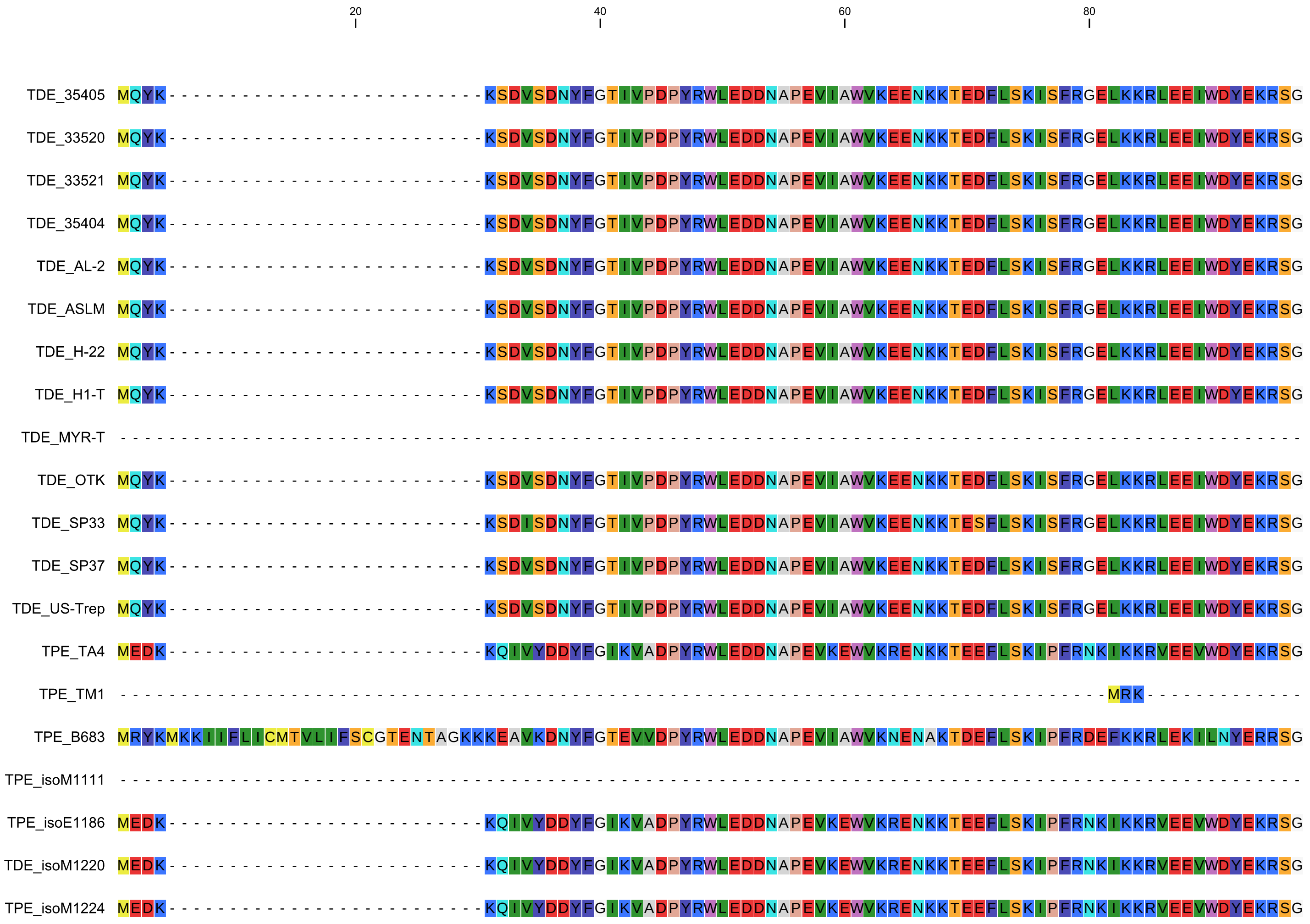
20 40 60 80
TDE_35405 MQYK--------------------------KSDVSDNYFGTIVPDPYRWLEDDNAPEVIAWVKEENKKTEDFLSKISFRGELKKRLEEIWDYEKRSG
TDE_33520 MQYK--------------------------KSDVSDNYFGTIVPDPYRWLEDDNAPEVIAWVKEENKKTEDFLSKISFRGELKKRLEEIWDYEKRSG
TDE_33521 MQYK--------------------------KSDVSDNYFGTIVPDPYRWLEDDNAPEVIAWVKEENKKTEDFLSKISFRGELKKRLEEIWDYEKRSG
TDE_35404 MQYK--------------------------KSDVSDNYFGTIVPDPYRWLEDDNAPEVIAWVKEENKKTEDFLSKISFRGELKKRLEEIWDYEKRSG
TDE_AL-2 MQYK--------------------------KSDVSDNYFGTIVPDPYRWLEDDNAPEVIAWVKEENKKTEDFLSKISFRGELKKRLEEIWDYEKRSG
TDE_ASLM MQYK--------------------------KSDVSDNYFGTIVPDPYRWLEDDNAPEVIAWVKEENKKTEDFLSKISFRGELKKRLEEIWDYEKRSG
TDE_H-22 MQYK--------------------------KSDVSDNYFGTIVPDPYRWLEDDNAPEVIAWVKEENKKTEDFLSKISFRGELKKRLEEIWDYEKRSG
TDE_H1-T MQYK--------------------------KSDVSDNYFGTIVPDPYRWLEDDNAPEVIAWVKEENKKTEDFLSKISFRGELKKRLEEIWDYEKRSG
TDE_MYR-T ---
TDE_OTK MQYK--------------------------KSDVSDNYFGTIVPDPYRWLEDDNAPEVIAWVKEENKKTEDFLSKISFRGELKKRLEEIWDYEKRSG
TDE_SP33 MQYK--------------------------KSDISDNYFGTIVPDPYRWLEDDNAPEVIAWVKEENKKTESFLSKISFRGELKKRLEEIWDYEKRSG
TDE_SP37 MQYK--------------------------KSDVSDNYFGTIVPDPYRWLEDDNAPEVIAWVKEENKKTEDFLSKISFRGELKKRLEEIWDYEKRSG
TDE_US-Trep MQYK--------------------------KSDVSDNYFGTIVPDPYRWLEDDNAPEVIAWVKEENKKTEDFLSKISFRGELKKRLEEIWDYEKRSG
TPE_TA4 MEDK--------------------------KQIVYDDYFGIKVADPYRWLEDDNAPEVKEWVKRENKKTEEFLSKIPFRNKIKKRVEEVWDYEKRSG
TPE_TM1 ---MRK-------------
TPE_B683 MRYKMKKIIFLICMTVLIFSCGTENTAGKKKEAVKDNYFGTEVVDPYRWLEDDNAPEVIAWVKNENAKTDEFLSKIPFRDEFKKRLEKILNYERRSG
TPE_isoM1111 ---
TPE_isoE1186 MEDK--------------------------KQIVYDDYFGIKVADPYRWLEDDNAPEVKEWVKRENKKTEEFLSKIPFRNKIKKRVEEVWDYEKRSG
TDE_isoM1220 MEDK--------------------------KQIVYDDYFGIKVADPYRWLEDDNAPEVKEWVKRENKKTEEFLSKIPFRNKIKKRVEEVWDYEKRSG
TPE_isoM1224 MEDK--------------------------KQIVYDDYFGIKVADPYRWLEDDNAPEVKEWVKRENKKTEEFLSKIPFRNKIKKRVEEVWDYEKRSG

```
                100                 120                 140                 160                 180
TDE_35405     LFKAGNFYYFFRTEGLQNQSIMCRQSGNIKAESSPEVFFDPNKLSADGTTALKNLAFSKDGKYMAYSVSGSGSDWEEIFVFDAEKKADTGEHIHWVKF
TDE_33520     LFKAGNFYYFFRTEGLQNQSIMCRQSGNTKAESSPEVFFDPNKLSSDGTTALKNLAFSKDGKYMAYSVSGSGSDWEEIFVFDAEKKADTGEHIHWVKF
TDE_33521     LFKAGNFYYFFRTEGLQNQSIMCRQSGNIKAESSPEVFFDPNKLSADGTTALKNLAFSKDGKYMAYSVSGSGSDWEEIFVFDAEKKADTGEHIHWVKF
TDE_35404     LFKAGNFYYFFRTEGLQNQSIMCRQSGNIKAESSPEVFFDPNKLSADGTTALKNLAFSKDGKYMAYSVSGSGSDWEEIFVFDAEKKADTGEHIHWVKF
TDE_AL-2      LFKAGNFYYFFRTEGLQNQSIMCRQSGNTKAESSPEVFFDPNKLSSDGTTALKNLAFSKDGKYMAYSVSGSGSDWEEIFVFDAEKKADTGEHIHWVKF
TDE_ASLM      LFKAGNFYYFFRTEGLQNQSIMCRQSGNTKAESSPEVFFDPNKLSSDGTTALKNLAFSKDGKYMAYSVSGSGSDWEEIFVFDAEKKADTGEHIHWVKF
TDE_H-22      LFKAGNFYYFFRTEGLQNQSIMYRQSGNIKAESSPEVFFDPNKLSADGTTALKNLAFSKDGKYMAYSVSGSGSDWEEIFVFDAEKKADTGEHIHWVKF
TDE_H1-T      LFKAGNFYYFFRTEGLQNQSIMCRQSGNTKAESSPEVFFDPNKLSSDGTTALKNLAFSKDGKYMAYSVSGSGSDWEEIFVFDAEKKADTGEHIHWVKF
TDE_MYR-T     --------------------------------------------------------------------------------------------------
TDE_OTK       LFKAGNFYYFFRTEGLQNQSIMCRQSGNTKAESSPEVFFDPNKLSSDGTTALKNLAFSKDGKYMAYSVSGSGSDWEEIFVFDAEKKADTGEHIHWVKF
TDE_SP33      LFKAGNFYYFFRTEGLQNQSIMYRQNGNTKAESSPEVFFDPNKLSSDGTTALKNIAFSKDGKYMAYSVSGSGSDWEEIFVFDAEKKADTGEHIHWVKF
TDE_SP37      LFKAGNFYYFFRTEGLQNQSIMCRQSGNIKAESSPEVFFDPNKLSSDGTTALKNLAFSKDGKYMAYSVSGSGSDWEEIFVFDAEKKADTGEHIHWVKF
TDE_US-Trep   LFKAGNFYYFFRTEGLQNQSIMCRQSGNIKAESSPEVFFDPNKLSSDGTTALKNLAFSKDGKYMAYSVSGSGSDWEEIFVFDAEKKADTGEHIHWVKF
TPE_TA4       LFKAGNYFYFFRTEGLQNQSVMYRQKNSEKAEENPELFFDPNTLSNDGTIALKNLRFSKDGKYMAYSVSGSGSDWEEIFVFDAEKKALTEDNIKWIKF
TPE_TM1       ------------------------------------------------------------------------------------KXALTEDNIKWIKF
TPE_B683      FFKAGEYYYFSKTDGLQNQGIIYRQKNAGNAEEEAEVFFDPNTLSEDGTAALKSMSFSEDGKYMAYSVSKSGSDWEEIFVMDTEKKERLEDRIEWVKF
TPE_isoM1111  --------------------------------------------------------------------------------------------------
TPE_isoE1186  LFKAGNYFYFFRTEGLQNQSVMYRQKNSEKAEENPELFFDPNTLSNDGTIALKNLRFSKDGKYMAYSVSGSGSDWEEIFVFDAEKKALTEDNIKWIKF
TDE_isoM1220  LFKAGNYFYFFRTEGLQNQSVMYRQKNSEKAEENPELFFDPNTLSNDGTIALKNLRFSKDGKYMAYSVSGSGSDWEEIFVFDAEKKALTEDNIKWIKF
TPE_isoM1224  LFKAGNYFYFFRTEGLQNQSVMYRQKNSEKAEENPELFFDPNTLSNDGTIALKNLRFSKDGKYMAYSVSGSGSDWEEIFVFDAEKKALTEDNIKWIKF
```

200 220 240 260 280

```
TDE_35405      SNIAWYKDGFFYSSYDTPDKGKSLTEKNEFQKLKYHKLGTKESDDLLIFEDKDHPLRSFSASTTEDEKTLLLTAFEVGSEGNMLFVADLSEGLPKCSH
TDE_33520      SNIAWYKDGFFYSSYDTPDKGKSLTEKNEFQKLKYHKLGTKESDDLLIFEDKDHPLRSFSASTTEDEKTLLLTAFEVGSEGNMLFVADLSEGLPKCSH
TDE_33521      SNIAWYKDGFFYSSYDTPDKGKSLTEKNEFQKLKYHKLGTKESDDLLIFEDKDHPLRSFSASTTEDEKTLLLTAFEVGSEGNMLFVADLSEGLPKCSH
TDE_35404      SNIAWYKDGFFYSSYDTPDKGKSLTEKNEFQKLKYHKLGTKESDDLLIFEDKDHPLRSFSASTTEDEKTLLLTAFEVGSEGNMLFVADLSEGLPKCSH
TDE_AL-2       SNIAWYKDGFFYSSYDAPDKGKSLTEKNEFQKLKYHKLGTKESDDLLIFEDKDHPLRSFSASTTEDEKTLLLTAFEVGSEGNMLFVADLSEGLPKCSG
TDE_ASLM       SNIAWYKDGFFYSSYDAPDKGKSLTEKNEFQKLKYHKLGTKESDDLLIFEDKEHPLRSFSASTTEDEKTLLLTAFEVGSEGNMLFVADLSEGLPKCSH
TDE_H-22       SNIAWYKDGFFYSSYDAPDKGKSLTEKNEFQKLKYHKLGTKESDDLLIFEDKDHPLRSFSASTTEDEKTLLLTAFEVGSEGNMLFVADLSEGLPKCSH
TDE_H1-T       SNIAWYKDGFFYSSYDAPDKGKSLTEKNEFQKLKYHKLGTKESDDLLIFEDKDHPLRSFSASTTEDEKTLLLTAFEVGSEGNMLFVADLSEGLPKCSH
TDE_MYR-T      ----------------------------------------------------------------------------------MLFVADLSEGLPKCSH
TDE_OTK        SNIAWYKDGFFYSSYDAPDKGKSLTEKNEFQKLKYHKLGTKESDDLLIFEDKEHPLRSFSASTTEDEKTLLLTAFEVGSEGNMLFVADLSEGLPKCSH
TDE_SP33       SNIAWYKDGFFYSSYDAPDKGKSLTEKNEFQKLKYHKLGTKESDDLLIFEDKDHPLRSFSASTTEDEKTLLLTAFEVGNEGNMLFVADLSEGLPKS--
TDE_SP37       SNIAWYKDGFFYSSYDAPDKGKSLTEKNEFQKLKYHKLGTKESDDLLIFEDKDHPLRSFSASTTEDEKTLLLTAFEVGSEGNMLFVADLSEGLPKCSN
TDE_US-Trep    SNIAWYKDGFFYSSYDAPDKGKSLTEKNEFQKLKYHKLGTKESDDLLIFEDKDHPLRSFSASTTEDEKTLLLTAFEVGSEGNMLFVADLSEGLPKCSN
TPE_TA4        SDIAWYKDGFFYSCYDAVGEGKALTEKNEFQKIKYHKLGTPETEDKLIFNDTEHPLRSFTASTSEDEKTLFIYANEAGSEGFLVFIADLQKGLPQSKD
TPE_TM1        SDIAWYKDGFFYSCYDAVGEGKALTEKNEFQKIKYHKLGTPETEDKLIFNDTEHPLRSFTASTSEDEKTLFIYANEAGSEGFLVFIADLQKGLPQSKD
TPE_B683       SDLSWYKDGFFYNCYDAPEGKNALTEKNEFQKVKYHKLGTSTKDDKLIFSDDKNPYKIFSSIAFEPDGILFVIAQALGNEGNSLYIADLKRGFPEAEL
TPE_isoM1111   ----------------------------------------------------------------------------------LVFIADLQKGLPQSKD
TPE_isoE1186   SDIAWYKDGFFYSCYDAVGEGKALTEKNEFQKIKYHKLGTPETEDKLIFNDTEHPLRSFTASTSEDENTLFIYANEAGSEGFLVFIADLQKGLPQSKD
TDE_isoM1220   SDIAWYKDGFFYSCYDAVGEGKALTEKNEFQKIKYHKLGTPETEDKLIFNDTEHPLRSFTASTSEDEKTLFIYANEAGSEGFLVFIADLQKGLPQSKD
TPE_isoM1224   SDIAWYKDGFFYSCYDAVGEGKALTEKNEFQKIKYHKLGTPETEDKLIFNDTEHPLRSFTASTSEDENTLFIYANEAGSEGFLVFIADLQKGLPQSKD
```

```
                300                 320                 340                 360                 380
TDE_35405     CFKQYNTHFNDSVWPLETENGFLYLLTNKQAPFYRVVKTSLNNISEKSIDEVIPQKDCLLSSAALCGGKLLTVYLRDVQDEAFICGLDGKNSTKINLP
TDE_33520     CFKQYNTHFNDSVWPLETESGFLYLLTNKQAPFYRVVKTSLNNISEKSIDEVIPQKDCLLSSAALCGGKLLTVYLRDVQDEAFICGLDGKNSTKINLP
TDE_33521     CFKQYNTHFNDSVWPLETENGFLYLLTNKQAPFYRVVKTSLNNISEKSIDEVIPQKDCLLSSAALCGGKLLTVYLRDVQDEAFICGLDGKNSTKINLP
TDE_35404     CFKQYNTHFNDSVWPLETENGFLYLLTNKQAPFYRVVKTSLNNISEKSIDEVIPQKDCLLSSAALCGGKLLTVYLRDVQDEAFICGLDGKNSTKINLP
TDE_AL-2      CFKQYNTHFNDSVWPLETENGFLYLLTNKQAPFYRVVKTSLNNISEKSIDEVIPQKDCLLSSAALCGGKLLTVYLRDVQDEAFICGLDGKNSTKINLP
TDE_ASLM      CFKQYNTHFNDSVWPLETENGFLYLLTNKQAPFYRVVKTSLNNISEKSIDEVIPQKDCLLSSAALCGGKLLTVYLRDVQDEAFICGLDGKNSTKINLP
TDE_H-22      CFKQYNTHFNDSVWPLETENGFLYLLTNKQAPFYRVVKTSLNNISEKSIDEVIPQKDCLLSSAALCGGKLLTVYLRDVQDEAFICDLDGKNSTKINLP
TDE_H1-T      CFKQYNTHFNDSVWPLETENDFLYLLTNKQAPFYRVVKTSLNNISEKSIDEVIPQKDCLLSSAALCGGKLLTVYLRDVQDEAFICGLDGKNSTKINLP
TDE_MYR-T     CFKQYNTHFNDSVWPLETENDFLYLLTNKQAPFYRVVKTSLNNISEKSIDEVIPQKDCLLSSAALCGGKLLTVYLRDVQDEAFICGLDGKNSTKINLP
TDE_OTK       CFKQYNTHFNDSVWPLETENGFLYLLTNKQAPFYRVVKTSLNNISEKSIDEVIPQKDCLLSSAALCGGKLLTVYLRDVQDEAFICGLDGKNSTKINLP
TDE_SP33      -FKQYNTHFNDNVWPLETDDGFLYLLTNHKSPLYRIVKTPLNAADEKNIEEIIPEQDCLLSSAALCGGKLLTIYLRDVQDEAFICGLDGKNSTKINLP
TDE_SP37      CFKQYNTHFNDSVWPLETENGFLYLLTNKQAPFYRVVKTSLNNISEKSIDEVIPQKDCLLSSAALCGGKLLTVYLRDVQDEAFICGLDGKNGTKINLP
TDE_US-Trep   CFKQYNTHFNDSVWPLETENGFLYLLTNKQAPFYRVVKTSLNNISEKSIDEVIPQKDCLLSSAALCGGKLLTVYLRDVQDEAFICGLDGKNGTKINLP
TPE_TA4       CFVQYNKDFKNTVYPIETDGGYLFLMTNKDAPFYKLVKTPLANPSETNIEDVIPEKKCLLSSCAICGGKILTVYIKDVQDTVYIYGIDGKNEKRVALP
TPE_TM1       CFVQYNKDFKNTVYPIETDGGYLFLMTNKDAPFYKLVKTPLANPSETNIEDVIPEKKCLLSSCAICGGKILTVYIKDVQDTVYIYGIDGKNEKRVALP
TPE_B683      SFKQYNRGAGEAVSPAGISGKYLYLLTNEGTPFYKLVRTSIDNPSAESSEDVLSGQDYLLSSCVVTEDKILALYFKDVQNFAVVYDLNGKNPVEVKLP
TPE_isoM1111  CFVQYNKDFKNTVYPIETDGGYLFLMTNKDAPFYKLVKTPLANPSETNIEDVIPEKKCLLSSCAICGGKILTVYIKDVQDTVYIYGIDGKNEKRVALP
TPE_isoE1186  CFVQYNKDFKNTVYPIETDGGYLFLMTNKDAPFYKLVKTPLANPSETNIEDVIPEKKCLLSSCAICGGKILTVYIKDVQDTVYIYGIDGKNEKRVALP
TDE_isoM1220  CFVQYNKDFKNTVYPIETDGGYLFLMTNKDAPFYKLVKTPLANPSETNIEDVIPEKKCLLSSCAICGGKILTVYIKDVQDTVYIYGIDGKNEKRVALP
TPE_isoM1224  CFVQYNKDFKNTVYPIETDGGYLFLMTNKDAPFYKLVKTPLANPSETNIEDVIPEKKCLLSSCTICGGKILTVYIKDVQDTVYIYGIDGKNEKRVALP
```

```
            400                 420                 440                 460                 480
TDE_35405    ANGSISFSGTRKNEDSLFFNFTSYTTPNKIIRYDIKTNSLTDFFVPAIPINTGDFKCEQVFFKSKDGTKIPMHIVSKKDIKLDGSNPTIMYGYGGFAI
TDE_33520    ANGSISFSGTRKNEDSLFFNFTSYTTPNKIIRYDIKTNSLTDFFVPAIPINTGDFKCEQVFFKSKDGTKIPMHIVSKKDIKLDGSNPTIMYGYGGFAI
TDE_33521    ANGSISFSGTRKNEDSLFFNFTSYTTPNKIIRYDIKTNSLTDFFVPAIPINTGDFKCEQVFFKSKDGTKIPMHIVSKKDIKLDGSNPTIMYGYGGFAI
TDE_35404    ANGSISFSGTRKNEDSLFFNFTSYTTPNKIIRYDIKTNSLTDFFVPAIPINTGDFKCEQVFFKSKDGTKIPMHIVSKKDIKLDGSNPTIMYGYGGFAI
TDE_AL-2     ANGSISFSGTRKNEDSLFFNFTSYTTPNKIIRYDIKTNSLTDFFVPAIPINTGDFKCEQVFFKSKDGTKIPMHIVSKKDIKLDGSNPTIMYGYGGFAI
TDE_ASLM     ANGSISFSGTRKNEDSLFFNFTSYTTPNKIIRYDIKTNSLTDFFVPAIPINTGDFKCEQVFFKSKDGTKIPMHIVSKKDIKLDGSNPTIMYGYGGFAI
TDE_H-22     ANGSISFSGTRKNEDSLFFNFTSYTTPNKIIRYDIKTNSLTDFFVPAIPINTGDFKCEQVFFKSKDGTKIPMHIVSKKDIKLDGSNPTIMYGYGGFAI
TDE_H1-T     ANGSISFSGTRKNEDSLFFNFTSYTTPNKIIRYDIKTNSLTDFFVPAIPINTGDFKCEQVFFKSKDGTKIPMHIVSKKDIKLDGSNPTIMYGYGGFAI
TDE_MYR-T    ANGSISFSGTRKNEDSLFFNFTSYTTPNKIIRYDIKTNSLTDFFVPAIPINTGDFKCEQVFFKSKDGTKIPMHIVSKKDIKLDGSNPTIMYGYGGFAI
TDE_OTK      ANGSISFSGTRKNEDSLFFNFTSYTTPNKIIRYDIKTNSLTDFFVPAIPINTEDFKCEQVFFKSKDGTKIPMHIVSKKDIKLDGNNPTIMYGYGGFAI
TDE_SP33     TNGSISFSGTRKNEGSLFFNFTSYTTPNKIIRYDIKTNSLTDFFVPAIPINTEEFKCEQVFFNSKDGTKIPMHIVSKKDIKLDGNNPTILYGYGGFAI
TDE_SP37     ANGSISFSGTRKNEDSLFFNFTSYTTPNKIIRYDIKTNSLTDFFVPAIPIDTGNFKCEQVFFKSKDGTKIPMHIVSKKDIKLDGNNPTIMYGYGGFAI
TDE_US-Trep  ANGSISFSGTRKNEDSLFFNFTSYTTPNKIIRYDIKTNSLTDFFVPAIPIDTGNFKCEQVFFKSKDGTKIPMHIVSKKDIKLDGNNPTIMYGYGGFAI
TPE_TA4      ENGSITFSGARKNENFLFFAYTSYITPNKIIKYDIEKNELKDFFTPAVDFDDKKYKCEQVFFASKDGTKIPMHIVYKSDIKLDRNNPTIMYGYGGFAI
TPE_TM1      ENGSITFSGARKNENFLFFAYTSYITPNKIIKYDIEKNELKDFFTPAVDFDDKKYKCEQVFFASKDGTKIPMHIVYKSDIKLDGNNPTIMYGYGGFAI
TPE_B683     KNGSISYLGADEEDNTVYLSFTSYTTPGKIVKYDIKKNILTDFFVPSIDFNPNDYEAKQVFFTSKDGTKVPMHIVSKKGIKLDGNNPTLMYGYGGFGA
TPE_isoM1111 ENGSITFSGARKNENFLFFAYTSYITPNKIIKYDIEKNELKDFFTPAVDFDDKKYKCEQVFFASKDGTKIPMHIVYKSDIKLDGNNPTIMYGYGGFAI
TPE_isoE1186 ENGSITFSGARKSENFLFFAYTSYITPNKIIKYDIEKNELKDFFTPAVDFDDKKYKCEQVFFASKDGTKIPMHIVYKSDIKLDGNNPTIMYGYGGFAI
TDE_isoM1220 ENGSITFSGARKNENFLFFAYTSYITPNKIIKYDIEKNELKDFFTPAVDFDDKKYKCEQVFFASKDGTKIPMHIVYKSDIKLDGNNPTIMYGYGGFAI
TPE_isoM1224 ENGSITFSGARKSENFLFFAYTSYITPNKIIKYDIEKNELKDFFTPAVDFDDKKYKCEQVFFASKDGTKIPMHIVYKSDIKLDGNNPTIMYGYGGFAI
```

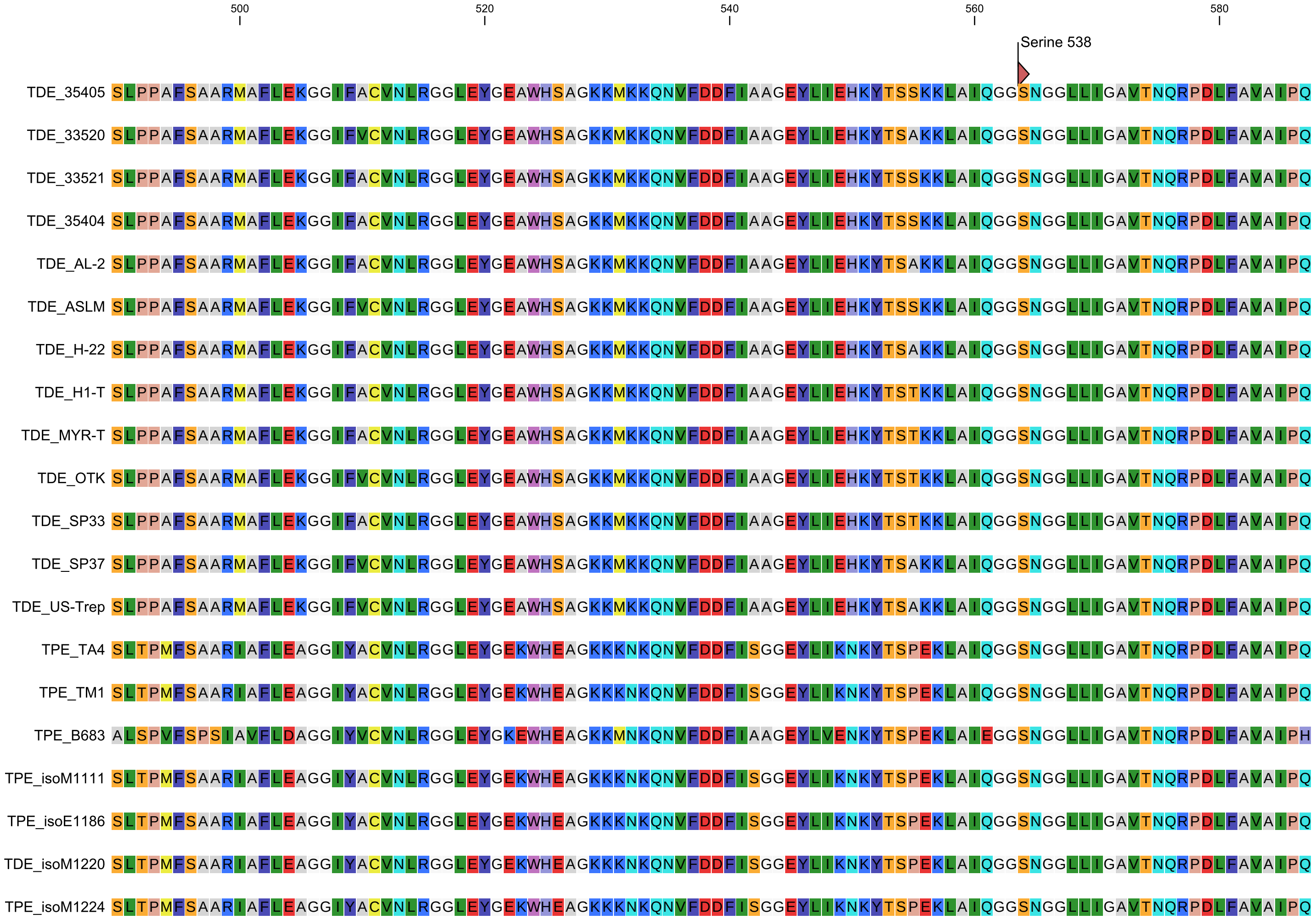
500
520
540
560
580
Serine 538
TDE_35405 SLPPAFSAARMAFLEKGGIFACVNLRGGLEYGEAWHSAGKKMKKQNVFDDFIAAGEYLIEHKYTSSKKLAIQGGSNGGLLIGAVTNQRPDLFAVAIPQ
TDE_33520 SLPPAFSAARMAFLEKGGIFVCVNLRGGLEYGEAWHSAGKKMKKQNVFDDFIAAGEYLIEHKYTSAKKLAIQGGSNGGLLIGAVTNQRPDLFAVAIPQ
TDE_33521 SLPPAFSAARMAFLEKGGIFACVNLRGGLEYGEAWHSAGKKMKKQNVFDDFIAAGEYLIEHKYTSSKKLAIQGGSNGGLLIGAVTNQRPDLFAVAIPQ
TDE_35404 SLPPAFSAARMAFLEKGGIFACVNLRGGLEYGEAWHSAGKKMKKQNVFDDFIAAGEYLIEHKYTSSKKLAIQGGSNGGLLIGAVTNQRPDLFAVAIPQ
TDE_AL-2 SLPPAFSAARMAFLEKGGIFACVNLRGGLEYGEAWHSAGKKMKKQNVFDDFIAAGEYLIEHKYTSAKKLAIQGGSNGGLLIGAVTNQRPDLFAVAIPQ
TDE_ASLM SLPPAFSAARMAFLEKGGIFVCVNLRGGLEYGEAWHSAGKKMKKQNVFDDFIAAGEYLIEHKYTSSKKLAIQGGSNGGLLIGAVTNQRPDLFAVAIPQ
TDE_H-22 SLPPAFSAARMAFLEKGGIFACVNLRGGLEYGEAWHSAGKKMKKQNVFDDFIAAGEYLIEHKYTSAKKLAIQGGSNGGLLIGAVTNQRPDLFAVAIPQ
TDE_H1-T SLPPAFSAARMAFLEKGGIFACVNLRGGLEYGEAWHSAGKKMKKQNVFDDFIAAGEYLIEHKYTSTKKLAIQGGSNGGLLIGAVTNQRPDLFAVAIPQ
TDE_MYR-T SLPPAFSAARMAFLEKGGIFACVNLRGGLEYGEAWHSAGKKMKKQNVFDDFIAAGEYLIEHKYTSTKKLAIQGGSNGGLLIGAVTNQRPDLFAVAIPQ
TDE_OTK SLPPAFSAARMAFLEKGGIFVCVNLRGGLEYGEAWHSAGKKMKKQNVFDDFIAAGEYLIEHKYTSTKKLAIQGGSNGGLLIGAVTNQRPDLFAVAIPQ
TDE_SP33 SLPPAFSAARMAFLEKGGIFACVNLRGGLEYGEAWHSAGKKMKKQNVFDDFIAAGEYLIEHKYTSTKKLAIQGGSNGGLLIGAVTNQRPDLFAVAIPQ
TDE_SP37 SLPPAFSAARMAFLEKGGIFVCVNLRGGLEYGEAWHSAGKKMKKQNVFDDFIAAGEYLIEHKYTSAKKLAIQGGSNGGLLIGAVTNQRPDLFAVAIPQ
TDE_US-Trep SLPPAFSAARMAFLEKGGIFVCVNLRGGLEYGEAWHSAGKKMKKQNVFDDFIAAGEYLIEHKYTSAKKLAIQGGSNGGLLIGAVTNQRPDLFAVAIPQ
TPE_TA4 SLTPMFSAARIAFLEAGGIYACVNLRGGLEYGEKWHEAGKKKNKQNVFDDFISGGEYLIKNKYTSPEKLAIQGGSNGGLLIGAVTNQRPDLFAVAIPQ
TPE_TM1 SLTPMFSAARIAFLEAGGIYACVNLRGGLEYGEKWHEAGKKKNKQNVFDDFISGGEYLIKNKYTSPEKLAIQGGSNGGLLIGAVTNQRPDLFAVAIPQ
TPE_B683 ALSPVFSPSIAVFLDAGGIYVCVNLRGGLEYGKEWHEAGKKMNKQNVFDDFIAAGEYLVENKYTSPEKLAIEGGSNGGLLIGAVTNQRPDLFAVAIPH
TPE_isoM1111 SLTPMFSAARIAFLEAGGIYACVNLRGGLEYGEKWHEAGKKKNKQNVFDDFISGGEYLIKNKYTSPEKLAIQGGSNGGLLIGAVTNQRPDLFAVAIPQ
TPE_isoE1186 SLTPMFSAARIAFLEAGGIYACVNLRGGLEYGEKWHEAGKKKNKQNVFDDFISGGEYLIKNKYTSPEKLAIQGGSNGGLLIGAVTNQRPDLFAVAIPQ
TDE_isoM1220 SLTPMFSAARIAFLEAGGIYACVNLRGGLEYGEKWHEAGKKKNKQNVFDDFISGGEYLIKNKYTSPEKLAIQGGSNGGLLIGAVTNQRPDLFAVAIPQ
TPE_isoM1224 SLTPMFSAARIAFLEAGGIYACVNLRGGLEYGEKWHEAGKKKNKQNVFDDFISGGEYLIKNKYTSPEKLAIQGGSNGGLLIGAVTNQRPDLFAVAIPQ

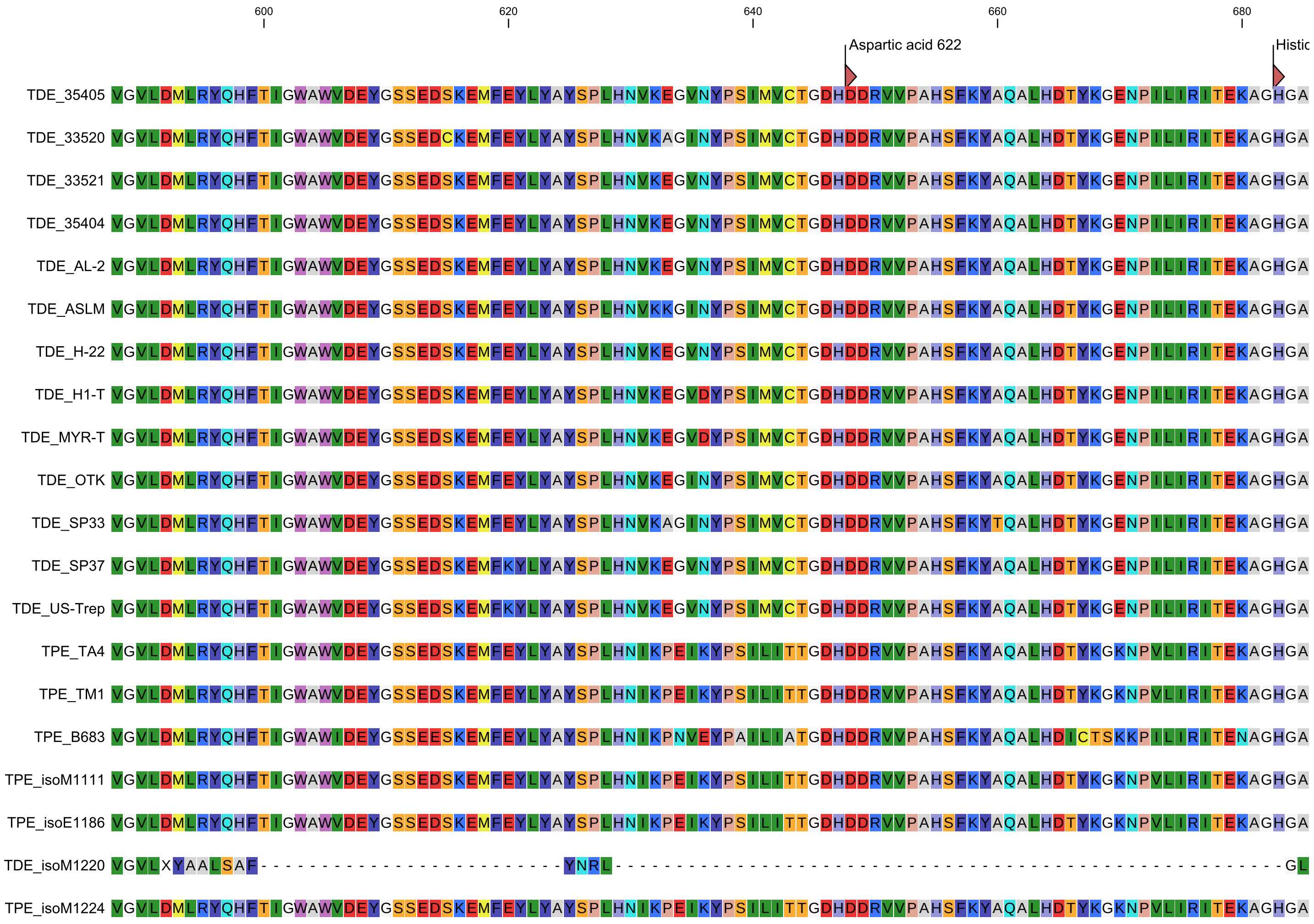
600
620
640
660
680
Aspartic acid 622
Histic
TDE_35405 VGVLDMLRYQHFTIGWAWVDEYGSSEDSKEMFEYLYAYSPLHNVKEGVNYPSIMVCTGDHDDRVVPAHSFKYAQALHDTYKGENPILIRITEKAGHGA
TDE_33520 VGVLDMLRYQHFTIGWAWVDEYGSSEDCKEMFEYLYAYSPLHNVKAGINYPSIMVCTGDHDDRVVPAHSFKYAQALHDTYKGENPILIRITEKAGHGA
TDE_33521 VGVLDMLRYQHFTIGWAWVDEYGSSEDSKEMFEYLYAYSPLHNVKEGVNYPSIMVCTGDHDDRVVPAHSFKYAQALHDTYKGENPILIRITEKAGHGA
TDE_35404 VGVLDMLRYQHFTIGWAWVDEYGSSEDSKEMFEYLYAYSPLHNVKEGVNYPSIMVCTGDHDDRVVPAHSFKYAQALHDTYKGENPILIRITEKAGHGA
TDE_AL-2 VGVLDMLRYQHFTIGWAWVDEYGSSEDSKEMFEYLYAYSPLHNVKEGVNYPSIMVCTGDHDDRVVPAHSFKYAQALHDTYKGENPILIRITEKAGHGA
TDE_ASLM VGVLDMLRYQHFTIGWAWVDEYGSSEDSKEMFEYLYAYSPLHNVKKGINYPSIMVCTGDHDDRVVPAHSFKYAQALHDTYKGENPILIRITEKAGHGA
TDE_H-22 VGVLDMLRYQHFTIGWAWVDEYGSSEDSKEMFEYLYAYSPLHNVKEGVNYPSIMVCTGDHDDRVVPAHSFKYAQALHDTYKGENPILIRITEKAGHGA
TDE_H1-T VGVLDMLRYQHFTIGWAWVDEYGSSEDSKEMFEYLYAYSPLHNVKEGVDYPSIMVCTGDHDDRVVPAHSFKYAQALHDTYKGENPILIRITEKAGHGA
TDE_MYR-T VGVLDMLRYQHFTIGWAWVDEYGSSEDSKEMFEYLYAYSPLHNVKEGVDYPSIMVCTGDHDDRVVPAHSFKYAQALHDTYKGENPILIRITEKAGHGA
TDE_OTK VGVLDMLRYQHFTIGWAWVDEYGSSEDSKEMFEYLYAYSPLHNVKEGINYPSIMVCTGDHDDRVVPAHSFKYAQALHDTYKGENPILIRITEKAGHGA
TDE_SP33 VGVLDMLRYQHFTIGWAWVDEYGSSEDSKEMFEYLYAYSPLHNVKAGINYPSIMVCTGDHDDRVVPAHSFKYTQALHDTYKGENPILIRITEKAGHGA
TDE_SP37 VGVLDMLRYQHFTIGWAWVDEYGSSEDSKEMFKYLYAYSPLHNVKEGVNYPSIMVCTGDHDDRVVPAHSFKYAQALHDTYKGENPILIRITEKAGHGA
TDE_US-Trep VGVLDMLRYQHFTIGWAWVDEYGSSEDSKEMFKYLYAYSPLHNVKEGVNYPSIMVCTGDHDDRVVPAHSFKYAQALHDTYKGENPILIRITEKAGHGA
TPE_TA4 VGVLDMLRYQHFTIGWAWVDEYGSSEDSKEMFEYLYAYSPLHNIKPEIKYPSILITTGDHDDRVVPAHSFKYAQALHDTYKGKNPVLIRITEKAGHGA
TPE_TM1 VGVLDMLRYQHFTIGWAWVDEYGSSEDSKEMFEYLYAYSPLHNIKPEIKYPSILITTGDHDDRVVPAHSFKYAQALHDTYKGKNPVLIRITEKAGHGA
TPE_B683 VGVLDMLRYQHFTIGWAWIDEYGSSEESKEMFEYLYAYSPLHNIKPNVEYPAILIATGDHDDRVVPAHSFKYAQALHDICTSKKPILIRITENAGHGA
TPE_isoM1111 VGVLDMLRYQHFTIGWAWVDEYGSSEDSKEMFEYLYAYSPLHNIKPEIKYPSILITTGDHDDRVVPAHSFKYAQALHDTYKGKNPVLIRITEKAGHGA
TPE_isoE1186 VGVLDMLRYQHFTIGWAWVDEYGSSEDSKEMFEYLYAYSPLHNIKPEIKYPSILITTGDHDDRVVPAHSFKYAQALHDTYKGKNPVLIRITEKAGHGA
TDE_isoM1220 VGVLXYAALSAF - - - - YNRL - - - - GL
TPE_isoM1224 VGVLDMLRYQHFTIGWAWVDEYGSSEDSKEMFEYLYAYSPLHNIKPEIKYPSILITTGDHDDRVVPAHSFKYAQALHDTYKGKNPVLIRITEKAGHGA

```
                        700                 720
                        |                   |
line 657

TDE_35405     GKPTAKIIEETADIYAFIFK------QTGHII-------------------- 685
TDE_33520     GKPTAKIIEETADIYAFIFK------QTGHII-------------------- 685
TDE_33521     GKPTAKIIEETADIYAFIFK------QTGHII-------------------- 685
TDE_35404     GKPTAKIIEETADIYAFIFK------QTGHII-------------------- 685
TDE_AL-2      GKPTAKIIEEMADIYAFIFK------QTGHII-------------------- 685
TDE_ASLM      GKPTAKIIEETADIYAFIFK------QTGHII-------------------- 685
TDE_H-22      GKPTAKIIEETADIYAFIFK------QTGHKI-------------------- 685
TDE_H1-T      GKPTAKIIEETADIYAFIFK------QTGHII-------------------- 685
TDE_MYR-T     GKPTAKIIEETADIYAFIFK------QTGHII-------------------- 434
TDE_OTK       GKPTAKIIEETADIYAFIFK------QTGHII-------------------- 685
TDE_SP33      GKPTAKIIEETADIYAFIFE------QTGHKI-------------------- 682
TDE_SP37      GKPTAKIIEETADIYAFIFK------QTGHKL-------------------- 685
TDE_US-Trep   GKPTAKIIEETADIYAFIFK------QTGHKL-------------------- 685
TPE_TA4       GKPTAKIIEETADIFSFVF------YQTGTDI------KG------------ 687
TPE_TM1       GKPTAKIIEETADIFXVCFLSNRNRYKRLIDIFKNGNFKGSRYQYKKKLLQY 559
TPE_B683      GKPTAKIIEETADIYSFIF------YQMGETL-------------------- 711
TPE_isoM1111  GKPTAKIIEETADIFSFVF------YQTGTDI------KG------------ 436
TPE_isoE1186  GKPTAKIIEETADIFSFVF------YQTGTDI------KG------------ 687
TDE_isoM1220  GR-------------------------------------------------- 581
TPE_isoM1224  GKPTAKIIEETADIFSFVF------YQTGTDI------KG------------ 687
```
