# Supplementary material for: Genome-Wide Relatedness of Treponema pedis, from Gingiva and Necrotic Skin Lesions of Pigs, with the Human Oral Pathogen Treponema denticola
Source: PLoS One. 2013 Aug 19;8(8):e71281. doi: 10.1371/journal.pone.0071281 (PMC3747143; doi:10.1371/journal.pone.0071281)
Supplement: Figure S3 — Amino acid alignments of T. denticola ATCC 35405 protease Dentipain (TDE0362) and identified homologues. Described catalytic residues are indicated along with their corresponding positions in TDE0362. (PDF) [file pone.0071281.s003.pdf]

```
                                      20                  40                  60                  80
TDE_35405      --------------MIKRQKLIGFAAIAAFFLLLSCKNNLLTKTEKEKSGEIPVKNIILSP-NKSEFSLEKNTAQTITVKIIPEKATNKALIYSSKHG
TDE_33520      MQNNVFFRFIGDYFMIKKHKLIGFAAVAAFFLLLSCKNNLLTKTEKEKSGEIPVKNIILSP-NKSEFSLEKNTAQTITVKIIPEKATNKALIYSSKHG
TDE_33521      --------------MIKRQKLIGFAAIAAFFLLLSCKNNLLTKTEKEKSGEIPVKNIILSP-NKSEFSLEKNTAQTITVKIIPEKATNKALIYSSKHG
TDE_35404      --------------MIKRQKLIGFAAIAAFFLLLSCKNNLLTKTEKEKSGEIPVKNIILSP-NKSEFSLEKNTAQTITVKIIPEKATNKALIYSSKHG
TDE_AL-2       --------------------------LQXFFLLLSCKNNLLTKTEKEKSGEIPVKNIILSP-NKSEFSLEKNTAQTITVKIIPEKATNKTLIYSSKHG
TDE_ASLM       MQNNVFFRFIGDYFMIKKLKLIGFAAVAAFFLLLSCKNNLLTKTEKEKSGEIPVKNIILSP-NKSEFSLEKNTAQTITVKIIPEKATNKALIYSSKHG
TDE_H-22       --------------MIKKHKLIGFAAVAAFFLLLSCKNNLLTKAEKEKSGEIPVKNIILSP-NKSEFSLEKNTAQTITVKIIPEKATNKTLIYSSKHG
TDE_H1-T       MQNNVFFRFIGDYFMIKKHKLIGFAAVAAFFLLLSCKNNLLTKTEKEKSGEIPVKNIILSP-NKSEFSLEKNTAQTITVKIIPEKATNKALIYSSKHG
TDE_MYR-T      MQNNVFFRFIGDYFMIKKHKLIGFAAVAAFFLLLSCKNNLLTKTEKEKSGEIPVKNIILSP-NKSEFSLEKNTAQTITVKIIPEKATNKALIYSSKHG
TDE_OTK        MQNNVFFRFIGDYFMIKKHKLIGFAAVAAFFLLLSCKNNLLTKTEKEKSGEIPVKNIILSP-NKSEFSLEKNTAQTITVKIIPEKATNKTLIYSSKHG
TDE_SP33       MQNNVFYKHIGDYFMIKRQKLIGFAAIAAFFLLLSCKNNL--------SPEIPVEDIIISPHNEGEISIVSGMTKNITVKIIPENATNKTLAYSSTPG
TDE_SP37       --------------------------LQXFFLLLSCKNNILTKSEKENSGEIPIKNIILSP-NKNELSLEKNTAQTITVKIIPEKATNKALIYSSKHG
TDE_US-Trep    --------------------------------------------------------------------------------------------------
TPE_TA4        --------------LIKKIIFIAFIA-ASFFMFAACPDNIKFKPKKESAVQA----------DESEQSVKSEVSGT----------------------
TPE_TM1        --------------LIKKIIFIAFIA-ASFFMFAACPDNIKFKPKKESAVQA----------DESEQSVKSEVSGT----------------------
TPE_B683       --------------LIKKIIFIAFIA-ASFFMFAACPDNIKFKPKKESVVQA----------AESAQSAKSELSGT----------------------
TPE_isoM1111   --------------------------------------------------------------------------------------------------
TPE_isoE1186   --------------LIKKIIFIAFIA-ASFFMFAACPDNIKFKPKKESVVQA----------DESAQSAKSELSGT----------------------
TPE_isoM1220   --------------LIKKIIFIAFIA-ASFFMFAACPDNIKFKPKKESAVQA----------DESEQSVKSEVSGT----------------------
TPE_isoM1224   -------------------------------MFAACPDNIKFKPKKESAVQA----------DESEQSVKSELSGT----------------------
```

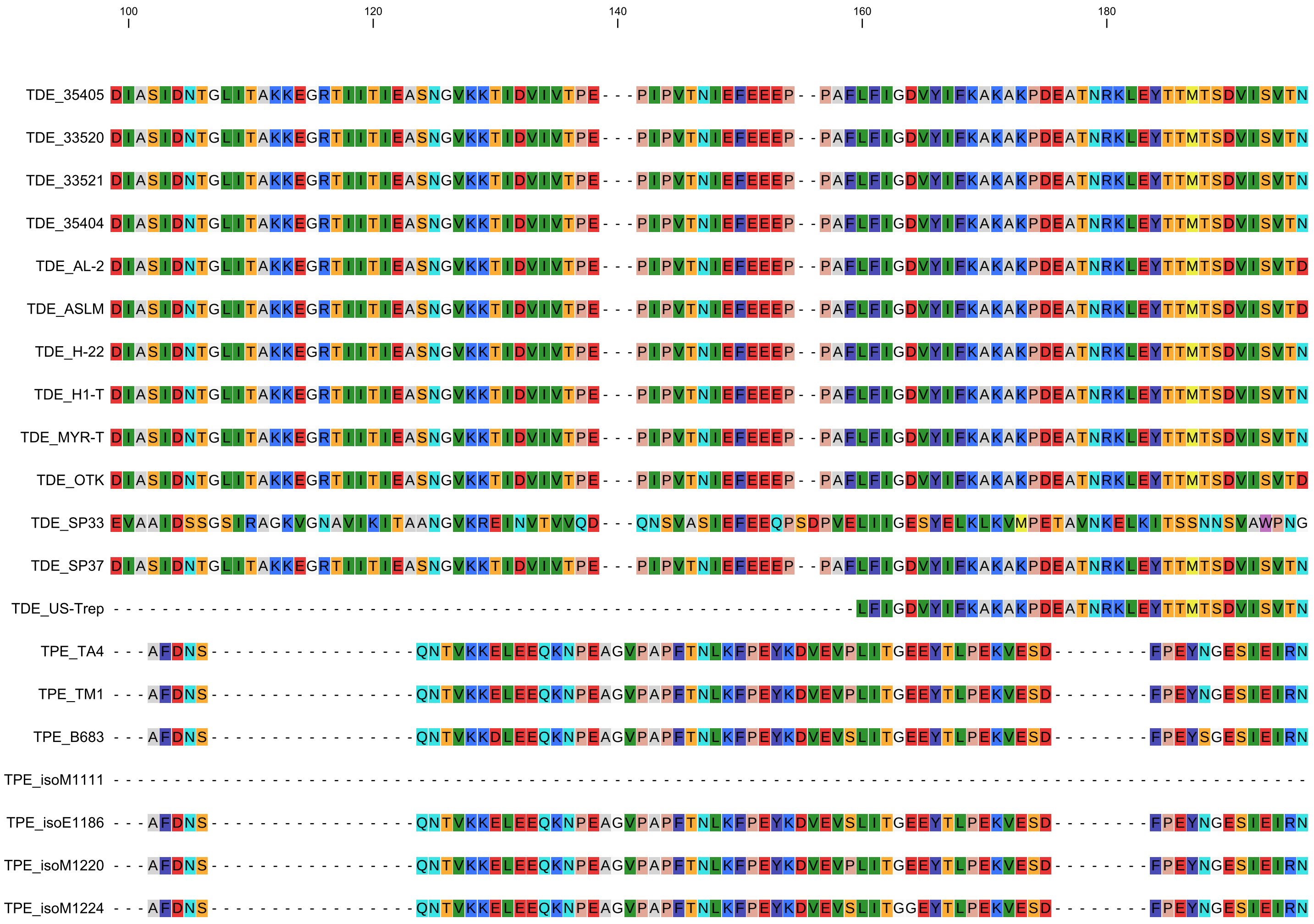
100
120
140
160
180
TDE_35405 DIASIDNTGLITAKKEGRTIITIEASNGVKKTIDVIVTPE---PIPVTNIEFEEEP--PAFLFIGDVYIFKAKAKPDEATNRKLEYTTMTSDVISVTN
TDE_33520 DIASIDNTGLITAKKEGRTIITIEASNGVKKTIDVIVTPE---PIPVTNIEFEEEP--PAFLFIGDVYIFKAKAKPDEATNRKLEYTTMTSDVISVTN
TDE_33521 DIASIDNTGLITAKKEGRTIITIEASNGVKKTIDVIVTPE---PIPVTNIEFEEEP--PAFLFIGDVYIFKAKAKPDEATNRKLEYTTMTSDVISVTN
TDE_35404 DIASIDNTGLITAKKEGRTIITIEASNGVKKTIDVIVTPE---PIPVTNIEFEEEP--PAFLFIGDVYIFKAKAKPDEATNRKLEYTTMTSDVISVTN
TDE_AL-2 DIASIDNTGLITAKKEGRTIITIEASNGVKKTIDVIVTPE---PIPVTNIEFEEEP--PAFLFIGDVYIFKAKAKPDEATNRKLEYTTMTSDVISVTD
TDE_ASLM DIASIDNTGLITAKKEGRTIITIEASNGVKKTIDVIVTPE---PIPVTNIEFEEEP--PAFLFIGDVYIFKAKAKPDEATNRKLEYTTMTSDVISVTD
TDE_H-22 DIASIDNTGLITAKKEGRTIITIEASNGVKKTIDVIVTPE---PIPVTNIEFEEEP--PAFLFIGDVYIFKAKAKPDEATNRKLEYTTMTSDVISVTN
TDE_H1-T DIASIDNTGLITAKKEGRTIITIEASNGVKKTIDVIVTPE---PIPVTNIEFEEEP--PAFLFIGDVYIFKAKAKPDEATNRKLEYTTMTSDVISVTN
TDE_MYR-T DIASIDNTGLITAKKEGRTIITIEASNGVKKTIDVIVTPE---PIPVTNIEFEEEP--PAFLFIGDVYIFKAKAKPDEATNRKLEYTTMTSDVISVTN
TDE_OTK DIASIDNTGLITAKKEGRTIITIEASNGVKKTIDVIVTPE---PIPVTNIEFEEEP--PAFLFIGDVYIFKAKAKPDEATNRKLEYTTMTSDVISVTD
TDE_SP33 EVAAIDSSGSIRAGKVGNAVIKITAANGVKREINVTVVQD---QNSVASIEFEEQPSDPVELIIGESYELKLKVMPETAVNKELKITSSNNSVAWPNG
TDE_SP37 DIASIDNTGLITAKKEGRTIITIEASNGVKKTIDVIVTPE---PIPVTNIEFEEEP--PAFLFIGDVYIFKAKAKPDEATNRKLEYTTMTSDVISVTN
TDE_US-Trep ---LFIGDVYIFKAKAKPDEATNRKLEYTTMTSDVISVTN
TPE_TA4 ---AFDNS-----------------QNTVKKELEEQKNPEAGVPAPFTNLKFPEYKDVEVPLITGEEYTLPEKVESD--------FPEYNGESIEIRN
TPE_TM1 ---AFDNS-----------------QNTVKKELEEQKNPEAGVPAPFTNLKFPEYKDVEVPLITGEEYTLPEKVESD--------FPEYNGESIEIRN
TPE_B683 ---AFDNS-----------------QNTVKKDLEEQKNPEAGVPAPFTNLKFPEYKDVEVSLITGEEYTLPEKVESD--------FPEYSGESIEIRN
TPE_isoM1111 --
TPE_isoE1186 ---AFDNS-----------------QNTVKKELEEQKNPEAGVPAPFTNLKFPEYKDVEVSLITGEEYTLPEKVESD--------FPEYNGESIEIRN
TPE_isoM1220 ---AFDNS-----------------QNTVKKELEEQKNPEAGVPAPFTNLKFPEYKDVEVPLITGEEYTLPEKVESD--------FPEYNGESIEIRN
TPE_isoM1224 ---AFDNS-----------------QNTVKKELEEQKNPEAGVPAPFTNLKFPEYKDVEVSLITGGEYTLPEKVESD--------FPEYNGESIEIRN

```
                  200                 220                 240                 260                 280
TDE_35405    TELGTMKATKE-GNAAITIRSASTPS-VAKTVTIEIKKKP---QIKYEEKQAVMPESAAGTYTFEVQTIDGKLDYEPYFT---SSTLP-WITGAPTIS
TDE_33520    TELGTMKATKE-GNAAITIRSASTPS-VAKTVTIEIKKKP---QIKYEEKQAVMPESAAGTYTFEVQTIDGKLDYEPYFT---SSTLP-WITGAPTIS
TDE_33521    TELGTMKATKE-GNAAITIRSASTPS-VAKTVTIEIKKKP---QIKYEEKQAVMPESAAGTYTFEVQTIDGKLDYEPYFT---SSTLP-WITGAPTIS
TDE_35404    TELGTMKATKE-GNAAITIRSASTPS-VAKTVTIEIKKKP---QIKYEEKQAVMPESAAGTYTFEVQTIDGKLDYEPYFT---SSTLP-WITGAPTIS
TDE_AL-2     TEFGTIKATKE-GNAAITIRSASTPS-VAKTVTIEIKKKP---EIRYEGKTAVMPESAAGTYTFEVQTIGGKLDYEPYFT---SSTLP-WITGAPTIS
TDE_ASLM     TELGTMKATKE-GNAAITIRSASTPS-VAKTVTIEIKKKP---QIKYEEKQAVMPESAAGTYTFEVQTIDGKLDYEPYFT---SSTLP-WITGAPTIS
TDE_H-22     TELGTMKATKE-GNAAITIRSASTPS-VAKTVTIEIKKKP---EIRYEGKTAVMPESAAGTYTFEVQTIGGKLDYEPRFT---SSTLP-WVSGSPAIS
TDE_H1-T     TELGTMKATKE-GNAAITIRSASTPS-VAKTVTIEIKKKP---QIKYEEKQAVMPESAAGTYTFEVQTIDGKLDYEPYFT---SSTLP-WITGAPTIS
TDE_MYR-T    TELGTMKATKE-GNAAITIRSASTPS-VAKTVTIEIKKKP---QIKYEEKQAVMPESAAGTYTFEVQTIDGKLDYEPYFT---SSTLP-WITGAPTIS
TDE_OTK      TEFGTIKATKE-GNAAITIRSASTPS-VAKTVTIEIKKKP---QIKYEEKQAVMPESAAGTYTFEVQTIDGKLDYEPYFT---SSTLP-WITGAPTIS
TDE_SP33     DGNRWIKADGTVGEATVTIISADNSS-IKKEVRFKTKEKPTGPSISIETLD-VEYESDEANITVAVKTLDGKLAYTPEVVGGGTGEKA-WLTFVNKVN
TDE_SP37     TELGTMKATKE-GNAAITIRSASTPS-VAKTVTIEIKKKP---QIKYEEKQAVMPESAAGTYTFEVQTIDGKLDYEPYFT---SSTLP-WITGAPTIS
TDE_US-Trep  TELGTMKATKE-GNAAITIRSASTPS-VAKTVTIEIKKKP---QIKYEEKQAVMPESAAGTYTFEVQTIDGKLDYEPYFT---SSTLP-WITGAPTIS
TPE_TA4      RTVFAINP----GRSVLKVKIKDIPNPVNFTFNITADSKITVDRAEIKITDRIRNENSPSEYTVTVTKQSKHTQYK-------LGPLPEWIKCE---S
TPE_TM1      RTVFAINP----GRSVLKVKIKDIPNPVNFTFNITADSKITVDRAEIKITDRIRNENSPSEYTVTVTKQSKHTQYK-------LGPLPEWIKCE---S
TPE_B683     RTVFAINP----GRSILKVKIKDIPNPVNFTFNITADSKITVDRKEIKITDRIRNENSPSEYTVTVTKQSKHTQYK-------LGSLPEWIKCE---S
TPE_isoM1111 -----------------------------------------------------------------------------------LGSLPEWIKCE---S
TPE_isoE1186 RTVFAINP----GRSILKVKIKDIPNPVNFTFNITADSKITVDRAEIKITDRIRNENSPSEYTVTVTKQSKHTQYK-------LGSLPEWIKCE---S
TPE_isoM1220 RTVFAINP----GRSVLKVKIKDIPNPVNFTFNITADSKITVDRAEIKITDRIRNENSPSEYTVTVTKQSKHTQYK-------LGPLPEWIKCE---S
TPE_isoM1224 RTVFAINP----GRSVLKVKIKDIPNPVNFTFNITADSKITVDRKEIKITDRIRNENSPSEYTVTVTKQSKHTQYK-------LGSLPEWIKCE---S
```

```
               300                 320                 340                 360                 380
               |                   |                   |                   |                   |
TDE_35405    SRTDPNKDVISFTCLKNKTVWNRRAYIKFKDKKTGQYIKGADGKADLTVNIIQKKNENPVVHYKWVDGIGAPTENQKIKMKIKNNGIETEDYFTDPFV
TDE_33520    SRTDPNKDVISFTCLKNKTVWNRRAYIKFKDKKTGQYIKGADGKADLTVNIIQKKNENPVVHYKWVDGIGAPTENQKIKMKIKNNGIETEDYFTDPFV
TDE_33521    SRTDPNKDVISFTCLKNKTVWNRRAYIKFKDKKTGQYIKGADGKADLTVNIIQKKNENPVVHYKWVDGIGAPTENQKIKMKIKNNGIETEDYFTDPFV
TDE_35404    SRTDPNKDVISFTCLKNKTVWNRRAYIKFKDKKTGQYIKGADGKADLTVNIIQKKNENPVVHYKWVDGIGAPTENQKIKMKIKNNGIETEDYFTDPFV
TDE_AL-2     SRTDPNKDVISFTCLKNKTVWNRRAYIKFKDKKTGQYIKGADGKADLIVNIIQKKNENPVVHYKWVDGIGAPTENQKIKMKIKNNGIETEDYFTDPFV
TDE_ASLM     SRTDPNKDVISFTCLKNKTVWNRRAYIKFKDKKTGQYIKGADGKTDLTVNIIQKKNENPVVHYKWVDGIGSPAENEKEKVGINNNGIPTGDTYPQSYV
TDE_H-22     SRTDPNKDVISFTCLKNKTVWDRRAYIKFWDKKTNSYVKNADGKTDLTVNIIQKKNEHPVVHYKWVDGIGAPTENQKIKMKIKNNGIETEDYFTDPFV
TDE_H1-T     SRTDPNKDVISFTCLKNKTVWNRRAYIKFKDKKTGQYIKGADGKADLTVNIIQKKNENPVVHYKWVDGIGAPTENQKIKMKIKNNGIETEDYFTDPFV
TDE_MYR-T    SRTDPNKDVISFTCLKNKTVWNRRAYIKFKDKKTGQYIKGADGKADLTVNIIQKKNENPVVHYKWVDGIGAPTENQKIKMKIKNNGIETEDYFTDPFV
TDE_OTK      SRTDPNKDVISFTCLKNKTVWNRRAYIKFKDKKTGQYIKGADGKADLTVNIIQKKNENPVVHYKWVDGIGAPTENQKIKMKIKNNGIETEDYFTDPFV
TDE_SP33     --TDANTDTVHLHLKKNKTVWDRTAYIKFKDNNTNEYIISA-GKP-LQVELTQKKNEHPIVTIKWVDGIGEPTQTEKKKIPVY--GGEKKVYWDDDKI
TDE_SP37     SRTDPNKDVISFTCLKNKTVWNRRAYIKFKDKKTGQYIKGADGKADLTVNIIQKKNENPVVHYKWVDGIGAPTEDQKVRMKIKNNGIETEDYFTDPFV
TDE_US-Trep  SRTDPNKDVISFTCLKNKTVWNRRAYIKFKDKKTGQYIKGADGKADLTVNIIQKKNENPVVHYKWVDGIGAPTEDQKVRMKIKNNGIETEDYFTDPFV
TPE_TA4      SVTEDLKDKYKF-ILSNDSFFDRDAKIVFTN---------TEGKTLSSISVRQDGNYAVKRNYKWVKGITPPANAE---LKKEYND-R-------PAF
TPE_TM1      SVTEDLKDKYKF-ILSNDSFFDRDAKIVFTN---------TEGKTLSSISVRQDGNYAVKRNYKWVKGITPPANAE---LKKEYND-R-------PAF
TPE_B683     SVTEDLKDKYKF-ILSNDSFFDRNAKIVFTN---------IEGKTLSTISVRQDGNYAVKRNYKWVKGITPPANAE---LKKEYND-R-------PAF
TPE_isoM1111 SVTEDLKDKYKF-ILSNDSFFDRNAKIVFTN---------IEGKTLSTISVRQDGNYNLKRTYQWVKGVKAPMESE---LKKNNPSIS-------PLL
TPE_isoE1186 SVTEDLKDKYKF-ILSNDSFFDRNAEIVFTN---------TEGKTLSTISVRQDGNYNLKRTYQWVKGVKAPMESE---LKKNNPSIS-------PX-
TPE_isoM1220 SVTEDLKDKYKF-ILSNDSFFDRDAKIVFTN---------TEGKTLSSISVRQDGNYAVKRNYKWVKGITPPANAE---LKKEYND-R-------PAF
TPE_isoM1224 SVTEDLKDKYKF-ILSNDSFFDRNAEIVFTN---------TEGKTLSTISVRQDGNYNLKRTYQWVKGVKAPMESE---LKKNNPSIS-------PLL
```

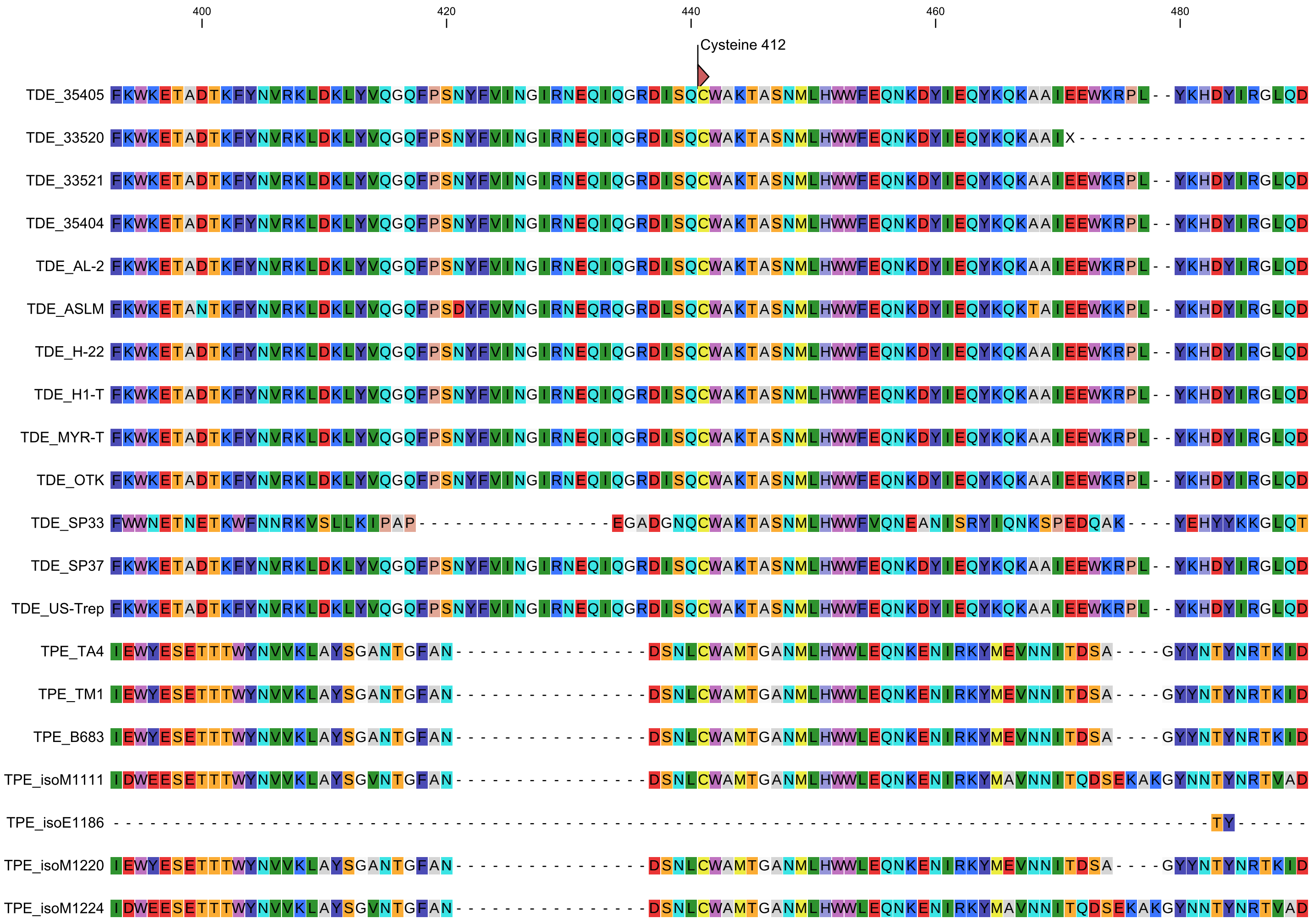
400
420
440
460
480
Cysteine 412
TDE_35405 FKWKETADTKFYNVRKLDKLYVQGQFPSNYFVINGIRNEQIQGRDISQCWAKTASNMLHWWFEQNKDYIEQYKQKAAIEEWKRPL--YKHDYIRGLQD
TDE_33520 FKWKETADTKFYNVRKLDKLYVQGQFPSNYFVINGIRNEQIQGRDISQCWAKTASNMLHWWFEQNKDYIEQYKQKAAIX-------------------
TDE_33521 FKWKETADTKFYNVRKLDKLYVQGQFPSNYFVINGIRNEQIQGRDISQCWAKTASNMLHWWFEQNKDYIEQYKQKAAIEEWKRPL--YKHDYIRGLQD
TDE_35404 FKWKETADTKFYNVRKLDKLYVQGQFPSNYFVINGIRNEQIQGRDISQCWAKTASNMLHWWFEQNKDYIEQYKQKAAIEEWKRPL--YKHDYIRGLQD
TDE_AL-2 FKWKETADTKFYNVRKLDKLYVQGQFPSNYFVINGIRNEQIQGRDISQCWAKTASNMLHWWFEQNKDYIEQYKQKAAIEEWKRPL--YKHDYIRGLQD
TDE_ASLM FKWKETANTKFYNVRKLDKLYVQGQFPSDYFVVNGIRNEQRQGRDLSQCWAKTASNMLHWWFEQNKDYIEQYKQKTAIEEWKKPL--YKHDYIRGLQD
TDE_H-22 FKWKETADTKFYNVRKLDKLYVQGQFPSNYFVINGIRNEQIQGRDISQCWAKTASNMLHWWFEQNKDYIEQYKQKAAIEEWKRPL--YKHDYIRGLQD
TDE_H1-T FKWKETADTKFYNVRKLDKLYVQGQFPSNYFVINGIRNEQIQGRDISQCWAKTASNMLHWWFEQNKDYIEQYKQKAAIEEWKRPL--YKHDYIRGLQD
TDE_MYR-T FKWKETADTKFYNVRKLDKLYVQGQFPSNYFVINGIRNEQIQGRDISQCWAKTASNMLHWWFEQNKDYIEQYKQKAAIEEWKRPL--YKHDYIRGLQD
TDE_OTK FKWKETADTKFYNVRKLDKLYVQGQFPSNYFVINGIRNEQIQGRDISQCWAKTASNMLHWWFEQNKDYIEQYKQKAAIEEWKRPL--YKHDYIRGLQD
TDE_SP33 FWWNETNETKWFNNRKVSLLKIPAP----------------EGADGNQCWAKTASNMLHWWFVQNEANISRYIQNKSPEDQAK----YEHYYKKGLQT
TDE_SP37 FKWKETADTKFYNVRKLDKLYVQGQFPSNYFVINGIRNEQIQGRDISQCWAKTASNMLHWWFEQNKDYIEQYKQKAAIEEWKRPL--YKHDYIRGLQD
TDE_US-Trep FKWKETADTKFYNVRKLDKLYVQGQFPSNYFVINGIRNEQIQGRDISQCWAKTASNMLHWWFEQNKDYIEQYKQKAAIEEWKRPL--YKHDYIRGLQD
TPE_TA4 IEWYESETTTWYNVVKLAYSGANTGFAN----------------DSNLCWAMTGANMLHWWLEQNKENIRKYMEVNNITDSA----GYYNTYNRTKID
TPE_TM1 IEWYESETTTWYNVVKLAYSGANTGFAN----------------DSNLCWAMTGANMLHWWLEQNKENIRKYMEVNNITDSA----GYYNTYNRTKID
TPE_B683 IEWYESETTTWYNVVKLAYSGANTGFAN----------------DSNLCWAMTGANMLHWWLEQNKENIRKYMEVNNITDSA----GYYNTYNRTKID
TPE_isoM1111 IDWEESETTTWYNVVKLAYSGVNTGFAN----------------DSNLCWAMTGANMLHWWLEQNKENIRKYMAVNNITQDSEKAKGYNNTYNRTVAD
TPE_isoE1186 --TY------
TPE_isoM1220 IEWYESETTTWYNVVKLAYSGANTGFAN----------------DSNLCWAMTGANMLHWWLEQNKENIRKYMEVNNITDSA----GYYNTYNRTKID
TPE_isoM1224 IDWEESETTTWYNVVKLAYSGVNTGFAN----------------DSNLCWAMTGANMLHWWLEQNKENIRKYMAVNNITQDSEKAKGYNNTYNRTVAD

```
                       500                 520                 540                 560                 580
                         |                   |                   |                   |                   |
TDE_35405      EDEGKKSNIANIFRAYSHNNARGGYIEDGLTWYLYKRDG-------QKNLGSIYPGLFNDVF--AHDTSPINIER-------CETKKEFEQLMNKTLD
TDE_33520      --------------------------------------------------------------------------R-------VETA------------
TDE_33521      EDEGKKSNIANIFRAYSHNNARGGYIEDGLTWYLYKRDG-------QKNLGSIYPGLFNDVF--AHDTSPINIER-------CETKKEFEQLMNKTLD
TDE_35404      EDEGKKSNIANIFRAYSHNNARGGYIEDGLTWYLYKRDG-------QKNLGSIYPGLFNDVF--AHDTSPINIER-------CETKKEFEQLMNKTLD
TDE_AL-2       EDEGKKSNIANIFRAYSHNNARGGYIEDGLTWYLYKRDG-------QKNLGSIYPGLFNDVF--THDTSPINIER-------CETKKEFEQLMNKALD
TDE_ASLM       KEENKKSNIANIFRAYTHNNSRGGYIEDGLTWYLYKRDA-------QKNLGSIYPGLFNDVF--THDTSPITIER-------CETKKEFEQLMNKALD
TDE_H-22       EDEGKKSNIANIFRAYSHNNARGGYIEDGLTWYLYKRDG-------QKNLGSIYPGLFNDVF--THDTSPINIER-------CETKKEFEQLMNKALD
TDE_H1-T       EDEGKKSNIANIFRAYSHNNARGGYIEDGLTWYLYKRDG-------QKNLGSIYPGLFNDVF--THDTSPINIER-------CETKKEFEQLMNKALD
TDE_MYR-T      EDEGKKSNIANIFRAYSHNNARGGYIEDGLTWYLYKRDG-------QKNLGSIYPGLFNDVF--THDTSPINIER-------CETKKEFEQLMNKALD
TDE_OTK        EDEGKKSNIANIFRAYSHNNARGGYIEDGLTWYLYKRDA-------QKNLGSIYPGLFNDVF--THDTSPINIER-------CETKKEFEQLMNKALD
TDE_SP33       KQEKEKSYIANAFRTKAHNGQLGDYIISGLAWYLYGHPSVTLPTIKEKDSAFEGPALFKDIFDKSENKTPIKVET-------VDGKDSFNKIISNALE
TDE_SP37       EDEGKKSNIANIFRAYSHNNARGGYIEDGLTWYLYKRDG-------QKNLGSIYPGLFNDVF--THDTSPINIER-------CETKKEFEQLMNKALD
TDE_US-Trep    EDEGKKSNIANIFRAYSHNNARGGYIEDGLTWYLYKRDG-------QKNLGSIYPGLFNDVF--THDTSPINIER-------CETKKEFEQLMNKALD
TPE_TA4        SD---KSDIAQAARSCINFRAAGGDVRTLFNWYISGKN---LPAGQRPNGYKDAPGYFRDIFP---DTTPIVNEQYV------NSKEQLESVIKEAFD
TPE_TM1        SD---KSDIAQAARSCINFRAAGGDVRTLFNWYISGKN---LPAGQRPNGYKDAPGYFRDIFP---DTTPIVNEQYV------NSKEQLESVIKEAFD
TPE_B683       SD---KSDIAQAARSCINFRAAGGDVRTLFNWYISGKN---LPTGQRPNGYKDAPGYFRDIFP---DTTPIVNEQYV------NSKEQLESVIKEAFD
TPE_isoM1111   SS---KSNIAQAARDYLNMGAVGGDVQTLINWYIAGRD---MPEGNRPRKYKPAPDYFKDVFK---DM--FINQNYIAVQKGIDSIETLEREIKDALE
TPE_isoE1186   --------------------------------------------------------------------------------------------------
TPE_isoM1220   SD---KSDIAQAARSCINFRAAGGDVRTLFNWYISGKN---LPAGQRPNGYKDAPGYFRDIFP---DTTPIVNEQYV------NSKEQLESVIKEAFD
TPE_isoM1224   SS---KSNIAQAARDYLNMGAVGGDVQTLINWYIAGRD---MPEGNRPRKYKPAPDYFKDVFK---DM--FINQNYIAVQKGIDSIETLEREIKDALE
```

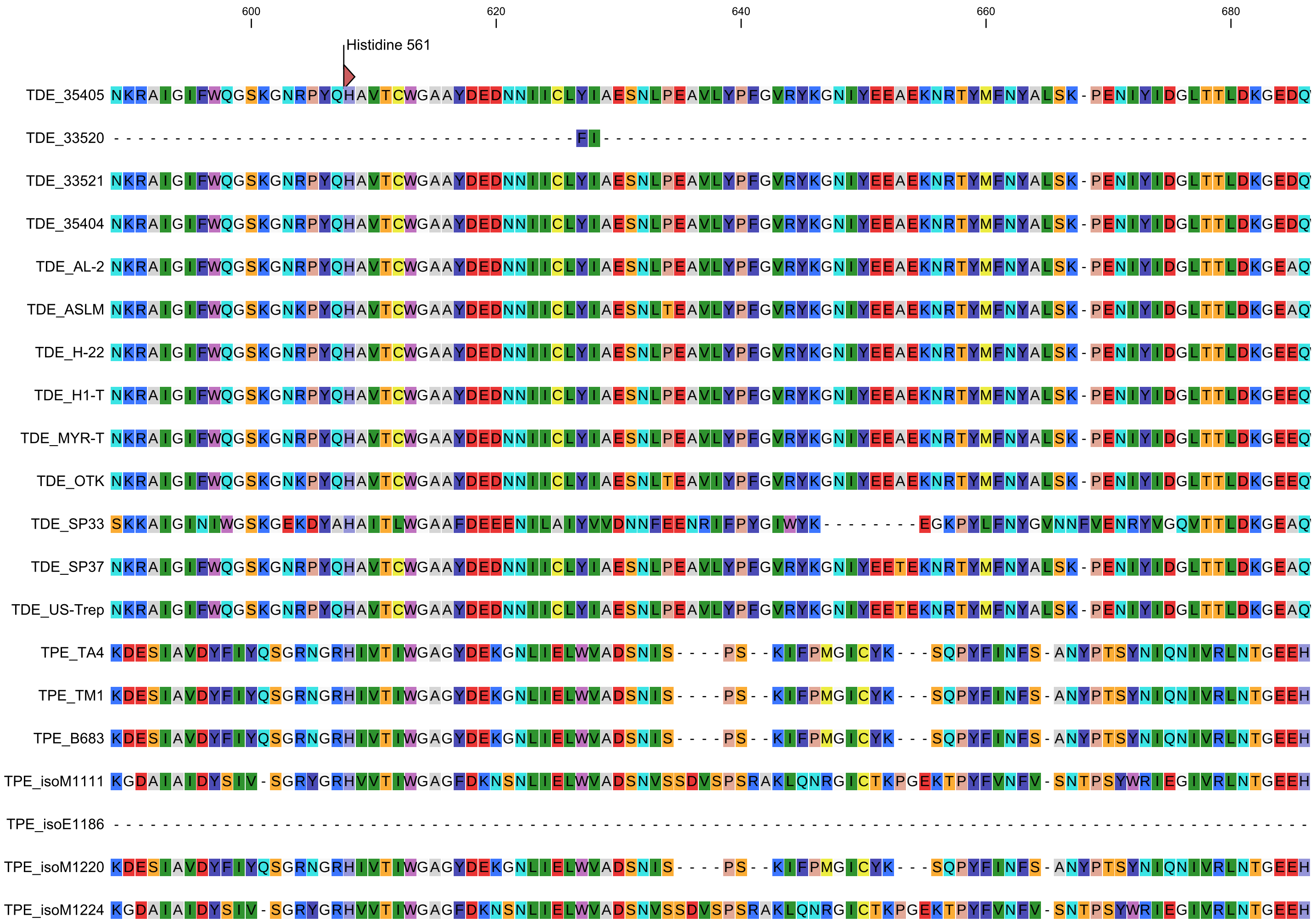
600
620
640
660
680
Histidine 561
TDE_35405
TDE_33520
TDE_33521
TDE_35404
TDE_AL-2
TDE_ASLM
TDE_H-22
TDE_H1-T
TDE_MYR-T
TDE_OTK
TDE_SP33
TDE_SP37
TDE_US-Trep
TPE_TA4
TPE_TM1
TPE_B683
TPE_isoM1111
TPE_isoE1186
TPE_isoM1220
TPE_isoM1224

TDE_35405	WKKWLEAHQ-	647
TDE_33520	----------	463
TDE_33521	WKKWLEAHQ-	647
TDE_35404	WKKWLEAHQ-	647
TDE_AL-2	WKKWLEAHQ-	635
TDE_ASLM	WKKWLEAHQ-	661
TDE_H-22	WKKWLEAHQ-	647
TDE_H1-T	WKKWLEAHQ-	661
TDE_MYR-T	WKKWLEAHQ-	661
TDE_OTK	WKNWLEAHQ-	661
TDE_SP33	WKEWLDKNK-	640
TDE_SP37	WKKWLEAHQ-	635
TDE_US-Trep	WKKWLEAHQ-	508
TPE_TA4	FRKYFENKK*	541
TPE_TM1	FRKYFENKK-	540
TPE_B683	FKKYFENKK-	540
TPE_isoM1111	FKKYFENKK-	364
TPE_isoE1186	----------	284
TPE_isoM1220	FRKYFENKK-	540
TPE_isoM1224	FKKYFENKK-	541
