# Supplementary material for: Genome-Wide Relatedness of Treponema pedis, from Gingiva and Necrotic Skin Lesions of Pigs, with the Human Oral Pathogen Treponema denticola
Source: PLoS One. 2013 Aug 19;8(8):e71281. doi: 10.1371/journal.pone.0071281 (PMC3747143; doi:10.1371/journal.pone.0071281)
Supplement: Figure S4 — Amino acid alignments of T. denticola ATCC 35405 protease PrtP (TDE0762) and identified homologues. Described catalytic residues are indicated along with their corresponding positions in TDE0762. (PDF) [file pone.0071281.s004.pdf]

```
                       20                  40                  60                  80
                        |                   |                   |                   |
TDE_35405     -------MKKKILFFLIAIALIVSSCNFGMNTAVIGNKDNGTVLNGYTGGSGNSSSIELPNGANYKPDDKDIVDGYFIVKTKDGFDKTLFEKKGFIVE
TDE_33520     LNKEVFKMKKKILFFLMTIVLIVSSCNFGMNTAVIGNKDNGAVLNGYTGGLGNSSSVELPNGANYKPDDKDIVDGYFIVKTKDGFDKTLFEKKGFIVK
TDE_33521     LNKEVFKMKKKILFFLIAIALIVSSCNFGMNTAVIGNKDNGTVLNGYTGGSGNSSSIELPNGANYKPDDKDIVDGYFIVKTKDGFDKTLFEKKGFIVE
TDE_35404     LNKEVFKMKKKILFFLIAIALIVSSCNFGMNTAVIGNKDNGTVLNGYTGGSGNSSSIELPNGANYKPDDKDIVDGYFIVKTKDGFDKTLFEKKGFIVE
TDE_AL-2      -------MKKKILFFLVTIVLIVSSCNFGMNTAVIGNKDNGTVLNGYAGGSGNSSSVELPNGANYKPDDKDIVDGYFIVKTKDGFDKTLFEKKGFIVE
TDE_ASLM      -------MKKKILFFLMTIVLIVSSCNFGMNTAVIGNKDNGAVLNGYTGGLGNSSSVELPNGANYKPDDKDIVDGYFIVKTKDGFDKTLFEKKGFIVK
TDE_H-22      -------MKKKILFFLMTIVLIVSSCNFGMNTAVIGNKDNGTVLNGYTGGSGNSSSIELPNGANYKPDDNDIVEGYFIVKTKDGFDKTLFEKKGFIVK
TDE_H1-T      -------MKKKILFFLMTIVLIVSSCNFGMNTAVIGNKDNGAVLNGYTGGLGNSSSVELPNGANYKPDDKDIVDGYFIVKTKDGFDKTLFEKKGFIVK
TDE_MYR-T     ------------------------------------------------------------------------------------LTRLFLXKKGFIVK
TDE_OTK       LNKEVFKMKKKILFFLIAIALIVSSCNFGMNTAVIGNKDNGTVLNGYTGGSGNSSSIELPNGANYKPDDNDIVEGYFIVKTKDGFDKTLFEKKGFIVE
TDE_SP37      -------MKKKILFFLMTIVLIVSSCNFGMNTAVIGNKDNGAVLNGYTGGSGNSSSVELPNGANYEPDDKDIVDGYFIVKTKDGFDKTLFEKKGFIVE
TDE_US-Trep   -------MKKKILFFLMTIVLIVSSCNFGMNTAVIGNKDNGAVLNGYTGGSGNSSSVELPNGANYEPDDKDIVDGYFIVKTKDGFDKTLFEKKGFIVE
TPE_TA4       --------MKKILVLSAVLAILAGSCSFNIDPQNISSNEQR--VQSMEALYGNSSSV-----IPYAPKDEDTVDGFFIVKTKDGFDKTAFEEKGFTVK
TPE_TM1       --------------------------------------------------------------------------------------------------
TPE_B683      --------MKKILVLSAVLAILAGSCSFNIDPQNISSNEQR--VQSMEALYGNSSSV-----LPYAPKDEDTVDGFFIVKTKDGFDKTAFEEKGFTVK
TPE_isoM1111  --------------------------------------------------------------------------------------------------
TPE_isoE1186  --------------------------------------------------------------------------------------------------
TPE_isoM1220  --------MKKILVLSAVLAILAGSCSFNIDPQNISSNEQR--VQSMEALYGNSSSV-----IPYAPKDEDTVDGFFIVKTKDGFDKTAFEEKGFTVK
TPE_isoM1224  --------MKKILVLSAVLAILAGSCSFNIDPQNISSNEQR--VQSMEALYGNSSSV-----LPYAPKDEDTVDGFFIVKTKDGFDKTAFEEKGFTVK
```

```
               100                 120                 140                 160                 180
TDE_35405      GNISLTDTGF-TYWYLNKKGDNKKNLLRAASIEGVLSAEHDYKVVEPDGSKAPNQNDSPVDPSNAGTYGLTDGNYLDDPEANNADYGLSITDALRAYK
TDE_33520      GKISLTDTGF-TYWYLNKEGNNKKNLLRAASIEGVLSAEHDYKVVEPDGSKAPNQNDGPVDPSNAGTYDLTKGNYLDDPEANNADYGLSITDALRAYS
TDE_33521      GNISLTDTGF-TYWYLNKKGDNKKNLLRAASIEGVLSAEHDYKVVEPDGSKAPNQNDSPVDPSNAGTYGLTDGNYLDDPEANNADYGLSITDALRAYK
TDE_35404      GNISLTDTGF-TYWYLNKKGDNKKNLLRAASIEGVLSAEHDYKVVEPDGSKAPNQNDSPVDPSNAGTYGLTDGNYLDDPEANNADYGLSITDALRAYK
TDE_AL-2       GNISLTDTGF-TYWYLNKKGDNKKNLLRAASIEGVLSAEHDYKVVEPDGSKAPNQNDSPVDPSNAGTYGLTDGNYLDDPEANNADYGLSITDALRAYK
TDE_ASLM       GKISLTDTGF-TYWYLNKEGNNKKNLLRAASIEGVLSVEHDYKVVEPDGSKAPNQNDGPVDPFNAETYDLTKGNYLDDPEANNADYGLSITEALKAYS
TDE_H-22       GKISLTDTGF-TYWYLNKKGDNKKNLLRAASIEGVLSAEHDYKVVEPDGSKAPNQNDGPVDPFNAETYDLTKGNYLDDPEANIADYGLSITDALRAYS
TDE_H1-T       GKISLTDTGF-TYWYLNKEGNNKKNLLRAASIEGVLSAEHDYKVVEPDGSKAPNQNDGPVDPSNAGTYDLTKGNYLDDPEANNADYGLSITDALRAYS
TDE_MYR-T      GKISLTDTGFxTYWYLNKEGNNKKNLLRAASIEGVLSAEHDYKVVEPDGSKAPNQNDGPVDPSNAGTYDLTKGNYLDDPEANNADYGLSITDALRAYS
TDE_OTK        GNISLTDTDF-TYWYLNKKGDNKKNLLRAASIEGVLSAEHDYKVVEPDGSKAPNQNDSPVDPSNAGTYGLTDGNYLDDPEANNADYGLSITDALRAYK
TDE_SP37       GNISLTDTGF-TYWYLNKKGDNKKNLLRAASIEGVLSAEHDYKVVEPDGSKAPNQNDSPVDPSNAGAYGLTDGNYLDDPEANNADYGLSITDALRAYK
TDE_US-Trep    GNISLTDTGF-TYWYLNKKGDNKKNLLRAASIEGVLSAEHDYKVVEPDGSKAPNQNDSPVDPSNAGAYGLTDGNYLDDPEANNADYGLSITDALRAYK
TPE_TA4        GALPLTGTGF-TYWYLNKEGNDKKNLSVISSVKGVISAESDYKVEPPDGIKVAKTVDGG---GLVDISRLINGDYSGDPIANNSDYGLSITEALKAYK
TPE_TM1        --------------------------------------------------------------------------------------------------
TPE_B683       GALPLTGTGF-TYWYLNKEGNDKKNLSVISSVKGVISAESDYKVEPPDGIKVAKTVVGG---GLVDISRLINGDYSGDPIANNSDYGLSITEALKAYK
TPE_isoM1111   --------------------------------------------------------------------------------------------------
TPE_isoE1186   --------------------------------------------------------------------------------------------------
TPE_isoM1220   GALPLTGTGF-TYWYLNKEGNDKKNLSVISSVKGVISAESDYKVEPPDGIKVAKTVDGG---GLVDISRLINGDYSGDPIANNSDYGLSITEALKAYK
TPE_isoM1224   GALPLTGTGF-TYWYLNKEGNDKKNLSVISSVKGVISAESDYKVGPPDGIKVAKTVDGG---GLADISRLINGDYSGDPIANNSDYGLSITEALKSYK
```

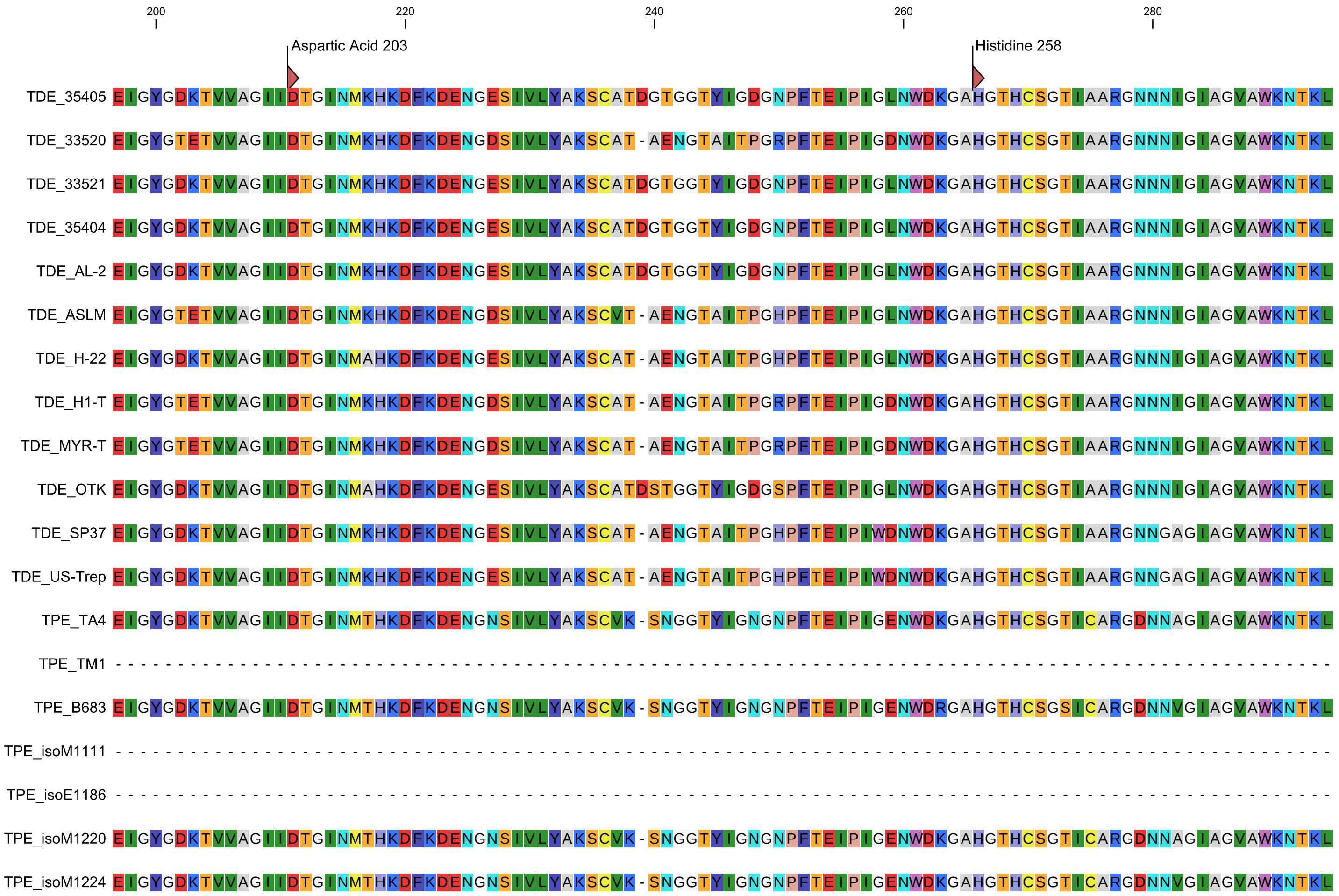
200
220
240
260
280
Aspartic Acid 203
Histidine 258
TDE_35405 EIGYGDKTVVAGIIDTGINMKHKDFKDENGESIVLYAKSCATDGTGGTYIGDGNPFTEIPIGLNWDKGAHGTHCSGTIAARGNNNIGIAGVAWKNTKL
TDE_33520 EIGYGTETVVAGIIDTGINMKHKDFKDENGDSIVLYAKSCAT-AENGTAITPGRPFTEIPIGDNWDKGAHGTHCSGTIAARGNNNIGIAGVAWKNTKL
TDE_33521 EIGYGDKTVVAGIIDTGINMKHKDFKDENGESIVLYAKSCATDGTGGTYIGDGNPFTEIPIGLNWDKGAHGTHCSGTIAARGNNNIGIAGVAWKNTKL
TDE_35404 EIGYGDKTVVAGIIDTGINMKHKDFKDENGESIVLYAKSCATDGTGGTYIGDGNPFTEIPIGLNWDKGAHGTHCSGTIAARGNNNIGIAGVAWKNTKL
TDE_AL-2 EIGYGDKTVVAGIIDTGINMKHKDFKDENGESIVLYAKSCATDGTGGTYIGDGNPFTEIPIGLNWDKGAHGTHCSGTIAARGNNNIGIAGVAWKNTKL
TDE_ASLM EIGYGTETVVAGIIDTGINMKHKDFKDENGDSIVLYAKSCVT-AENGTAITPGHPFTEIPIGLNWDKGAHGTHCSGTIAARGNNNIGIAGVAWKNTKL
TDE_H-22 EIGYGDKTVVAGIIDTGINMAHKDFKDENGESIVLYAKSCAT-AENGTAITPGHPFTEIPIGLNWDKGAHGTHCSGTIAARGNNNIGIAGVAWKNTKL
TDE_H1-T EIGYGTETVVAGIIDTGINMKHKDFKDENGDSIVLYAKSCAT-AENGTAITPGRPFTEIPIGDNWDKGAHGTHCSGTIAARGNNNIGIAGVAWKNTKL
TDE_MYR-T EIGYGTETVVAGIIDTGINMKHKDFKDENGDSIVLYAKSCAT-AENGTAITPGRPFTEIPIGDNWDKGAHGTHCSGTIAARGNNNIGIAGVAWKNTKL
TDE_OTK EIGYGDKTVVAGIIDTGINMAHKDFKDENGESIVLYAKSCATDSTGGTYIGDGSPFTEIPIGLNWDKGAHGTHCSGTIAARGNNNIGIAGVAWKNTKL
TDE_SP37 EIGYGDKTVVAGIIDTGINMKHKDFKDENGESIVLYAKSCAT-AENGTAITPGHPFTEIPIWDNWDKGAHGTHCSGTIAARGNNGAGIAGVAWKNTKL
TDE_US-Trep EIGYGDKTVVAGIIDTGINMKHKDFKDENGESIVLYAKSCAT-AENGTAITPGHPFTEIPIWDNWDKGAHGTHCSGTIAARGNNGAGIAGVAWKNTKL
TPE_TA4 EIGYGDKTVVAGIIDTGINMTHKDFKDENGNSIVLYAKSCVK-SNGGTYIGNGNPFTEIPIGENWDKGAHGTHCSGTICARGDNNAGIAGVAWKNTKL
TPE_TM1 --
TPE_B683 EIGYGDKTVVAGIIDTGINMTHKDFKDENGNSIVLYAKSCVK-SNGGTYIGNGNPFTEIPIGENWDRGAHGTHCSGSICARGDNNVGIAGVAWKNTKL
TPE_isoM1111 --
TPE_isoE1186 --
TPE_isoM1220 EIGYGDKTVVAGIIDTGINMTHKDFKDENGNSIVLYAKSCVK-SNGGTYIGNGNPFTEIPIGENWDKGAHGTHCSGTICARGDNNAGIAGVAWKNTKL
TPE_isoM1224 EIGYGDKTVVAGIIDTGINMTHKDFKDENGNSIVLYAKSCVK-SNGGTYIGNGNPFTEIPIGENWDKGAHGTHCSGTICARGDNNVGIAGVAWKNTKL

```
                300                 320                 340                 360                 380
TDE_35405     ISYQSL--GVEGS--GRTWSVYGAMADLTKIVKILRKPKANRSVAENNALPSYLQNTDFQITQETVPVNMSLGGSYGSEFAFAVLTNAVKHNILPVIA
TDE_33520     ISYQSLNAGLNGQFGGDTWSVYGALLELTNIVEILRKAKADRSLAEIAALPSYLQTTDFQITQKTVPVNMSLGGSYGTEFAFAVLTNAVKHNILPVIA
TDE_33521     ISYQSL--GVEGS--GRTWSVYGAMADLTKIVKILRKPKANRSVAENNALPSYLQNTDFQITQETVPVNMSLGGSYGSEFAFAVLTNAVKHNILPVIA
TDE_35404     ISYQSL--GVEGS--GRTWSVYGAMADLTKIVKILRKPKANRSVAENNALPSYLQNTDFQITQETVPVNMSLGGSYGSEFAFAVLTNAVKHNILPVIA
TDE_AL-2      ISYQSL--GVEGS--GRTWSVYGAMADLTKIVKILRKPKANRSVAENNALPSYLQNTDFQITQETVPVNMSLGGSYGSEFAFAVLTNAVKHNILPVIA
TDE_ASLM      ISYQSLNAGLNGQFGGDTWSVYGALLDLTNIVEILRKAKADRSPAEIAALPSYLQTTDFQITQKTVPVNMSLGGSYGSEFAFAVLTNAVKHNILPVIA
TDE_H-22      ISYQSLNAGLNGQFGGDTWSVYGALLDLTNIVEILRKAKADRSLTEIAALPSYLQTTDFQITQKTVPVNMSLGGSYGSEFAFAVLTNAVKHNILPVIA
TDE_H1-T      ISYQSLNAGLNGQFGGDTWSVYGALLELTNIVEILRKAKADRSPAEIAALPSYLQTTDFQITQKTVPVNMSLGGSYGTEFAFAVLTNAVKHNILPVIA
TDE_MYR-T     ISYQSLNAGLNGQFGGDTWSVYGALLELTNIVEILRKAKADRSPAEIAALPSYLQTTDFQITQKTVPVNMSLGGSYGTEFAFAVLTNAVKHNILPVIA
TDE_OTK       ISYQSL--GVKG--GGRTWSVYGAMADLTKIVKILRKPKANRSVAENNALPSYLQNTDFQITQETVPVNMSLGGSYGTEFAFAILTEAVQNGILPVIA
TDE_SP37      ISYQSLNAGLNGQFGGDTWSVYGALLELTNIVEILRKAKADRSPAEIAALPSYLQTTDFQITQKTVPVNMSLGGSYGTEFAFAVLTNAVKHNILPVIA
TDE_US-Trep   ISYQSLNAGLNGQFGGDTWSVYGALLELTNIVEILRKAKADRSPAEIAALPSYLQTTDFQITQKTVPVNMSLGGSYGTEFAFAVLTNAVKHNILPVIA
TPE_TA4       ISYQSL----DVDGGGSAWAVYGALADLTRTVNILRKPKSDRTLDENNALPSYLKNEDFQITQKTVPVNMSLGGSYGTEFAFSVLTAAVKNNILPVIA
TPE_TM1       --------------------------------------------------------------------------------------------------
TPE_B683      ISYQSL----DVDGGGSAWAVYGALADLTRTVNILRKPKSDRTLDENNALPSYLKNEDFQITQKTVPVNMSLGGSYGTEFAFSVLTAAVKNNILPVIA
TPE_isoM1111  ----------------------------------LRKPKSDRTLDENNALPSYLKNEDFQITQKTVPVNMSLGGSYGTEFAFSVLTAAVKNNILPVIA
TPE_isoE1186  -------------------------------------------------LPSYLKNEDFQITQKTVPVNMSLGGSYGTEFAFSVLTAAVKNNILPVIA
TPE_isoM1220  ISYQSL----DVDGGGSAWAVYGALADLTRTVNILRKPKSDRTLDENNALPSYLKNEDFQITQKTVPVNMSLGGSYGTEFAFSVLTAAVKNNILPVIA
TPE_isoM1224  ISYQSL----DVDGGGSAWAVYGALADLTRTVNILRKPKSDRTLDENNALPSYLKNEDFQITQKTVPVNMSLGGSYGTEFAFSVLTAAVKNNILPVIA
```

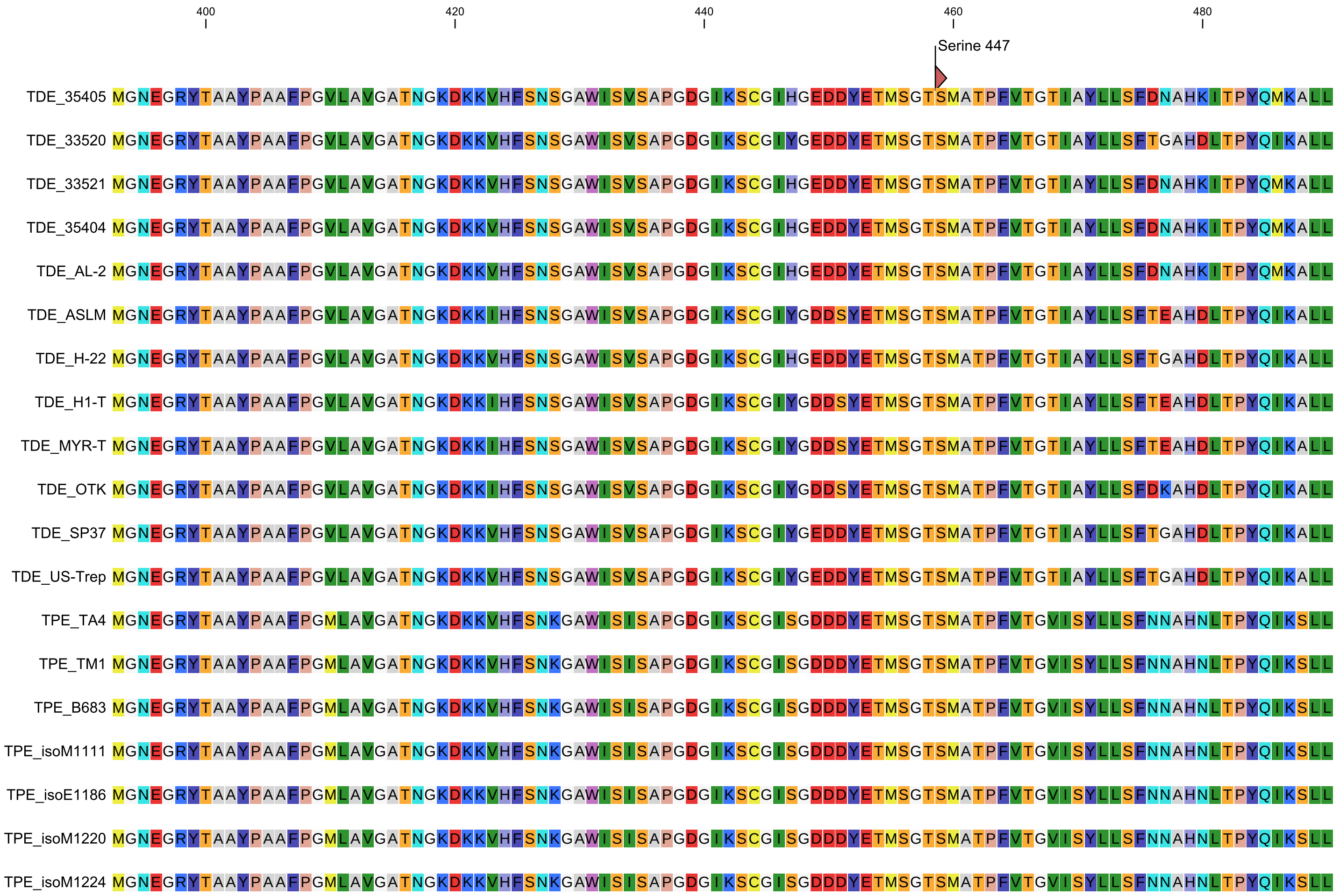
400
420
440
460
480
Serine 447
TDE_35405 MGNEGRYTAAYPAAFPGVLAVGATNGKDKKVHFSNSGAWISVSAPGDGIKSCGIHGEDDYETMSGTSMATPFVTGTIAYLLSFDNAHKITPYQMKALL
TDE_33520 MGNEGRYTAAYPAAFPGVLAVGATNGKDKKVHFSNSGAWISVSAPGDGIKSCGIYGEDDYETMSGTSMATPFVTGTIAYLLSFTGAHDLTPYQIKALL
TDE_33521 MGNEGRYTAAYPAAFPGVLAVGATNGKDKKVHFSNSGAWISVSAPGDGIKSCGIHGEDDYETMSGTSMATPFVTGTIAYLLSFDNAHKITPYQMKALL
TDE_35404 MGNEGRYTAAYPAAFPGVLAVGATNGKDKKVHFSNSGAWISVSAPGDGIKSCGIHGEDDYETMSGTSMATPFVTGTIAYLLSFDNAHKITPYQMKALL
TDE_AL-2 MGNEGRYTAAYPAAFPGVLAVGATNGKDKKVHFSNSGAWISVSAPGDGIKSCGIHGEDDYETMSGTSMATPFVTGTIAYLLSFDNAHKITPYQMKALL
TDE_ASLM MGNEGRYTAAYPAAFPGVLAVGATNGKDKKIHFSNSGAWISVSAPGDGIKSCGIYGDDSYETMSGTSMATPFVTGTIAYLLSFTEAHDLTPYQIKALL
TDE_H-22 MGNEGRYTAAYPAAFPGVLAVGATNGKDKKVHFSNSGAWISVSAPGDGIKSCGIHGEDDYETMSGTSMATPFVTGTIAYLLSFTGAHDLTPYQIKALL
TDE_H1-T MGNEGRYTAAYPAAFPGVLAVGATNGKDKKIHFSNSGAWISVSAPGDGIKSCGIYGDDSYETMSGTSMATPFVTGTIAYLLSFTEAHDLTPYQIKALL
TDE_MYR-T MGNEGRYTAAYPAAFPGVLAVGATNGKDKKIHFSNSGAWISVSAPGDGIKSCGIYGDDSYETMSGTSMATPFVTGTIAYLLSFTEAHDLTPYQIKALL
TDE_OTK MGNEGRYTAAYPAAFPGVLAVGATNGKDKKIHFSNSGAWISVSAPGDGIKSCGIYGDDSYETMSGTSMATPFVTGTIAYLLSFDKAHDLTPYQIKALL
TDE_SP37 MGNEGRYTAAYPAAFPGVLAVGATNGKDKKVHFSNSGAWISVSAPGDGIKSCGIYGEDDYETMSGTSMATPFVTGTIAYLLSFTGAHDLTPYQIKALL
TDE_US-Trep MGNEGRYTAAYPAAFPGVLAVGATNGKDKKVHFSNSGAWISVSAPGDGIKSCGIYGEDDYETMSGTSMATPFVTGTIAYLLSFTGAHDLTPYQIKALL
TPE_TA4 MGNEGRYTAAYPAAFPGMLAVGATNGKDKKVHFSNKGAWISISAPGDGIKSCGISGDDDYETMSGTSMATPFVTGVISYLLSFNNAHNLTPYQIKSLL
TPE_TM1 MGNEGRYTAAYPAAFPGMLAVGATNGKDKKVHFSNKGAWISISAPGDGIKSCGISGDDDYETMSGTSMATPFVTGVISYLLSFNNAHNLTPYQIKSLL
TPE_B683 MGNEGRYTAAYPAAFPGMLAVGATNGKDKKVHFSNKGAWISISAPGDGIKSCGISGDDDYETMSGTSMATPFVTGVISYLLSFNNAHNLTPYQIKSLL
TPE_isoM1111 MGNEGRYTAAYPAAFPGMLAVGATNGKDKKVHFSNKGAWISISAPGDGIKSCGISGDDDYETMSGTSMATPFVTGVISYLLSFNNAHNLTPYQIKSLL
TPE_isoE1186 MGNEGRYTAAYPAAFPGMLAVGATNGKDKKVHFSNKGAWISISAPGDGIKSCGISGDDDYETMSGTSMATPFVTGVISYLLSFNNAHNLTPYQIKSLL
TPE_isoM1220 MGNEGRYTAAYPAAFPGMLAVGATNGKDKKVHFSNKGAWISISAPGDGIKSCGISGDDDYETMSGTSMATPFVTGVISYLLSFNNAHNLTPYQIKSLL
TPE_isoM1224 MGNEGRYTAAYPAAFPGMLAVGATNGKDKKVHFSNKGAWISISAPGDGIKSCGISGDDDYETMSGTSMATPFVTGVISYLLSFNNAHNLTPYQIKSLL

```
                   500                 520                 540                 560                 580
TDE_35405      EKTADKVDGATDFTDSLGHGRVNVYKAAKAIKDGNIPAPNDIYTEAEVTVTVKNNDGHSNDIVPCKITLVDEETHAPLAYVA--------GTGSNPPA
TDE_33520      EKTADKVDGATGFTDRLGHGRVNVYKAAKAIKDGNIPAQNDIYTEAEVTVTVKNNDGHSNDIVPCKITLVDEETHAPLAYVA--------GTGSNPPA
TDE_33521      EKTADKVDGATDFTDSLGHGRVNVYKAAKAIKDGNIPAPNDIYTEAEVTVTVKNNDGHSNDIVPCKITLVDEETHAPLAYVA--------GTGSNPPA
TDE_35404      EKTADKVDGATDFTDSLGHGRVNVYKAAKAIKDGNIPAPNDIYTEAEVTVTVKNNDGHSNDIVPCKITLVDEETHAPLAYVA--------GTGSNPPA
TDE_AL-2       EKTADKVDGATDFTDSLGHGRVNVYKAAKAIKDGNIPAPNDIYTEAEVTVTVKNNDGHSNDIVPCKITLVDEETHAPLAYVA--------GTGSNPPA
TDE_ASLM       EKTADKVDGATGFTDSLGHGRVNVYKAAKAIKDGNIPPANDIYTEAEVKVEVKNNDGHSNDIVPCKITLVDEETHAPLAYVA--------GDESNPPA
TDE_H-22       EKTADKVDGATGFTDSLGHGRVNVYKAAKAIKDGNIPAQNDIYTEAKVTVTVKNNDGHSNDDVPCKITLVDEETHAPLAYVSLAPVSLAPVAGVENPA
TDE_H1-T       EKTADKVDGATGFTDSLGHGRVNVYKAAKAIKDNNIPPENGVYTEKDVKVKVTNDDGFGVEVVPCKITLVDEVTHAPLAYVA--------GLGNTPIT
TDE_MYR-T      EKTADKVDGATGFTDSLGHGRVNVYKAAKAIKDNNIPPENGVYTEKDVKVKVTNDDGFGVEVVPCKITLVDEVTHAPLAYVA--------GLGNTPIT
TDE_OTK        EKTADKVDGATGFTDSLGHGRVNVYKAAKAIKDNNIPPENGLYTEKEVQVKVTNDDGFGVEVVPCKITLVDEVTNAPLAYVS--------GLGNTPIT
TDE_SP37       EKTADKVDGATGFTDRLGHGRVNVYKAAKAIKDGNIPAQNDIYTEAEVTVTVKNNDGHSNDIVPCKITLVDEETHAPLAYVA--------GTGSNPPA
TDE_US-Trep    EKTADKVDGATGFTDRLGHGRVNVYKAAKAIKDGNIPAQNDIYTEAEVTVTVKNNDGHSNDIVPCKITLVDEETHAPLAYVA--------GTGSNPPA
TPE_TA4        EKTADKVDGAVSFTEGYGHGRVNVYNAAKAIRENSIPQVNEIYSEGSVYVEVKNN----NEVIASKISLVDEETKVPLAYVA--------GLGNNPVV
TPE_TM1        EKTADKVDGAVSFTEGYGHGRVNVYNAAKAIRENSIPQVNEIYSEGSVYVEVKNN----NEVIASKISLVDEETKVPLAYVA--------GLGNNPVV
TPE_B683       EKTADKVDGAVSFTEGYGHGRVNVYNAAKAIRENNIPQVNEIYSEGSVYVEVKNN----NEVIASKISLVDEETKVPLAYVA--------GLGNNPVV
TPE_isoM1111   EKTADKVDGAVSFTEGYGHGRVNVYNAAKAIRENNIPQVNEIYSEGSVYVEVKNN----NEVIASKISLVDEETKVPLAYVA--------GLGNNPVV
TPE_isoE1186   EKTADKVDGAVSFTEGYGHGRINVYNAAKAIRENNIPQVNEIYSEGSVYVEVKNN----NEVIASKISLVDEETKVPLAYVA--------GLGNNPVV
TPE_isoM1220   EKTADKVDGAVSFTEGYGHGRVNVYNAAKAIRENSIPQVNEIYSEGSVYVEVKNN----NEVIASKISLVDEETKVPLAYVA--------GLGNNPVV
TPE_isoM1224   EKTADKVDGAVSFTEGYGHGRVNVYNAAKAIRENNIPQVNEIYSEGSVYVEVKNN----NEVIASKISLVDEETKVPLAYVA--------GLGNNPVV
```

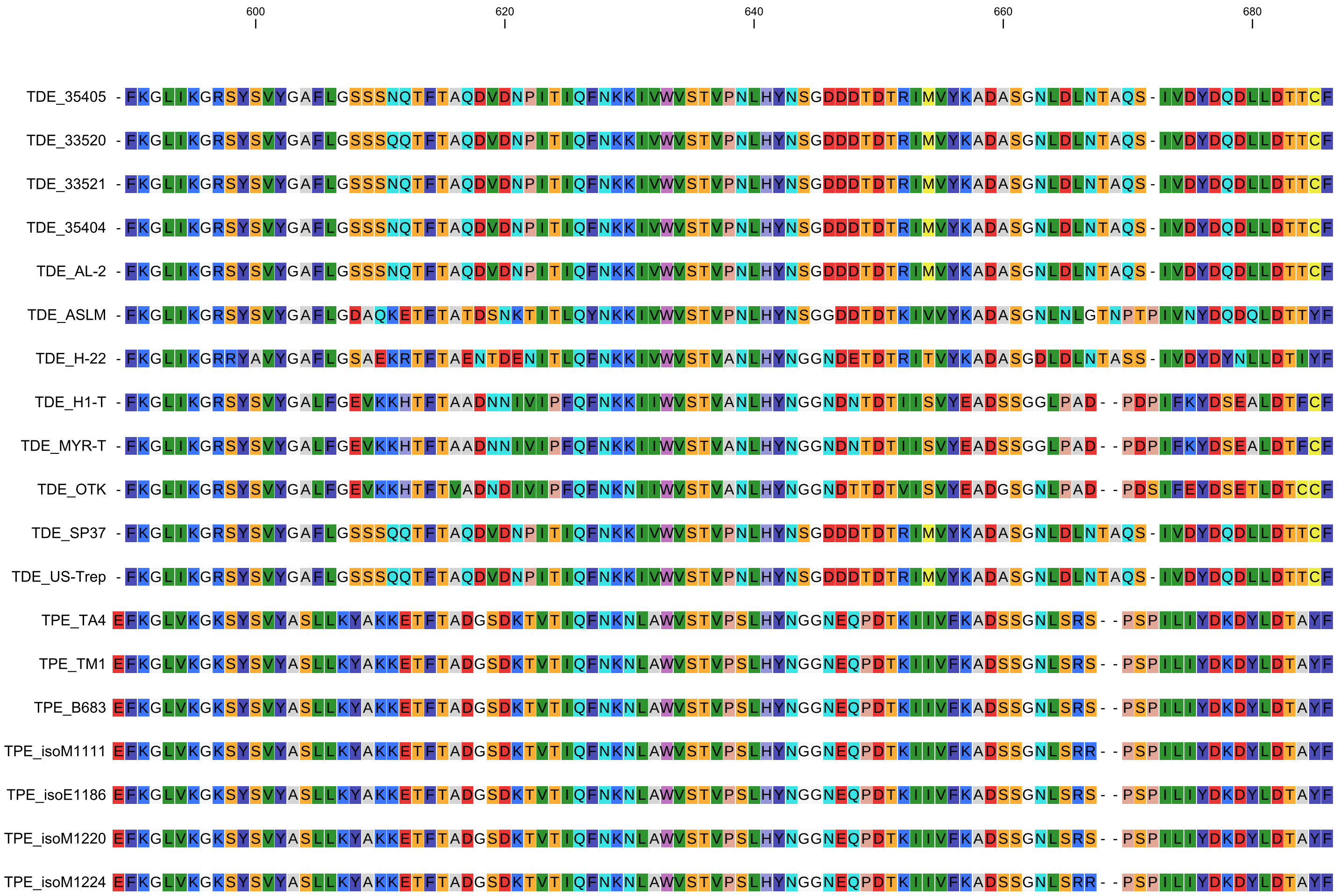
600
620
640
660
680
TDE_35405 -FKGLIKGRSYSVYGAFLGSSSNQTFTAQDVDNPITIQFNKKIVWVSTVPNLHYNSGDDDTDTRIMVYKADASGNLDLNTAQS-IVDYDQDLLDTTCF
TDE_33520 -FKGLIKGRSYSVYGAFLGSSSQQTFTAQDVDNPITIQFNKKIVWVSTVPNLHYNSGDDDTDTRIMVYKADASGNLDLNTAQS-IVDYDQDLLDTTCF
TDE_33521 -FKGLIKGRSYSVYGAFLGSSSNQTFTAQDVDNPITIQFNKKIVWVSTVPNLHYNSGDDDTDTRIMVYKADASGNLDLNTAQS-IVDYDQDLLDTTCF
TDE_35404 -FKGLIKGRSYSVYGAFLGSSSNQTFTAQDVDNPITIQFNKKIVWVSTVPNLHYNSGDDDTDTRIMVYKADASGNLDLNTAQS-IVDYDQDLLDTTCF
TDE_AL-2 -FKGLIKGRSYSVYGAFLGSSSNQTFTAQDVDNPITIQFNKKIVWVSTVPNLHYNSGDDDTDTRIMVYKADASGNLDLNTAQS-IVDYDQDLLDTTCF
TDE_ASLM -FKGLIKGRSYSVYGAFLGDAQKETFTATDSNKTITLQYNKKIVWVSTVPNLHYNSGGDDTDTKIVVYKADASGNLNLGTNPTPIVNYDQDQLDTTYF
TDE_H-22 -FKGLIKGRRYAVYGAFLGSAEKRTFTAENTDENITLQFNKKIVWVSTVANLHYNGGNDETDTRITVYKADASGDLDLNTASS-IVDYDYNLLDTIYF
TDE_H1-T -FKGLIKGRSYSVYGALFGEVKKHTFTAADNNIVIPFQFNKKIIWVSTVANLHYNGGNDNTDTIISVYEADSSGGLPAD--PDPIFKYDSEALDTFCF
TDE_MYR-T -FKGLIKGRSYSVYGALFGEVKKHTFTAADNNIVIPFQFNKKIIWVSTVANLHYNGGNDNTDTIISVYEADSSGGLPAD--PDPIFKYDSEALDTFCF
TDE_OTK -FKGLIKGRSYSVYGALFGEVKKHTFTVADNDIVIPFQFNKNIIWVSTVANLHYNGGNDTTDTVISVYEADGSGNLPAD--PDSIFEYDSETLDTCCF
TDE_SP37 -FKGLIKGRSYSVYGAFLGSSSQQTFTAQDVDNPITIQFNKKIVWVSTVPNLHYNSGDDDTDTRIMVYKADASGNLDLNTAQS-IVDYDQDLLDTTCF
TDE_US-Trep -FKGLIKGRSYSVYGAFLGSSSQQTFTAQDVDNPITIQFNKKIVWVSTVPNLHYNSGDDDTDTRIMVYKADASGNLDLNTAQS-IVDYDQDLLDTTCF
TPE_TA4 EFKGLVKGKSYSVYASLLKYAKKETFTADGSDKTVTIQFNKNLAWVSTVPSLHYNGGNEQPDTKIIVFKADSSGNLSRS--PSPILIYDKDYLDTAYF
TPE_TM1 EFKGLVKGKSYSVYASLLKYAKKETFTADGSDKTVTIQFNKNLAWVSTVPSLHYNGGNEQPDTKIIVFKADSSGNLSRS--PSPILIYDKDYLDTAYF
TPE_B683 EFKGLVKGKSYSVYASLLKYAKKETFTADGSDKTVTIQFNKNLAWVSTVPSLHYNGGNEQPDTKIIVFKADSSGNLSRS--PSPILIYDKDYLDTAYF
TPE_isoM1111 EFKGLVKGKSYSVYASLLKYAKKETFTADGSDKTVTIQFNKNLAWVSTVPSLHYNGGNEQPDTKIIVFKADSSGNLSRR--PSPILIYDKDYLDTAYF
TPE_isoE1186 EFKGLVKGKSYSVYASLLKYAKKETFTADGSDKTVTIQFNKNLAWVSTVPSLHYNGGNEQPDTKIIVFKADSSGNLSRS--PSPILIYDKDYLDTAYF
TPE_isoM1220 EFKGLVKGKSYSVYASLLKYAKKETFTADGSDKTVTIQFNKNLAWVSTVPSLHYNGGNEQPDTKIIVFKADSSGNLSRS--PSPILIYDKDYLDTAYF
TPE_isoM1224 EFKGLVKGKSYSVYASLLKYAKKETFTADGSDKTVTIQFNKNLAWVSTVPSLHYNGGNEQPDTKIIVFKADSSGNLSRR--PSPILIYDKDYLDTAYF

```
                          700                 720                 740                 760                 780
                          |                   |                   |                   |                   |
TDE_35405    EAETGAKYYVLITGFM-KGGVFQGGNYALKIDRTPLNSNGVNIDDTARSPNNASTNDSH---------------------------------------
TDE_33520    EAETGAKYYVLITGFM-KGGVFQGGNYALKIDRTPLNPNGVDIDDTARSPNNASTNDSHED---DDTPAFAKVKGNAWGQTKGCNLVAHPIGSN--NV
TDE_33521    EAETGAKYYVLITGFM-KGGVFQGGNYALKIDRTPLNSNGVNIDDTARSPNNASTNDSHED---DDTPAFAKVKGNAWGQTKGCNLVAHPIGSN--NV
TDE_35404    EAETGAKYYVLITGFM-KGGVFQGGNYALKIDRTPLNSNGVNIDDTARSPNNASTNDSHED---DDTPAFAKVKGNAWGQTKGCNLVAHPIGSN--NV
TDE_AL-2     EAETGAKYYVLITGFM-KGGVFQGGNYALKIDRTPLNSNGVDIDDVGRSPNNASTNDSHEGAGFDDTPADAKSKGNAWGQEMGCNLVAHPIGTPPANV
TDE_ASLM     EAETGAKYYVLITGFM-KGGVFQGGNYALKIDRTPLNSNGVDIDDTARSPNNASTNDSHEGAGFDDTPADAKSKGNAWGQEMGCNLVAHPIGTPPKNV
TDE_H-22     EAETGAKYYVLITGFM-KEGVFQGGNYALKIDRTPLNSNGVDIDDVGRSPNNASTNDSHED---DDTPAFAKVKGDAWGQTKGCNLVAHPIGTPPANV
TDE_H1-T     EAEHGKKYYVKITNY---GPLTGSKNYVISIDRKSL-SGAEDMDD-GRL---ATANDSHEN---DDTPGDAKIKGNAWGQKYACNLVAHPIGGT--NT
TDE_MYR-T    EAEHGKKYYVKITNY---GPLTGSKNYVISIDRKSL-SGAEDMDD-GRL---ATANDSHEN---DDTPGDAKIKGNAWGQKYACNLVAHPIGGT--NT
TDE_OTK      EAEHGKKYYVKITNY---GALTGSKNYVISIDRKSL-SGAEDMDDNGRS---ATANDSHEN---DDTPAGAKLKGNAWGQKYACNLVAHTIGGE--NT
TDE_SP37     EAETGAKYYVLITGFM-KGGVFQGGNYALKIDRTPLNPNGVDIDDTARSPNNASTNDSHED---DDTPAFAKVKGNAWGQTKGCNLVAHPIGSN--NV
TDE_US-Trep  EAETGAKYYVLITGFM-KGGVFQGGNYALKIDRTPLNPNGVDIDDTARSPNNASTNDSHED---DDTPAFAKVKGNAWGQTKGCNLVAHPIGSN--NV
TPE_TA4      EYESGAEYYAEITGLKDEQGIFRGGNYVVKIGLTPLDLNGEDIIDGSRV---ASDNDTHED---DDEPDKAKLKGNAWEKKYACNLAAH--GTN--NE
TPE_TM1      EYESGAEYYAEITGLKDEQGIFRGGNYVVKIGLTPLDLNGEDIIDGSRV---ASDNDTHED---DDEPDKAKLKGNAWEKKYACNLAAH--GTN--NE
TPE_B683     EYESGAEYYTAITGLKDEQGTFRGGNYVVKIGLTPLDLNGEDIIDGSRV---ASDNDTHED---DDESDKAKLKGNAWEKKYACNLAAH--GTN--NE
TPE_isoM1111 EYESGAEYYTAITGLKDERGSFRGGNYVVKIGLTPLDLNGEDIIDGSRV---ASDNDTHED---DDESDKAKLKGNAWEKKYACNLAAH--GTN--NE
TPE_isoE1186 EYESGAEYYAAITGLKDEQGIFRGGNYVVKIGLTSLDLNGEDIIDGSRL---ASDNDTHED---DDESDKAKLKGNAWEKKYACNLAAH--GTN--NE
TPE_isoM1220 EYESGAEYYAEITGLKDEQGIFRGGNYVVKIGLTPLDLNGEDIIDGSRV---ASDNDTHED---DDEPDKAKLKGNAWEKKYACNLAAH--GTN--NE
TPE_isoM1224 EYESGAEYYTAITGLKDERGTFRGGNYVVKIGLTPLDLNGEDIIDDSRV---ASDNDTHED---DDEPDKAKLKGNAWEKKYACNLAAQ--GTN--NE
```

TDE_35405	- - - - - - - - - - -	722
TDE_33520	DLDWFYVEYP-	776
TDE_33521	DLDWFYVEYP-	773
TDE_35404	DLDWFYVEYP-	773
TDE_AL-2	DLDWFYVEYPY	772
TDE_ASLM	DLDWFYVEYPY	776
TDE_H-22	DLDWFYVEYPY	780
TDE_H1-T	DEDWFYVEYP-	761
TDE_MYR-T	DEDWFYVEYP-	685
TDE_OTK	DEDWFYVEYP-	766
TDE_SP37	DLDWFYVEYP-	769
TDE_US-Trep	DLDWFYVEYP-	769
TPE_TA4	DIDFFYIKMP-	746
TPE_TM1	DIDFFYIKMP-	378
TPE_B683	DIDFFYIKTP-	746
TPE_isoM1111	DIDFFYIKTP-	442
TPE_isoE1186	DIDFFYIKTP-	427
TPE_isoM1220	DIDFFYIKMP-	746
TPE_isoM1224	DIDFFYIKTP-	746
